# Insights into long term glass corrosion mechanisms from the Ballidon experiment

**DOI:** 10.1038/s41529-025-00571-0

**Published:** 2025-03-17

**Authors:** C. L. Thorpe, A. J. Fisher, G. Manifold, S. Creasey-Gray, C. M. Jackson, B. Stone, C. L. Corkhill, C. Boothman, J. R. Lloyd, R. J. Hand

**Affiliations:** 1 School of Chemical, Materials, and Biological Engineering, University of Sheffield, Sheffield, UK; 2 Dalton Cumbrian Facility, University of Manchester, Westlakes Science Park, Whitehaven, Cumbria UK; 3 School of History, Philosophy and Digital Humanities, University of Sheffield, Sheffield, UK; 4 School of Earth Sciences, University of Bristol, Bristol, UK; 5 Williamson Research Centre, University of Manchester, Manchester, UK

**Keywords:** Materials science, Geochemistry

## Abstract

At the Ballidon experiment, one of the longest running glass durability studies, modern and simulant archaeological glasses were buried in mildly alkaline, under-saturated, conditions for 52 years. Glass surfaces were analysed to determine the extent and mechanisms of alteration. Alteration layer chemistry was complex and included Ca from the surrounding limestone sediment and P from porewater resulting in Ca, Pb and Fe-phosphate rich phases interspersed with Si and Al rich regions. There was evidence for ongoing evolution of the alteration layer structure due to continued fluid ingress. Lamellae in the silica-rich regions approximately numbering the years of burial and indicating a possible link between their formation and seasonal climate cycling. Comparison of field samples with laboratory dissolution tests highlighted the impact of surface finish on initial alteration rate and the limitations of using alteration layer thickness to estimate the amount of glass that has dissolved.

## Introduction

The Ballidon experiment, established in 1970 to better understand the corrosion of ancient and modern glass types under alkaline conditions, is now one of the world’s longest running glass corrosion studies^1–3^. Since its conception, the experimental focus has been expanded to include the burial of additional borosilicate and iron phosphate glasses, representative of vitrified radioactive wastes from the UK, US and Russia. Here we explore what can be learned from analysis of the nine original glass types after 52 years of burial, with respect to the experiment’s original aims and more recent objectives pertaining to the safe disposal of vitrified radioactive wastes.

Understanding glass alteration is of interest from many perspectives, including the conservation of art and archaeological artefacts^4^, the longevity of construction materials and product components, the behaviour of glasses for biomedical applications (e.g. ref. ^5^) and the durability of vitrified radioactive wastes (e.g. refs. ^6, 7^). Numerous laboratory studies have unpicked the mechanisms by which silicate-based glasses alter when contacted by water or water vapour and describe the successive stages of ion exchange, network hydrolysis, secondary phase formation and accompanying reduction of alteration rate (as well as possible rate resumption)^8^. Despite improved mechanistic understanding, there is still no model that can accurately predict the durability of a given glass composition in every possible environment, in part because of the vast range of possible chemical compositions, but also due to the even greater number of environmental factors to consider. Laboratory tests are usually conducted at elevated temperatures and under closed system (often static), sterile conditions^9^. These simplified systems may exclude potentially important corrosion mechanisms that may have negligible effect over days or weeks but whose cumulative impact may be significant over the long-term. For example, seasonal temperature and rainfall cycling, incorporation of elements from adjacent mineralogy and the actions of microorganisms may all affect glass alteration over long timeframes. The studies of naturally altered glasses from long-term field studies like Ballidon therefore have an important part to play in validating laboratory derived results^10^.

The nine glass types (Table 1) originally buried at the Ballidon site were replicas of an identical set buried in 1963 at Wareham Experimental Earthwork (Morden Bog National Nature Reserve, Wareham St Martin, Dorset, SY/911923), which was itself established following the success of Overton Down Experimental Earthwork (constructed 1960)^11,12^. Both Wareham and Overton Down comprised a large artificial bank of soil above the natural ground surface level and were intended to test the effects of alkali (Overton Down) vs acidic (Wareham) sediment on the corrosion of a variety of manmade, biological and geological materials of interest from the point of view of understanding the corrosion of archaeological artefacts^13^. Due to the exclusion of glasses from the earlier Overton Down experiment^14^, and the expectation that glasses would alter slowly in the mildly acidic soil at Wareham, a separate experiment in alkaline soil was proposed, focused entirely on the behaviour of glass, and was established at Ballidon Quarry, Derbyshire^1^.

**Table 1:** Description of the glasses buried at Wareham and subsequently at Ballidon^1^

Glass Type	Name	Description
1	Roman glass	Newly-made glass of typical Roman composition (high Na and Ca)
2	Medieval glass	Newly-made glass of typical medieval composition (high K and Ca)
3	Hangleton linen smoother	Newly-made glass of composition similar to that of the linen smoother from Hangleton ^73^ (low Si)
4	Polished plate glass	Contemporary plate glass^a^ with both surfaces ground and polished. ‘Pilkington Brothers Ltd’
5	Plate glass ‘as produced’	Contemporary plate glass but with the surfaces left in ‘as produced’ condition. ‘Pilkington Brothers Ltd’
6	E-glass	Marbles of ‘E’ glass composition, i.e. a typical low-alkali glass-fibre material (high Al)
7	Borosilicate	Heat-resistant borosilicate oven-ware glass
8	Soda-lime optical glass	High quality soda-lime optical glass with all surfaces ground flat
9	Lead optical glass	Lead optical glass with all surfaces ground flat (high Pb)

^a^At the time the Wareham experiment was started float glass was still a relatively novel product with plate and sheet glass being the more common flat glass manufacturing routes, although by 1970 float glass had commercially replaced plate glass

In 1963 at Wareham, identical sets of buried materials were emplaced in two contrasting environments (turf and sand bank) in 8 sections to be excavated at intervals. These intervals were 1964 (13.5 months), 1965 (2 years), 1968 (5 years) and 1972 (9 years), 1980 (17 years) and 1996 (33 years) with further retrievals planned for 2027 (64 years) and 2091 (128 years)^15^. Data from the 33-year retrieval showed the pH was 3.8 in the turf environment (approximately 1.3 m below the top of the earthworks in 1996) and 4–4.7 the pH in the sand environment (mid-bank - approximately 0.5 m below the surface of the earthworks and 60 cm above ground level in 1996). The two environments differ in water content, aeration, compressibility, mineral content, and organic matter with the turf stack presenting a continually moist, partially anaerobic environment and the sand presenting a fast draining, easily aerated zone^13^.

The only results from Wareham publically available in the literature are from the 5 and 9 year retrievals and these are summarised in Table 2. Some limited observations were made, for example: “The greater resistance of the glasses of modern type was apparent after 1 year and was maintained, so that after 9 years’ samples 4, 5, 8 and 9 from both turf and sand, and samples 6 and 7 from the sand still showed no signs of attack”^13^. Meanwhile glasses 1, 2, 3 from both environments and glasses 6 and 7 from the sand showed signs of alteration manifesting as the development of iridescence on the glass surface. Further analysis or interpretation was not conducted on the samples from Wareham, partly as glass corrosion was not the main focus of the study and partly as advanced microscopy techniques were expensive and not as widely available in 1972 as they are today.

**Table 2:** Corrosion observed at Wareham after 9 years in acidic soil in 1972

Glass Type	Name	Description of corrosion in sand environment	Description of corrosion in turf environment
1	Roman glass	A slight weathering crust with craters, c. 200 µm thick (after 5 years)	A slight weathering crust with craters, c. 200 µm thick (after 5 years)
2	Medieval glass	Strong blue colouring on the underside. C. 200 µm	More attack than the Roman, with the development of iridescence. A ‘fingerprint’ pattern of whorls, perhaps caused by changes in pH across a broken heather stalk. C. 200 µm
3	Hangleton linen smoother	Increasing iridescence from 5 to 9 years	Increasing iridescence from 5 to 9 years
4	Polished plate glass	No sign of attack	No sign of attack
5	Plate glass ‘as produced’	No sign of attack	No sign of attack
6	E-glass	No sign of attack	Some iridescence (after 5 years)
7	Borosilicate	No sign of attack	Peculiar band of apparent crazing 2–3 mm wide on one surface (after 5 years)
8	Soda-lime optical glass	No sign of attack	No sign of attack
9	Lead optical glass	No sign of attack	No sign of attack

Samples were examined under low and medium power optical microscopes; signs of alteration were noted and colour photographs and photomicrographs were taken (photographs recorded are available at the British Glass Manufacturers Research Association (now the British Glass Manufacturers Confederation) and lists are kept in the Earthwork Archive)

**Fig. 1:**
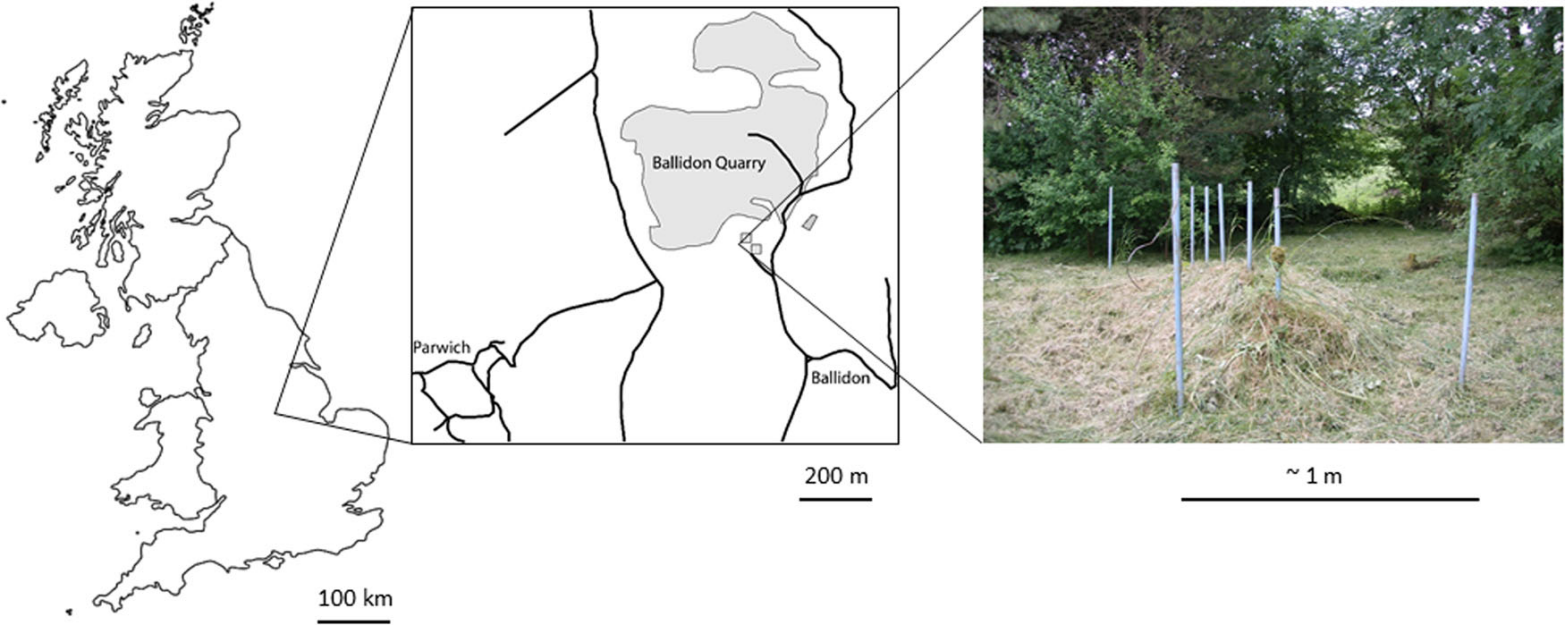
Location and appearance of the Ballidon glass burial experiment in summer 2022.

**Fig. 2:**
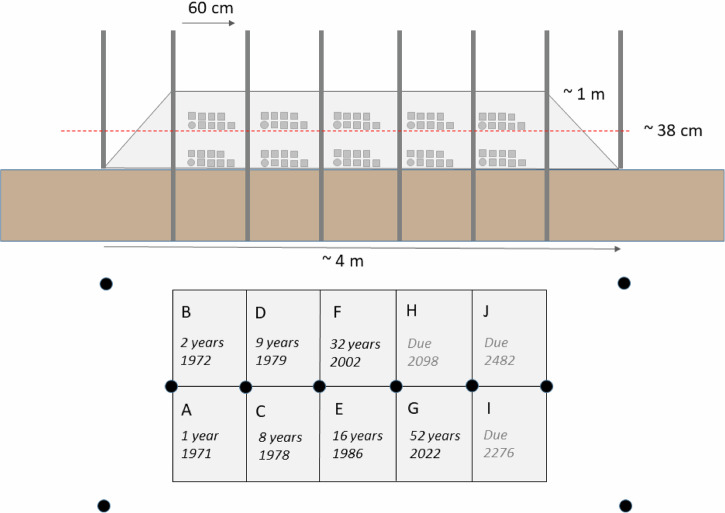
Diagram of the Ballidon earthwork. Above: Schematic cross section of the earthwork at Ballidon showing the original burial depth of the ‘mid mound’ samples (red dashed line). Below: Plan view of the earthwork showing the location of the ten sets of samples and their excavation/projected excavation dates.

The Ballidon experiment was established at ‘Ballidon Quarry’, Ballidon, Derbyshire, DE6 1QX, Lat. 53.088 Long. −1.700, in 1970 (Fig. 1). A smaller ‘earthwork’ to that at Wareham, it was constructed measuring approximately 3.5 m by 1.5 m and originally around 1 m in height. Steel poles were driven into the ground to mark out a grid of ten sections with 18 samples buried in each section (9 at the base of the bank in contact with the topsoil and 9 in the middle of the bank) (Fig. 2). The bank was constructed from material described as ‘limestone chatter’, pieces of crushed rock of varying sizes (<1–8 cm) sourced from the adjacent Ballidon Quarry (currently owned by Tarmac Ltd). The original pH of the mound was stated (in 1970) to be pH 9.5–9.7 although there is no record of this being checked at subsequent excavations which took place in 1971 (1 year), 1972 (2 years), 1978 (8 years), 1979 (9 years), 1986 (16 years) and 2002 (32 years). The pH was next measured in the 2022 (52 years) excavation and was found to have decreased to between 7.8–8.2.

Analysis from early sample points (16 and 32 years) was limited to optical microscopy (e.g. refs. ^16–19^), with Mcloughlin et al.^3^ the first to analyse sample alteration layers using scanning electron microscopy (SEM), though only reporting on three of the nine glasses. As these past studies are either incomplete, or the results unclear, a brief summary of the results are provided in Table 3. This current study will be treated as the first complete investigation of the Ballidon samples using high resolution imaging techniques, which includes an assessment of the chemical durability of pristine Ballidon glasses using the short-term Stirred Reactor Coupon Analysis (SRCA) protocol, ASTM C1925^20^.

**Table 3:** Summary of the alteration observed on the Ballidon glasses after 16 and 32 years in alkaline soil

Glass Type	Name	Description of alteration after 1–16 years^3,18^	Description of alteration after 32 years^3,74^
1	Roman glass	No sign of attack in initial study. Sodium leaching observed in 2006 study of 1 year (2 µm) and 9 year (6 µm) samples^3^.	Sodium leaching 10–20 µm and dissolution of silica 2–5 µm^3^.
2	Medieval glass	Iridescence. 2006 analysis reveals a 5–20 µm present for 1 year and 9 year samples^3^.	Calcium rich corrosion layer 10–15 µm^3^.
3	Hangleton linen smoother	2006 analysis reveals 2–20 µm alteration after 1 year and up to 40 µm after 9 years^3^. 16-year sample showed extensively crazed surface estimated 260 µm. Ca-rich alteration layer^18^.	Not reported
4	Polished plate glass	No sign of attack	Not reported
5	Plate glass ‘as produced’	No sign of attack	Not reported
6	E-glass	No sign of attack	Not reported
7	Borosilicate	No sign of attack	Not reported
8	Soda-lime-silica optical glass	No sign of attack	Not reported
9	Lead optical glass	No sign of attack	Not reported

Newton et al.^18^, used optical microscopy whilst McLoughlin et al.^3^ used SEM with energy dispersive x-ray (EDX) analysis. This later study also re-examined 1 and 9 year samples from glasses 1, 2 and 3

As samples were removed from the Ballidon experiment, other samples were emplaced in the then vacant sections of the earthwork. These included a two-year experiment on ten archaeological compositions (established by the European Science Foundation 1980–1982;^17,18^); two experiments to test the durability of US nuclear waste glass samples (established by researchers at Savannah River National Laboratory 1986–2002 and 2002–2022^21–23^; and various nuclear waste glass compositions including UK and Russian glasses (emplaced by the University of Sheffield 2002–2022). This manuscript concerns the nine glass types included in the original Ballidon experiment as buried in 1970. However, subsequent manuscripts (e.g. Manifold et al. in prep.) will discuss the alteration of nuclear waste type glasses removed from the Ballidon site in 2022. A short documentary about the Ballidon experiment was prepared following the removal of 35 glass samples in 2022 (available at: https://youtu.be/6-N81zqLBnE).

## Results

### Site characterisation

**Fig. 3:**
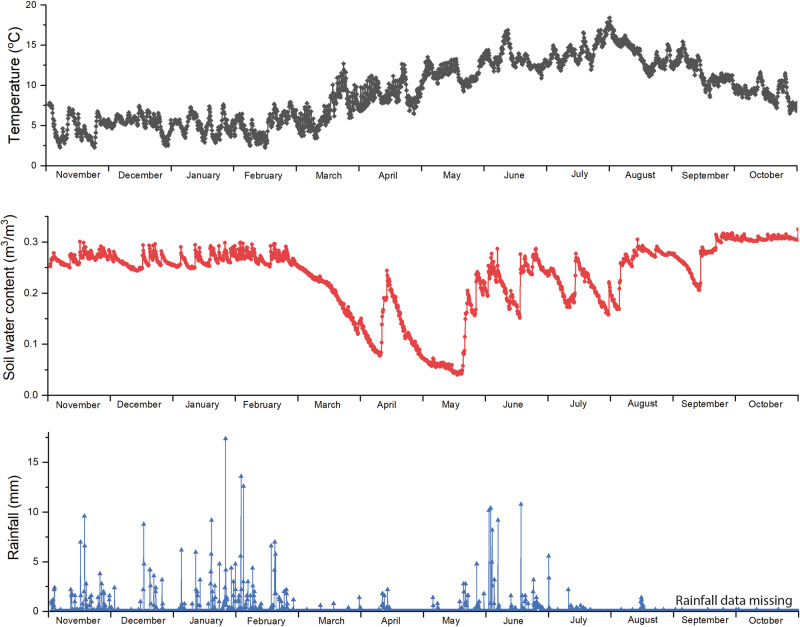
Temperature, soil water content, and rainfall data gathered from the Ballidon site. Data was collected over the course of one year (November 2019–November 2020). The lack of rainfall after August 2020 is due to a fault with the rainfall monitor, rainfall during this period can be inferred from nearby weather stations (Supplementary Fig. 7).

The Ballidon earthwork was monitored for 12 months between November 2019 and November 2020 (Fig. 3) to log rainfall, temperature and soil saturation (conductivity). In addition, data collected at Ballidon was compared with average soil moisture readings and publically available rainfall data compiled from local weather stations for 1970–2022 (summarised in Supplementary Figs. 1–9)^24–26^. Onsite monitoring showed that, during the winter months of October-March, the soil water content remained relatively stable (between 0.25 and 0.3 m^3^/m^3^). During the summer months, April – September, onsite monitoring showed that saturation varied between 0.05 and 0.3 m^3^/m^3^ meaning that there were periods where the sediment around the samples, and especially around those located ‘mid-mound’, would have been almost completely dry. As expected, these periods of low soil water content corresponded with months of low rainfall which in 2020 occurred in March, April and the first half of May. On-site monitoring showed the earthworks took 4–6 weeks to dry out after rainfall and, therefore, weather data from 1970–2022 was studied to identify similar periods of dry weather. Seventeen such periods were identified where there was no (or negligible) rainfall for four weeks or more during the spring-summer months (March–October). Based on these criteria it was proposed that the earthwork is unlikely to have dried out completely every year but can be considered an unsaturated environment and this must be taken into account when considering glass corrosion rates and mechanisms. When glass corrosion layers dry out, they can crack and often separate a little from the pristine glass on which they have formed. Wetting and drying over a 52-year period at Ballidon will likely have resulted in cracks and channels by which water could reach the pristine glass without requiring diffusion through any alteration layer formed^27^.

The temperature logger, located ~15 cm beneath the soil surface recorded temperatures between 2 and 18 °C (averaging 9.2 °C) confirming that soil in contact with the samples did not experience temperatures below 0 °C during the monitored year. During this period, temperatures for the UK were overall above the 1961–2022 long-term average; by 1–1.5 °C. Prolonged cold snaps at earlier time-periods may have led to frost reaching the sample depth at times within the 52-year time period, however, it is reasonable to assume that this has become increasingly rare and that the effect of freeze-thaw is probably not a major mechanism for physical weathering of the sample.

The pH of the soil measured at the time of sample removal (June 2022) was 7.8–8.2 (Table 4), lower than the pH of 9.5–9.7 recorded by Fletcher. Assuming that this initial value was reported accurately, the difference can likely be attributed to weathering by continuous rainfall resulting in soil neutralisation (via leaching of base cations (e.g. Ca, Mg and K), and potentially to the growth of plant matter, and associated microorganisms, on-top of the earthworks that can affect sediment pH via the release of organic compounds. Ballidon sediment, nevertheless, remains mildly alkaline and porewater after equilibration with the limestone chatter will have a pH >8 although fresh rainwater infiltrating the earthworks may lower the pH temporarily (pH of rainwater is between 5.5 and 6.5)^28^. Glass dissolution often follows a U or V shaped profile with respect to pH^29–32^ with greater dissolution expected under acidic conditions (through greater inter-diffusion of H_3_O^+^ and ion exchange for alkali metals) and under alkaline conditions (through increased hydrolysis of the silicate network and greater silicon solubility). Notably there are exceptions with some glasses including medieval stained glass compositions (similar to Glass 2) showing low pH dependency at alkaline pH^33^.

**Table 4:** Chemical characterisation at the Ballidon site between 2019 and 2022

Parameter	Mid-mound	Base of mound
pH	7.8–8.1	8.1–8.2
Temperature	2.2–18.4 °C Average 9.2 °C	Not measured
Soil water content	0.04–0.33 m^3^/m^3^	Not measured
Redox (based on Fe(II) measurements during excavation)	No Fe(II) detected (June 2022)	No Fe(II) detected (June 2022)
Total Organic Carbon	3.27 ± 1.04	6.61 ± 1.54

**Fig. 4:**
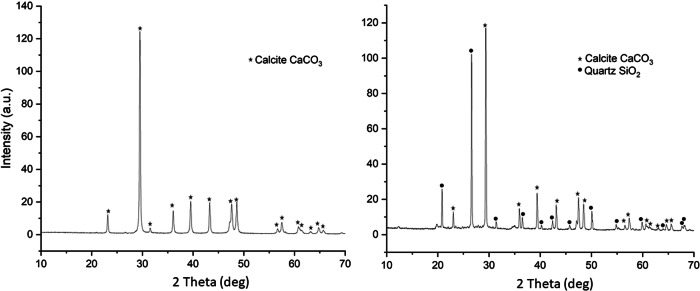
Mineralogy of the sediment at Ballidon. XRD trace of (left) the limestone chatter from the middle of the mound, and (right) limestone chatter and topsoil at the base of the mound.

**Fig. 5:**
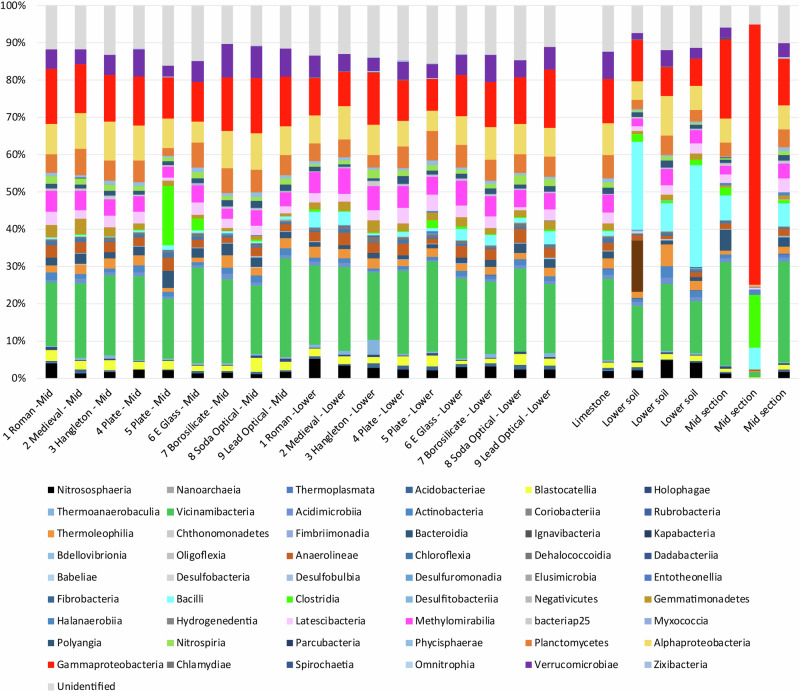
Microbial community analysis at the class level. 16S rRNA analysis for swabs taken from the surface of each glass sample, swabs taken from a limestone clast and from three samples of sediment form the middle (mid) and lower part of the earthwork.

Samples were analysed for total organic carbon as an indication of biological matter within sediment at both the mid-earthworks and at the base of the earthworks. The sediment mid-earthworks contained approximately half the TOC content of that at the base of the earthworks. The decrease is not linear but instead reflects the low organic matter content of the limestone chatter from which the earthwork is constructed. The sediment beneath is the original organic-rich forest topsoil (Fig. 4). The limestone chatter, in contrast, is course and fast draining with only a very thin layer of vegetation covering the top surface; likely the source of some of the TOC measured within the limestone. Analysis by XRD confirmed the presence of calcite (CaCO_3_) only, whilst XRD from the base also contained quartz (SiO_2_), and some minor peaks (likely clay phases) that could not be positively identified (Fig. 5). Chemical analysis by EPMA and SEM-EDS identified chemistries consistent with calcium carbonate (>90%), aluminosilicates (<10%) and minor iron oxide phases (<1%).

Porewater chemistry in the earthwork is expected to vary depending on rainfall and an estimate was made by centrifugation of sediment to obtain enough porewater to enable compositional analysis by ICP-OES (Table 5).

**Table 5:** Ballidon environment porewater in ppm measured by ICP-OES

Location	Element (ppm) and LOQ								
	Al	Ca	Fe	K	Mg	Na	P	Si	Zn
Limit of quantification	0.61	0.19	0.22	0.95	0.17	0.50	0.03	0.19	0.03
Farmer’s Field	< LOQ	84.70	< LOQ	35.43	7.715	8.17	0.45	7.38	< LOQ
Top of Earthwork	< LOQ	80.35	< LOQ	12.13	2.63	6.69	0.60	3.54	< LOQ
Earthwork surface	< LOQ	61.69	< LOQ	17.10	2.19	8.94	0.65	2.53	< LOQ
30 cm from Earthwork	< LOQ	77.73	< LOQ	9.98	2.61	5.49	0.44	6.07	< LOQ
20 cm depth	< LOQ	80.38	< LOQ	11.89	2.19	6.66	0.52	4.69	< LOQ
Burial depth	< LOQ	87.13	< LOQ	6.227	2.29	10.43	0.74	5.70	< LOQ
3 m from Earthwork	< LOQ	71.39	0.389	6.327	2.66	4.48	1.55	7.92	< LOQ

Only enough porewater was retrieved by centrifugation to run one sample per site and, therefore, triplicate analysis was not possible. Errors associated with ICP-OES are <5%

### Site microbiology

Microbial communities associated with the surface of the nine glass samples and the surrounding sediment were comprised > 50% of *Vicinamibacteria*, *Gammaproteobacteria, Alphaproteobacteria, Methylomirabilia, Planctomycetes* and *Verrucomicrobiae* classes typical of terrestrial soils (Fig. 5; Supplementary Tables 1 and 2). With the exception of the lead silicate glass, all samples were expected to be non-toxic to microorganisms. There appeared to be little variation between the bacterial species associated with the surface of each glass, including the lead silicate sample (Supplementary Tables 1 and 2). The microbial communities associated with the glass samples were most comparable to that associated with a swabbed piece of limestone, likely presenting similar conditions as regards pH, saturation and available nutrients.

Simpsons diversity index values for all were in excess of 0.99 (Supplementary Table 3). At the species level most were aerobes as might be expected from the porous, above-ground earthworks, however, there were several species identified at both the upper and lower levels that were capable of anaerobic respiration and the utilization of alternative electron acceptors such as Fe(III) and sulphate (Supplementary Tables 4–9). These included members of the genus *Geoalkalibacter, Geobacter, Acidibacter, Desulfitobacterium, Latescibacterota, Anaeromyxobacter, Desulfatitalea* and unidentified members of the *Desulfobacteraceae* family^34–39^. Although present, known iron and sulphate reducers made up < 2% of each community and were likely largely dormant whilst the earthworks remained aerobic. In addition to microbial species 18S, rRNA detected over 3000 species of Eukaryotic life including plants and fungal species, the former likely detritus from the topsoil and surrounding woodland (Supplementary Table 10–12).

### Sample characterisation

**Fig. 6:**
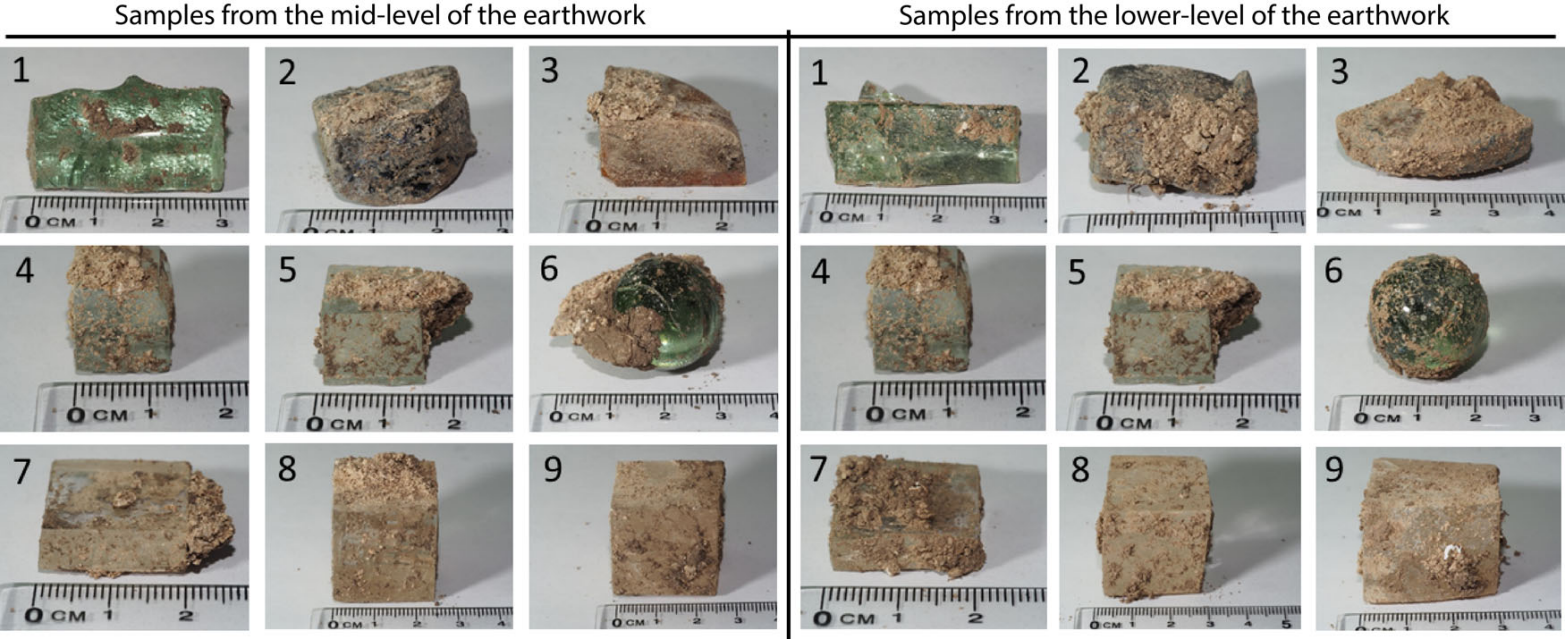
Glasses 1–9 removed from Ballidon. (left) Samples from the mid-level of the earthwork, and (right) samples from the lower-level of the earthwork.

**Fig. 7:**
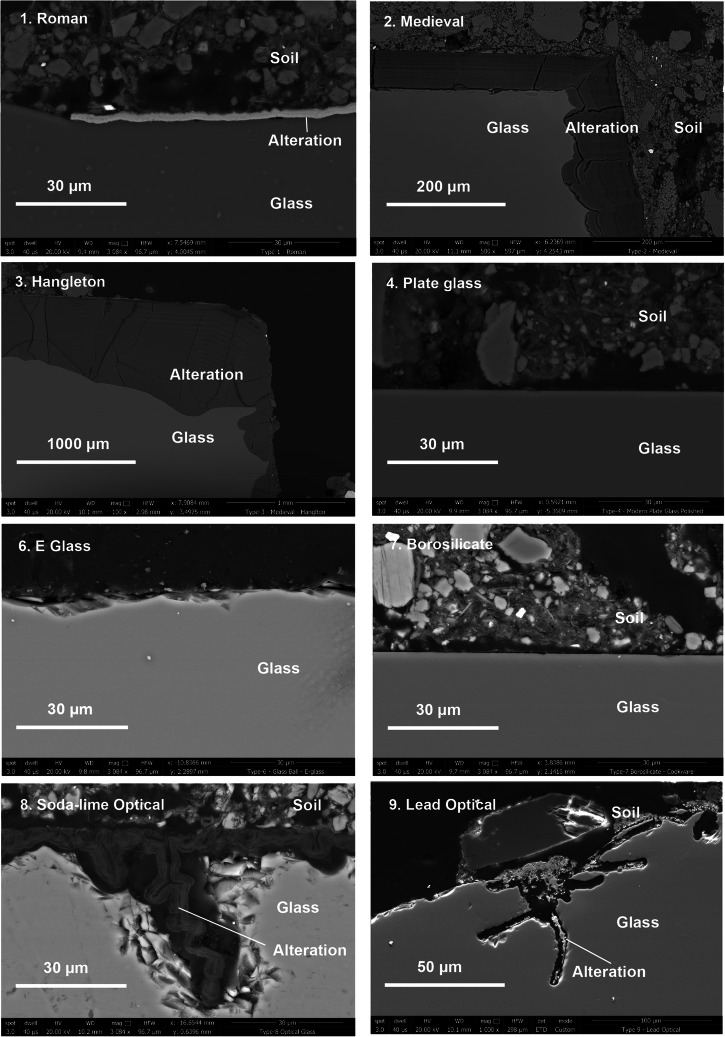
BSE-SEM images of alteration observed on the surface of Glasses 1–4 and 6–9 retrieved from the upper level of the Ballidon site. Sample 5 is omitted because it is of the same glass composition as Glass 4.

Samples removed from the mid-level and lower level of the earthwork were photographed prior to encapsulation in epoxy resin to preserve any alteration layers and attached sediment (Fig. 6). Unless otherwise stated, analysis presented in this manuscript has been conducted on samples from the mid-level of the earthwork. Historical records of the sample shapes and later SEM-EDS analysis were used to identify the samples (Tables 1 and 6). Alteration layers were apparent on glasses 2, 3, 8 and 9: the model medieval glass (Medieval), the Hangleton Linen Smoother (Hangleton), the soda-lime-silica optical glass (Soda Lime Optical) and the lead silicate optical glass (Lead Optical). Intermittent alteration was observed on Glass 1, the model Roman glass (Roman) (Fig. 7).

**Table 6:** Compositions of glasses buried at Ballidon in wt% as reported by Fletcher 1972 and converted to mol%

Glass																		
	1		2		3		4		5		6		7		8		9	
	wt%	mol%	wt%	mol%	wt%	mol%	wt%	mol%	wt%	mol%	wt%	mol%	wt%	mol%	wt%	mol%	wt%	mol%
SiO_2_	68.5	69.8	50.1	53.0	46.5	49.8	72.7	71.9	72.7	71.9	54.3	56.0	79.1	81.5	70.1	72.1	54.2	73.6
Al_2_O_3_	3.6	2.2	4.3	2.7	3.5	2.2	1.1	0.6	1.1	0.6	14.1	8.57	1.8	1.1	0.8	0.5	0	0
Fe_2_O_3_	0.49	0.2	0.95	0.38	1.2	0.48	0.09	0.03	0.09	0.03	0.42	0.1	0.03	0.01	0	0	0	0
P_2_O_5_	0	0	0	0	4.6	2.1	0	0	0	0	0	0	0	0	0	0	0	0
CaO	7.3	8.0	18.6	21.1	21.6	24.8	9.2	9.7	9.2	9.7	17.4	19.2	0.2	0.2	9.5	10.5	0	0
MgO	0.5	0.8	4.7	7.4	5.4	8.6	3.1	4.6	3.1	4.6	4.4	6.7	0	0	1	1.5	0	0
Na_2_O	18.2	18.0	2.6	2.6	0.7	0.7	13.3	12.7	13.3	12.7	0.25	0.25	4.2	4.2	10.6	10.6	8	10.5
K_2_O	1.3	0.8	16.7	11.2	16.4	11.2	0.5	0.3	0.5	0.3	0.35	0.23	0.3	0.2	5.1	3.4	4.1	3.5
PbO	0	0	0.1	0.03	0	0	0	0	0	0	0	0	0	0	0	0	33.6	12.2
MnO	0.34	0.2	0.63	0.46	0	0	0	0	0	0	0	0	0	0	0	0	0	0
CuO	0	0	0.08	0.06	0	0	0	0	0	0	0	0	0	0	0	0	0	0
CoO	0	0	1.03	0.87	0	0	0	0	0	0	0	0	0	0	0	0	0	0
B_2_O_3_	0	0	0	0	0	0	0	0	0	0	8.0	7.1	14.3	12.7	0.6	0.5	0	0
F	0	0	0	0	0	0	0	0	0	0	0.5	1.6	0	0	0	0	0	0
BaO	0	0	0	0	0	0	0	0	0	0	0	0	0	0	2	0.8	0	0
Sb_2_O_3_	0	0	0	0	0	0	0	0	0	0	0	0	0	0	0.3	0.07	0.05	0.01
As_2_O_3_	0	0	0	0	0	0	0	0	0	0	0	0	0	0	0.06	0.02	0.1	0.04

**Fig. 8:**
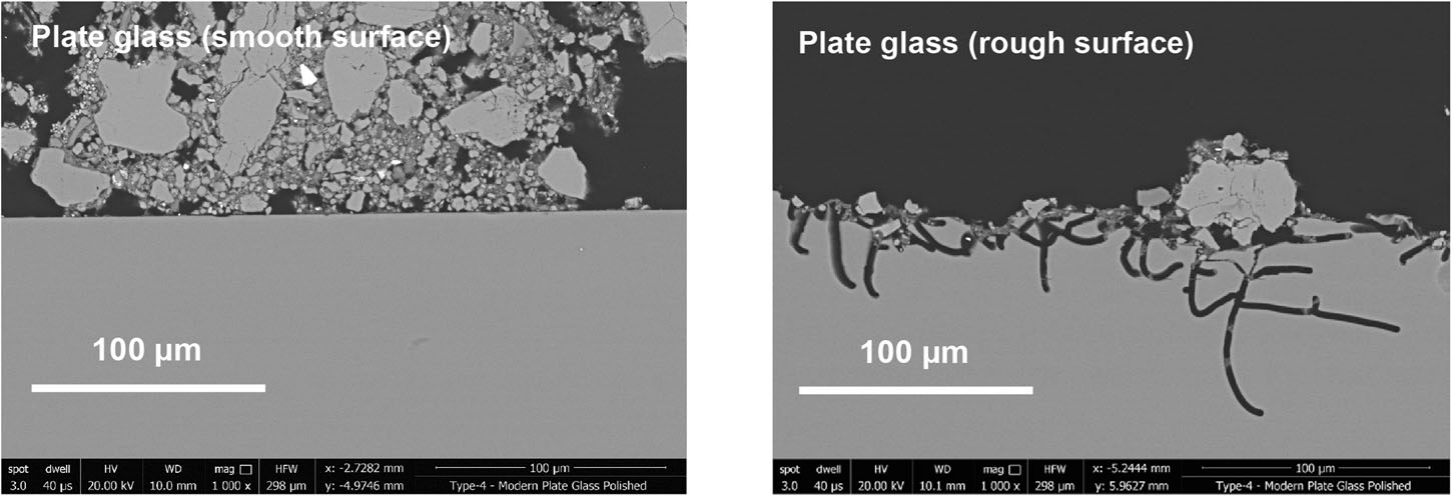
Narrow 3–5 µm diameter vermiform features on the surface of a rough surface on plate glass (Glass 5). Vermiform features were not observed smooth surfaces (polished and as-formed) of the Plate Glass.

Corrosion observed on Ballidon samples is summarised in Fig. 7 with additional images for each glass provided in Supplementary Figs. 10–20. Where alteration layers were present, they were often fractured. Fracturing is thought to have occurred in-situ as a result of dry periods rather than as the result of sample preparation. Glass 3 (Hangleton) showed the largest alteration layers followed by Glass 2 (Medieval), Glass 8 (Soda Lime Optical) and Glasses 1 and 9 (Roman and Lead Optical). Glasses 4, 5 and 7 showed no alteration layers or secondary mineral phase precipitation but all three glasses exhibited extensive vermiform features^40^ on at least one side. Vermiform features in these samples (discussed in depth in Mansfield et al.^40^) took the form of ~2–3 µm diameter channels typically 20–100 µm in length and were abundant on the rough-cut surfaces of Glass 4 and 5 (Plate Glass) (Fig. 8; Supplementary Figs. 14 and 15). Vermiform features of a similar dimension were also seen on the rough-cut faces of Glass 7 (borosilicate glass) (Supplementary Figs. 17 and 18). These features were free of obvious alteration layers, however, particles occasionally observed within the vermiform features could be either small particles from the sediment or precipitates from the glass (e.g. Supplementary Figs. 17 and 18). Much wider channels ~ 10–15 µm in diameter and 10–100 µm in length were observed on all faces of Glasses 8 and 9 (Soda Lime Optical and Lead Optical glasses) and these were lined with secondary alteration phases.

Although alteration features were comparable between samples removed from the mid-earthwork and the base of the earthwork, greater corrosion was observed on samples from the lower level (the earthwork/soil interface). The difference in extent of corrosion is most obvious on the least durable glass Glass 3 (Hangleton) where a corrosion layer of up to 1000 µm was observed surrounding the sample taken from the lower level, whilst the mid-earthwork showed variable alteration layer thickness between 50 and 1000 µm. This difference is likely due to the higher moisture content of the lower part of the earthwork adjacent to the soil, which, it must be presumed, took longer to dry out than the upper parts. Hence, the glasses at the lower level spent more time exposed to water.

### Alteration on Glass 1: Roman

**Fig. 9:**
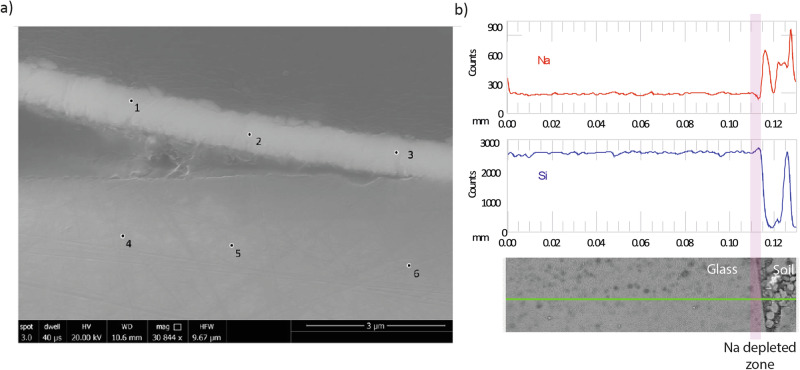
Analysis of glass 1. **a** SEM-SE image of alteration observed intermittently around the Roman glass (Glass 1) and locations of EDX point analysis (Table 7) and **b** line scan across the glass surface where the red line is Na relative sodium concentration, the blue line is the relative Si concentration, the green line shows the location of the line scan on an SEM-BSE image and the pink band shows the Na depleted area of the glass.

A continuous gel layer was absent from Glass 1, however, there were intermittent regions where a secondary precipitate was observed that was considerably enriched in Fe compared to the bulk glass (Fig. 9; Supplementary Fig. 10). Fe-rich alteration between 1 and 3 μm in thickness was observed and showed evidence of detachment that may explain why the layer was not observed around the whole of the sample (Supplementary Fig. 10; Table 7). This layer may be a precursor to Fe-rich phyllosilicates similar to those observed in other studies (e.g. refs. ^40–42^), however, the high Fe/Si ratio of the layer (between 1.5 and 3.3) more strongly supports the formation of amorphous Fe(III)-oxy-hydroxide within the silica gel as observed by Pèlegrin et al. on the surface of altered basalt^43,44^. As well as Fe, EDX analysis detected minor amounts of Al, Na, K, Al, Mn, Mg, P and Ca in the altered layer, all of which were also detected in the glass. In addition to visible alteration layers, EPMA analysis detected sodium depletion in the first 5 μm of glass agreeing with previous observations by McLoughlin et al.^3^.

**Table 7:** Chemical composition of pristine and altered glass determined by EDX from Glass 1 (Roman glass) in oxide wt% of elements detected assuming standard oxide state

Spot	Description	wt% SiO_2_	wt% Al_2_O_3_	wt% Na_2_O	wt% K_2_O	wt% MgO	wt% CaO	wt% P_2_O_5_	wt% MnO	wt% Fe_2_O_3_
1	Alteration layer	18.2 ± 1.8	1.7 ± 0.2	4.1 ± 0.4	0.3 ± 0.0	0.0 ± 0.0	1.4 ± 0.2	0.0 ± 0.0	0.4 ± 0.0	73.7 ± 7.4
2	Alteration layer	31.9 ± 3.1	1.7 ± 0.2	4.9 ± 0.5	0.5 ± 0.0	0.3 ± 0.0	2.8 ± 0.3	0.4 ± 0.0	0.5 ± 0.0	56.9 ± 5.7
3	Alteration layer	22.2 ± 2.2	1.2 ± 1.1	6.2 ± 0.6	0.4 ± 0.0	0.5 ± 0.1	1.8 ± 0.2	0.4 ± 0.0	0.3 ± 0.0	67.2 ± 6.7
4	Pristine glass	74.8 ± 7.4	3.5 ± 0.3	10.1 ± 1.0	1.6 ± 0.2	0.6 ± 0.1	8.1 ± 0.8	0.0 ± 0.0	0.2 ± 0.0	1.0 ± 0.1
5	Pristine glass	75.2 ± 6.6	3.5 ± 0.4	9.5 ± 0.9	1.5 ± 0.1	0.6 ± 0.1	8.7 ± 0.9	0.0 ± 0.0	0.3 ± 0.0	0.6 ± 0.5
6	Pristine glass	71.9 ± 7.4	3.2 ± 0.2	12.3 ± 1.6	1.5 ± 0.2	0.6 ± 0.1	8.2 ± 0.9	0.25 ± 0.0	0.4 ± 0.0	1.7 ± 0.4

### Alteration on Glass 2: medieval

**Fig. 10:**
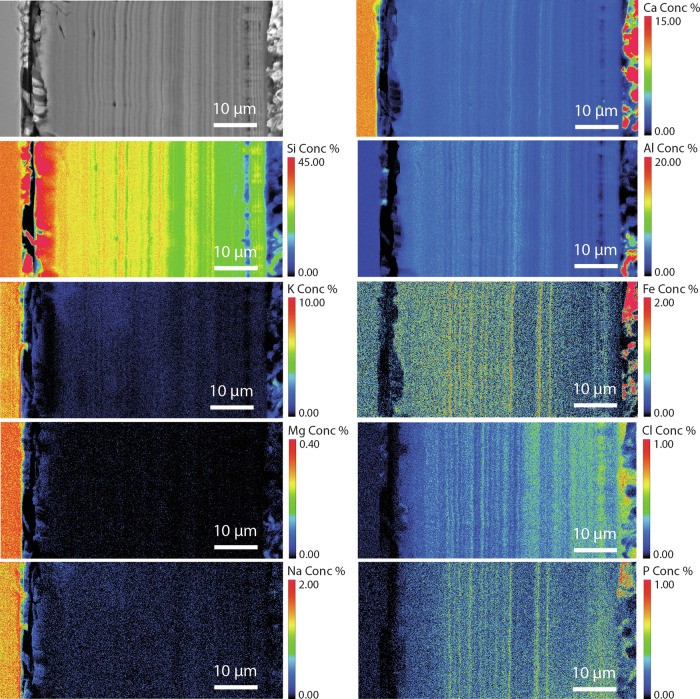
EPMA of the Glass 2. Banded alteration layers observed throughout the glass of medieval composition.

The medieval composition (Glass 2) had a thick alteration layer on all sides (up to 100 µm on the lower earthworks sample) (Fig. 7; Supplementary Fig. 11). This layer frequently exhibited ‘banding’, which was especially clear where the layer was thickest and in parts where soil adhered. On one face, to which there was little soil adhesion, the alteration layer was thinner (10–15 µm) and contained extensive vermiform features. It was unclear whether these differences were due to sample orientation/soil adhesion (that could have led to a variation in moisture), or to the difference in surface finish. The fine banding observed in the SEM images was further investigated using EPMA (Fig. 10).

Semi-quantitative chemical analysis by EMPA confirms that the banding corresponds to changing chemistry and potentially an accompanying change in density^45–48^ (Fig. 10; Supplementary Table 13; Supplementary Fig. 12). It can be seen from the mapped distribution of Si that there is a silica-rich, un-banded region approximately 7 µm in thickness next to the pristine glass. Closer towards the outer edge of the altered glass (presumed to be older) the alteration layer is made up of alternating regions of two distinct chemical environments: either containing the elements Si, Fe, P and Ca, or the elements Si and Al. Bands are of variable thickness typically less than 1 µm, although they appear less distinct closer to the glass and are indistinguishable in the silica-rich band closest to the pristine glass suggesting initial formation of a silica rich layer before evolution over time to a more complex chemistry. Although most elements are present in the glass, P and Ca concentration are notably low in the bulk glass meaning they have been augmented by Ca and P from the surrounding environment.

**Fig. 11:**
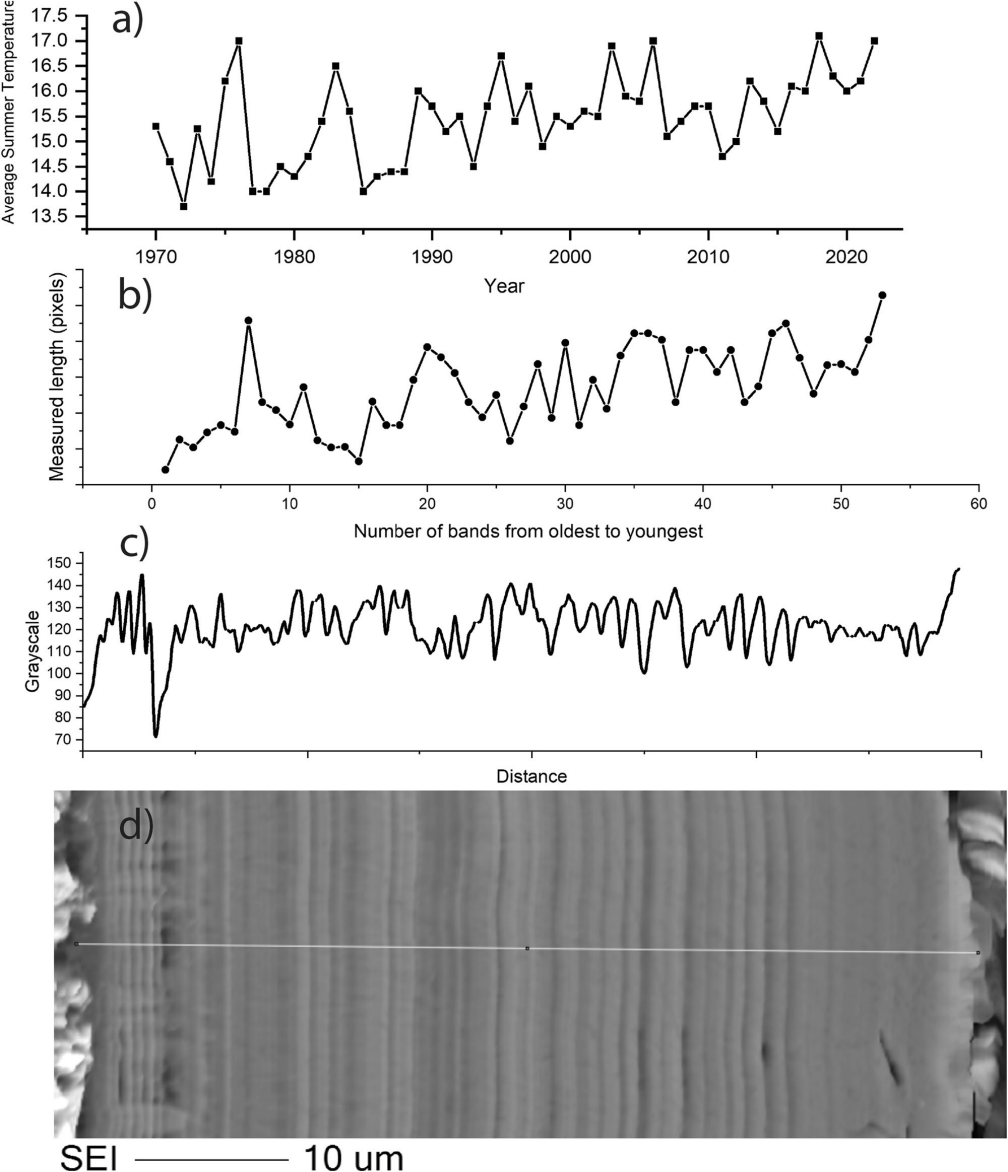
Analysis of banding on Glass 2. **A** average summer temperature^26^, **B** thickness of alteration layers from oldest to youngest measured in ImageJ, **C** grayscale profile across an SEM image of an unfractured alteration layer and **D** SEM image of the alteration layer observed on Glass 2 (Medieval).

The number of bands was counted on several images to ascertain if there is any correlation between banding and the number of years that the glass was exposed to the environment. This was done both by eye and using imageJ software to create a greyscale profile across the image (e.g. Fig. 11). Lines counted by eye numbered between 40–60 depending on the image resolution and judgement as to what constitutes a separate band. Using imageJ, variations in greyscale can be determined by peaks and troughs across the profile of a line scan drawn from one edge to another. On the highest resolution SEM image of the entire unfractured alteration layer, 50 cycles (peak and trough) of varying frequency (corresponding to thickness) were counted (Fig. 11). These were compared to climate data obtained and particularly to the average summer temperature that (considering the earthworks rarely dried out completely) was likely the dominant control on the alteration rate of the glass. Both the average summer temperature and the thickness of alteration layers increase over the 50-year timeframe.

### Glass 3: The Hangleton linen smoother

**Fig. 12:**
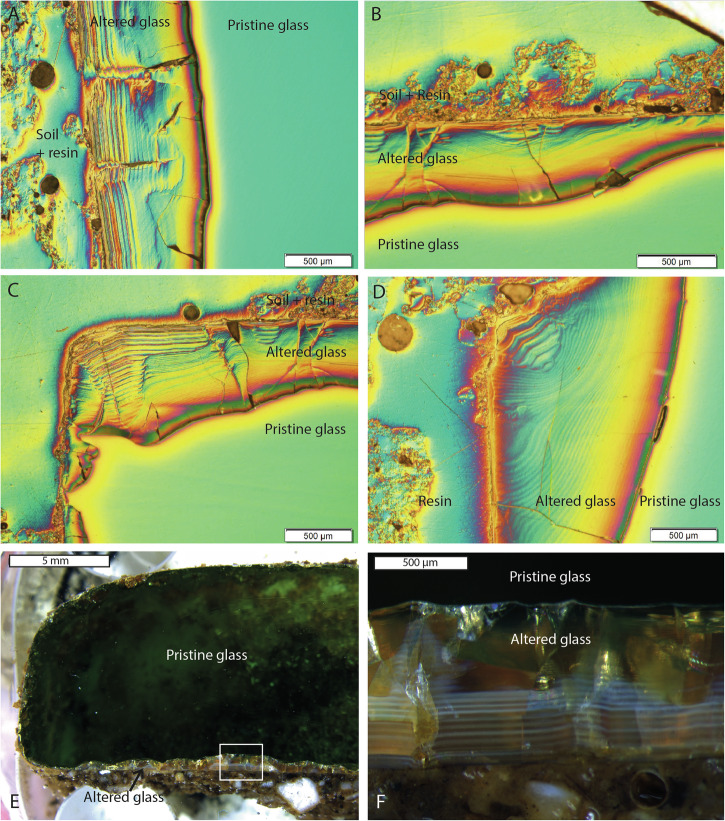
Analysis of Glass 3. Alteration layer banding on Glass 3 evident by, **A**–**D** differential interference contrast imaging and **E**, **F** visible light microscopy.

The Hangleton linen smoother composition (Glass 3) was the least durable glass and the alteration layer thickness varied greatly with some layers up to 1000 µm in thickness (Fig. 7; Fig. 12; Supplementary Fig. 13).

**Fig. 13:**
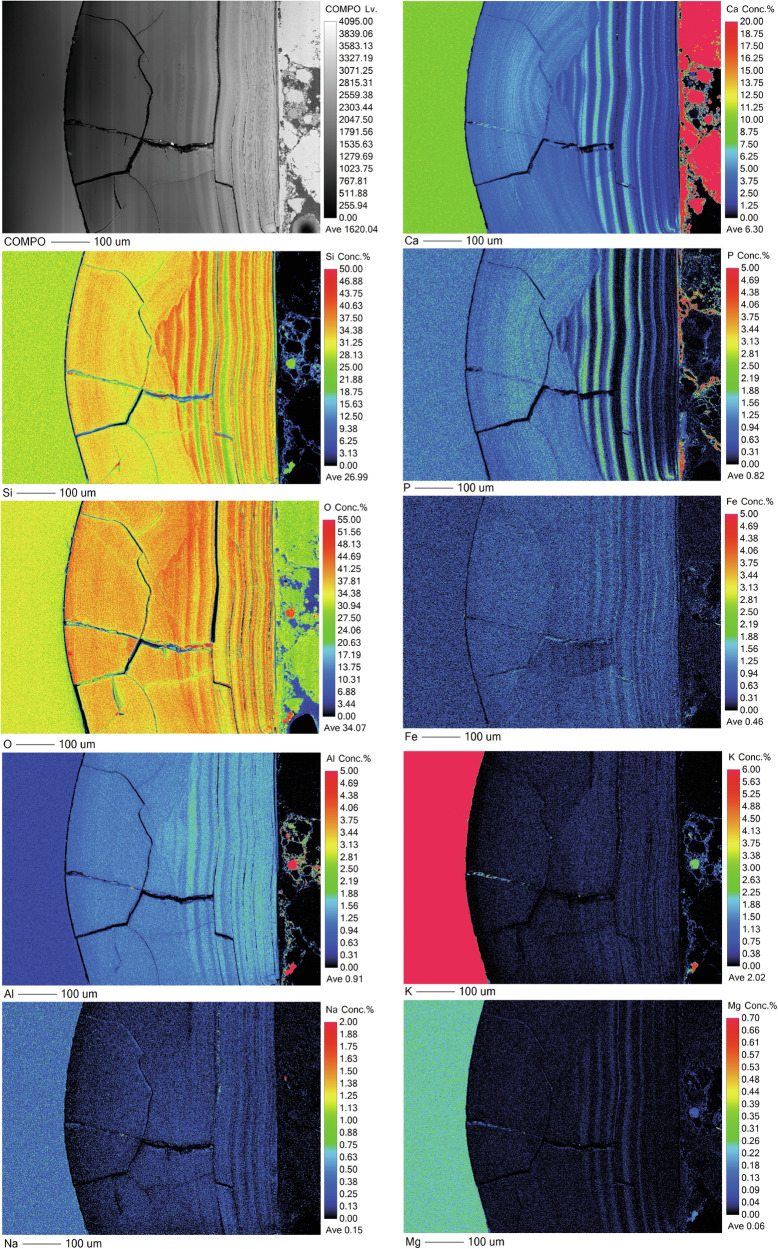
EMPA of major chemical components of the alteration layer from Glass 3.

**Fig. 14:**
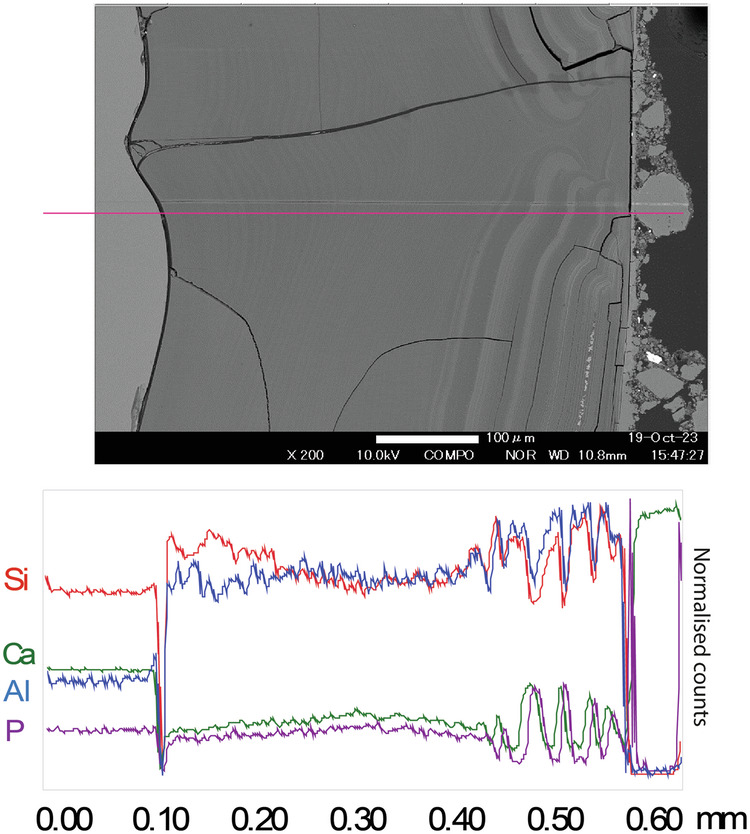
Line scan across the alteration layer of Glass 3, showing the normalised counts for key elements Si, Ca, Al and P.

**Fig. 15:**
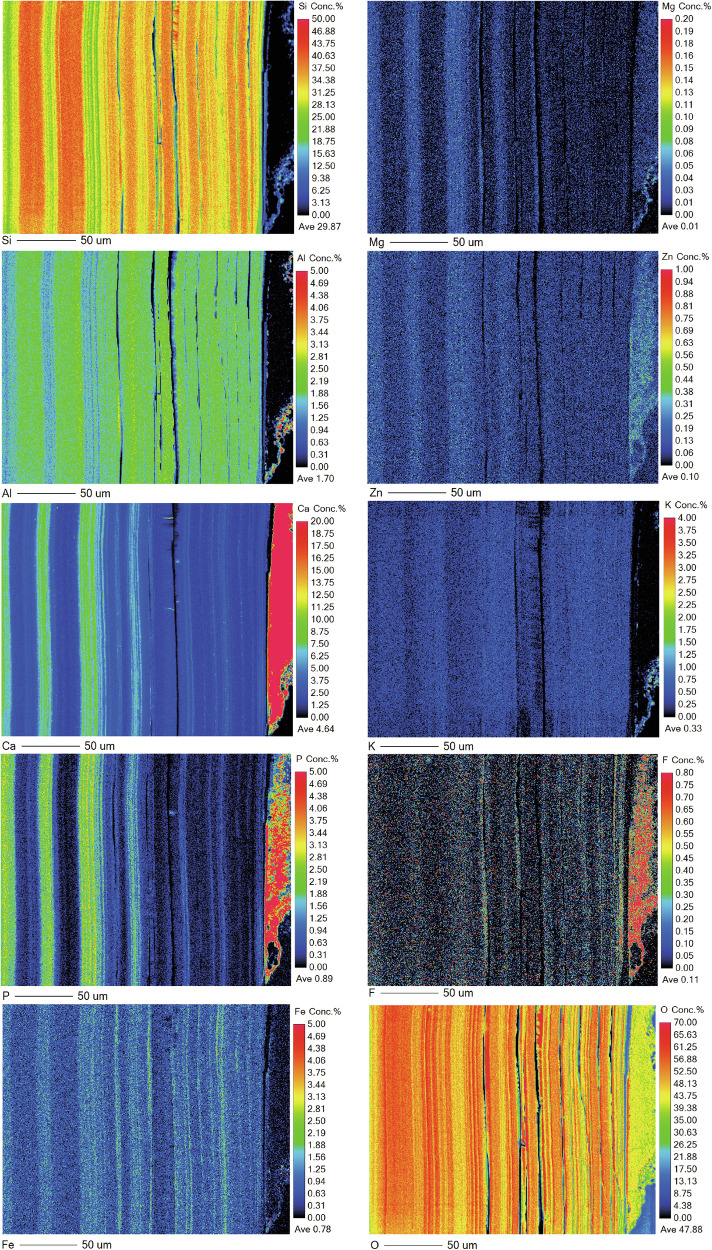
EPMA mapping of a thickly banded outer edge of the alteration layer on Glass 3. Pristine glass to left and alteration layer surface to the right.

**Fig. 16:**
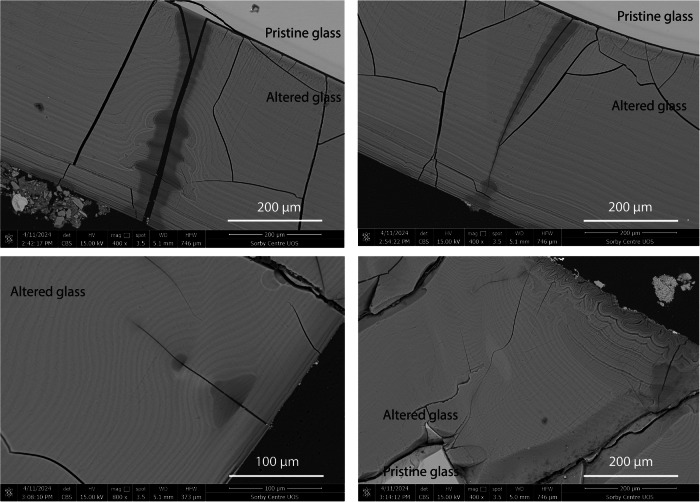
BSE-SEM images of alteration observed on the surface of Glass 3. Images show areas where cracks have apparently resulted in post-formation leaching and chemical re-ordering of the alteration layer.

As with the medieval glass, the alteration layers showed distinct banding but here on the scale of tens of microns (Fig. 12) that was discernible under light microscopy. EMPA analysis revealed to be chemically different regions following the same elemental groupings as observed in Glass 2; Si- and Al-rich layers alternating with layers rich in Si, Ca, P and some Fe (Figs. 13, 14 and 15). In some areas, the structure of the banding was particularly curious, for example, a region of thickly banded alteration appeared crosscut by a fine-banded (and presumably younger) region of alteration closer to the glass (Fig. 13). SEM imaging provided further evidence that chemical banding was not static and rather moved over time due to the ingress of fluid down newly formed cracks (Fig. 16). Higher resolution mapping shows additional correlation of Ca-, P- and Fe-rich zones with Mg and Zn as well as layers where Fe is concentrated alone (Fig. 15). In addition to banding, a white secondary phase was observed on the surface of the altered glass that comprised a Ca- and P-rich precipitate containing some F, likely a hydroxyapatite-like phase (Ca(PO_4_)_3_OH) (Fig. 15). With micron-scale resolution it appears that the Fe-, Ca-, P- rich zones within the layers contained significant Si however it could not be determined, with available analytical techniques, whether nano-scale crystalline Ca-P phases exist between silica layers or whether the layer is non-crystalline.

### Glass 8: soda lime optical glass

**Fig. 17:**
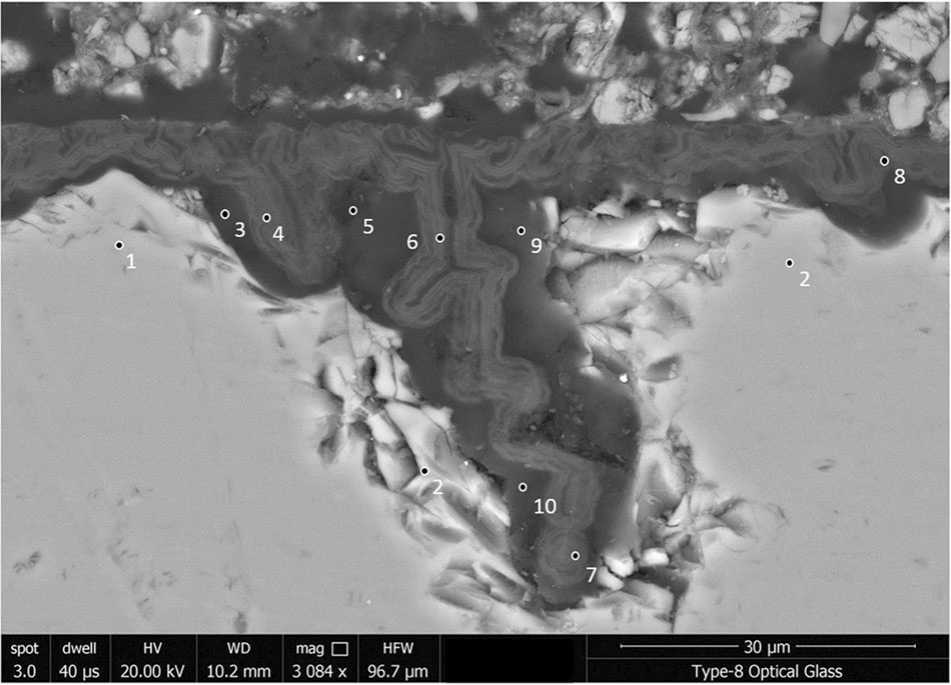
BSE-SEM image of the alteration layer on Glass 8 (Soda-Lime Optical glass).

The alteration observed on Glass 8, the Soda Lime Optical glass, was consistent and followed the surface contours including lining the relatively wide vermiform features present (Fig. 17). Here too alteration layers showed evidence of banding, however on too finer scale to be chemically resolved by EPMA. EDX analysis of the darker regions of the silica rich gel layer contained significant contribution from Cl, Ca, Al, K and Na, whilst the lighter regions were depleted with regard to K and Na. Neither Mg nor Ba were retained in the gel layer (Table 8).

**Table 8:** Semi-quantitative EDX analysis of selected spots in the pristine glass and altered regions of Glass 8 (soda-lime optical) (Fig. 17) in oxide wt% assuming standard oxide states

Spot	Description	SiO_2_	Al_2_O_3_	Na_2_O	K_2_O	MgO	CaO	BaO	Cl
1	Pristine glass	70.4 ± 6.5	1.0 ± 0.1	7.4 ± 1.3	5.8 ± 0.7	1.2 ± 0.10	11.6 ± 0.1	2.5 ± 0.1	–
2	Pristine glass	70.3 ± 6.5	1.1 ± 0.1	7.2 ± 1.3	5.9 ± 0.7	1.5 ± 0.1	11.8 ± 1.2	2.2 ± 0.1	–
3	Dark gel	66.0 ± 5.7	3.2 ± 0.3	1.3 ± 0.2	3.6 ± 0.4	–	9.7 ± 0.9	–	16.3 ± 2.4
5	Dark gel	50.1 ± 3.9	–	–	3.3 ± 0.3	–	7.6 ± 0.6	–	38.9 ± 5.2
9	Dark gel	55.0 ± 4.4	3.5 ± 0.3	4.3 ± 0.7	3.4 ± 0.4	–	7.1 ± 0.6	–	26.3 ± 3.6
10	Dark gel	65.6 ± 5.6	3.6 ± 0.4	2.4 ± 0.4	2.8 ± 0.3	–	8.2 ± 0.8	–	17.3 ± 2.5
4	Light gel	85.4 ± 8.2	5.3 ± 0.6	–	–	–	4.7 ± 0.5	–	4.5 ± 0.8
6	Light gel	84.6 ± 8.1	5.7 ± 0.6	–	–	–	4.6 ± 0.5	–	5.1 ± 0.8
7	Light gel	84.0 ± 8.0	4.9 ± 0.6	0.7 ± 0.1	1.1 ± 0.1	–	5.2 ± 0.5	–	4.1 ± 0.7
8	Light gel	83.0 ± 0.7	6.5 ± 0.7	–	–	–	4.8 ± 0.5	–	5.6 ± 0.9

### Glass 9: Lead optical glass

**Fig. 18:**
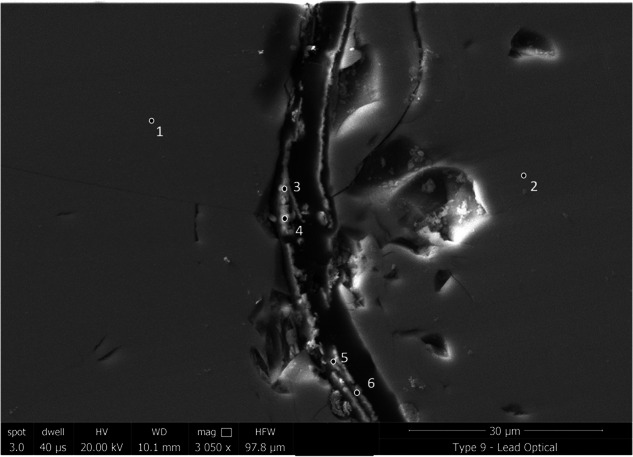
BSE-SEM image of the alteration layer on Glass 9 (Lead Optical glass).

Unlike Glass 8 (soda-lime-silica optical glass), where no elements were present in the layers that were not also detected in the pristine glass, alteration on Glass 9 (the lead optical glass) contained significant amounts of P and minor amounts of Ca that can only have come from the surrounding environment (Fig. 18; Supplementary Fig. 20). EDS analysis of secondary phases shows PbO (~70 wt%), CaO (4.5 wt%) and P_2_O_5_ (~12 wt%) (Table 9), which correspond to atom % ratios Pb (~30%), Ca (~15%) and P (~20%) consistent with a Ca substituted lead-apatite-type phase (e.g. Pb_5_(PO_4_)_3_OH). Due to the presence of other elements (e.g. Ca and Si) it is unlikely that stoichiometric apatite phases will form but rather the phase is an amorphous precursor. It is not known how the formation of lead-apatite-like secondary phases in this case might influence the ongoing dissolution of the glass compared to dissolution in their absence, but clearly the alteration layer chemistry of Glass 9 in this natural environment is different to that which would be determined under a laboratory setting and is thus likely an avenue for further research.

**Table 9:** Semi-quantitative EDX analysis of selected spots from Fig. 18 in the pristine glass and altered regions of the lead optical glass (Glass 9) in mol%

Spot	Description	SiO_2_	Na_2_O	K_2_O	P_2_O_5_	CaO	PbO	Cl
1	Pristine glass	52.1 ± 4.3	5.5 ± 1.4	4.3 ± 0.7	–	–	37.9 ± 1.3	–
2	Pristine glass	52.3 ± 4.3	5.5 ± 1.4	4.4 ± 0.7	–	–	37.7 ± 1.3	–
3	Precipitates	3.0 ± 0.5	2.5 ± 0.7	0.6 ± 0.1	12.0 ± 2.3	4.8 ± 1.8	74.0 ± 3.5	3.0 ± 0.9
4	Precipitates	3.6 ± 0.6	3.0 ± 1.0	0.6 ± 0.1	11.7 ± 2.2	4.4 ± 1.7	73.3 ± 3.4	3.1 ± 0.9
5	Precipitates	6.9 ± 1.1	3.5 ± 1.1	0.9 ± 0.2	11.1 ± 2.0	4.4 ± 1.6	69.8 ± 3.1	3.4 ± 1.0
6	Precipitates	4.9 ± 0.8	3.2 ± 1.1	0.9 ± 0.2	11.4 ± 2.1	4.7 ± 1.7	71.2 ± 3.2	3.5 ± 1.0

Note that standard oxide states may not be appropriate for all secondary alteration phases and so results are only semi-quantitative

### Laboratory dissolution tests

To compare the nine glasses according to their forward dissolution rates, independent of the influence of alteration layer formation or the surrounding soil, a Stirred Reactor Coupon Analysis (SRCA) test was performed (ASTM C1926^20^). Tests were conducted under two conditions; at a temperature of 10 °C and pH 8.2 (pH fixed using 0.1 M TRIS buffer) for 28 days and at a temperature of 90 °C in deionised water (pH 6.8) for 7 days. The former was intended to replicate pH and temperature conditions at the Ballidon site whilst the latter was intended to rank the glasses in order of their relative durability. During SRCA, glass coupons, polished on one side to a 1 µm diamond finish and half-masked with silicon, were exposed to a large volume of agitated solution (surface area to volume ratio approximately 0.05 m^−1^) for a given number of days in a purpose built reactor vessel. The amount of material dissolved was then calculated by measuring the ‘step height’, the height difference between the masked and unmasked portion of the coupon using Vertical Scanning Interferometry (VSI).

**Fig. 19:**
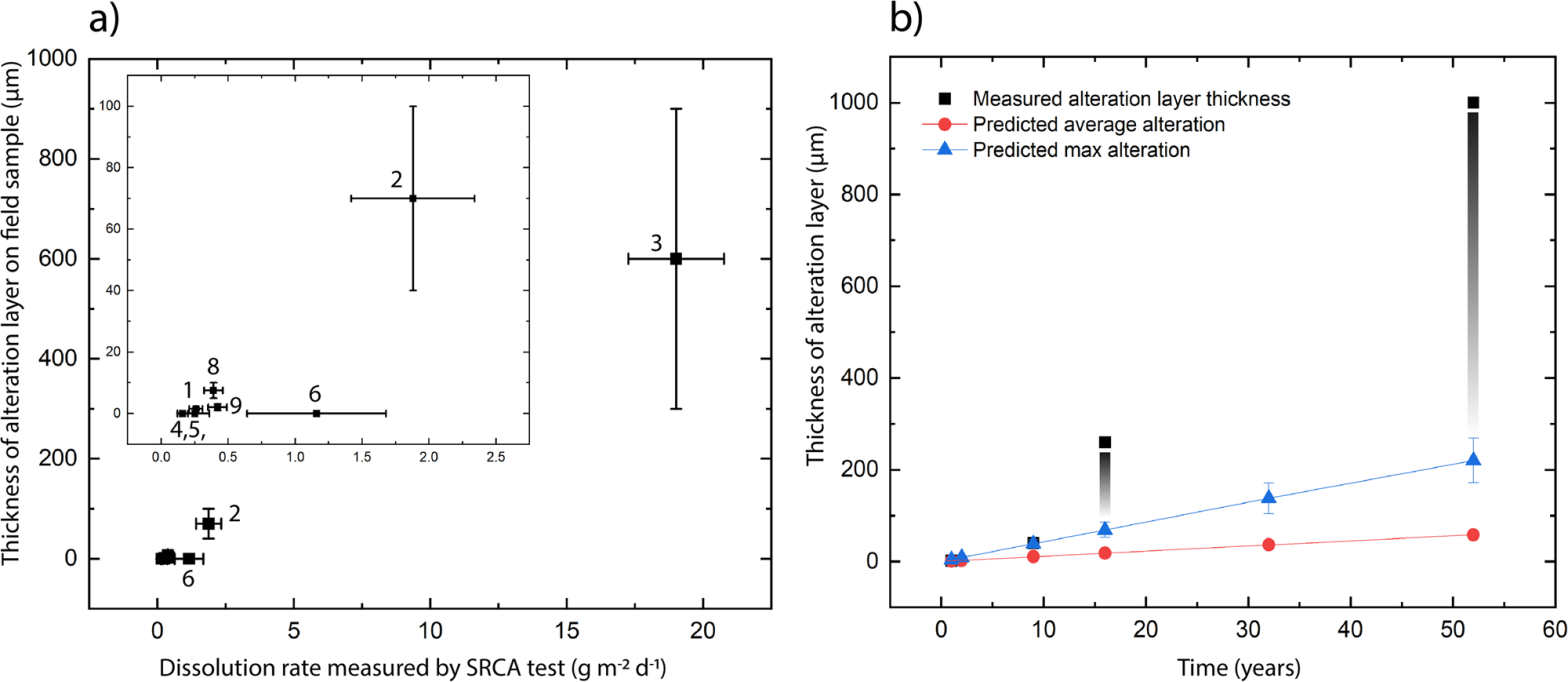
Comparison of laboratory testing with alteration layers observed on field samples. **a** Dissolution rates measured at 90 °C in deionised water by SRCA test plotted against alteration layer thickness measured on field samples and **b** thickness of alteration layers measured on Glass 3 (Hangleton) compared with alteration layer thickness predicted by average and maximum step height measured by SRCA test at 10 °C and pH 8.2 (pH maintained using TRIS buffer).

Due to extremely low corrosion rates at pH 8.2 and 10 °C, a change in height between the masked and unmasked part of the sample could only be measured on the fastest corroding glass, Glass 3 (Hangleton) (Table 10). Measurement of this height was further complicated by the uneven corrosion texture meaning that the new surface height of the sample, and therefore the volume of material lost, was difficult to determine (Supplementary Fig. 21). The average change in surface height and the maximum change in surface height were used to calculate an average surface retreat rate of 1.13 ± 0.03 µm y^−1^ ((8.3 ± 0.2) ×10^−3^ g m^−2^ d^−1^) and a maximum surface retreat rate of 4.30 ± 1.05 µm y^−1^ ((3.14 ± 0.76) ×10^−2^ g m^−2^ d^−1^). These gave a predicted alteration thickness of (average) 58.3 ± 1.33 µm and (max) 220.0 ± 48.6 µm, a factor of five lower than that alteration thicknesses observed in field samples. Figure 19b compares the rate of dissolution measured by SRCA with that observed on samples from Ballidon.

**Table 10:** Comparison of alteration layer thickness measured on the 52-year Ballidon samples with coupons of the same pristine glasses exposed to pH 8.2 solution at 10 °C for 28 days and deionised water at 90 °C for 7 days under dilute conditions (SRCA)

Glass	Density (ρ) (g cm^−3^)	Alteration layer thickness (µm)	Step height (h) after 28 d at 10 °C (µm)	Step height (h) after 7 d at 90 °C (µm)	Comparative rate g m^−2^ d^–1^ at 10 °C *Rate* = *[(h x* ρ*)/t]*	Comparative rate g m^−2^ d^–1^ at 90 °C *Rate* = *[(h x* ρ*)/t]*
1: Roman	2.504 ± 0.003	1–3	0	1.18 ± 0.2	N/A	0.42 ± 0.07
2: Medieval	2.659 ± 0.001	40–100	0	4.97 ± 1.2	N/A	1.88 ± 0.46
3: Hangleton linen smoother	2.662 ± 0.006	300–1000	Average: 0.087 ± 0.002 Max: 0.33 ± 0.080	50.0 ± 4.6	Average: 0.0083 ± 0.0002 Max: 0.0314 ± 0.0077	19.01 ± 1.75
4: Plate glass – polished	2.481 ± 0.003	0	0	0.45 ± 0.1	N/A	0.16 ± 0.04
5: Plate glass – as made	2.481 ± 0.003	0	0	0.71 ± 0.3	N/A	0.25 ± 0.11
6: E-glass	2.620 ± 0.027	0	0	3.1 ± 1.4	N/A	1.16 ± 0.52
7: Borosilicate cookware	–	0	0	No pristine glass available	N/A	–
8: Soda-lime-silica optical glass	2.539 ± 0.000	5–10	0	1.08 ± 0.2	N/A	0.39 ± 0.07
9: lead optical glass	3.195 ± 0.000	1–2	0	0.57 ± 0.1	N/A	0.26 ± 0.05

Comparative testing of glasses at 90 °C to determine the relative initial alteration rates of the nine glasses predicted the order of durability (from least durable to most durable) to be: Glass 3 (Hangleton) > Glass 2 (Medieval) > Glass 6 (E glass) > Glass 8 (Soda Lime Optical) > Glass 9 (Lead Optical) > Glass 1 (Roman) > Glass 4 & 5 (plate glass) ≥ Glass 7 (borosilicate cookware) (Fig. 19a). This was in agreement with the alteration layer thickness observed on field samples with the exception of Glass 6 (E-Glass marble) that showed no visible alteration layer on field samples but a dissolution rate similar to Glass 8 (Soda-lime Optical) in the SRCA.

## Discussion

### Agreement between field observations and laboratory tests

During 52 years’ burial, site monitoring confirmed that the nine glass samples had experienced variable saturation, fluctuating temperatures, and mildly alkaline conditions. The pH had reduced from an original pH of ~9.7 to a pH of ~8.2 likely through leaching by constant rainwater infiltration and the influence of biological processes (e.g. decaying plant matter and respiring microorganisms). Glass corrosion at the site can still be considered to be in the alkaline regime but the pH drop means that, for some glasses, the Ballidon earthwork is a less corrosive environment today than in 1970 (not all glasses show a V or U shaped response to changes in pH). Diverse microbial communities were present on the surface of all samples but community composition was comparable to that observed on the surrounding limestone implying that glass chemistry did not stimulate or select for particular bacteria.

Using alteration layer thickness as an approximation for extent of alteration, the relative durability of the nine glasses was comparable in both field and laboratory experiments with the one exception of Glass 6 (E-glass marble) (Fig. 19a). Glasses 4 and 5 (Plate Glass) and Glass 7 (Borosilicate) showed no visible surface alteration only some localised attack. The high chemical durability of these glasses was unsurprising as both have high SiO_2_ concentrations 72.0 mol % (Plate Glass) and 81.5% (Borosilicate) and relatively low alkali concentration of 13.0 mol % and 4.5 mol % respectively (Table 6). The only other glass to show no visible surface alteration in the field was Glass 6 (E-glass) despite having a lower SiO_2_ content at 56.0 mol % and the third fastest dissolution rate in laboratory testing. As the only spherical ‘marble’ shaped sample in the suite of glasses (the others being rectangular monoliths), we hypothesise that its increased aqueous durability compared to a flat ‘coupon’ of the same glass (used in laboratory testing) may be due to the difference in surface finish. The marble surface was heated or ‘flame polished’ to achieve its round shape and annealed (it did not fracture upon cutting). In contrast, the glass coupon used in the SRCA test was a slice cut from the marble, a process that likely introduced surface defects not present in the original surface. Dussossoy et al. showed that initial dissolution rates measured in the first 7 days were lowest for the flame polished sample when compared to samples prepared by polishing to various grades up to 1 µm and confirmed that superficial microcracks were introduced by cutting and polishing that were rare on flame-polished samples^49^. In addition, there may potentially be a difference in surface chemistry caused by intense heating. The removal of volatile elements (e.g. Na and K) from the glass surface during flame polishing, has the potential to change the surface chemistry and increase network connectivity locally^50^. Finally, the difference in pH between tests performed in deionised water (pH ~6.0 at 90 °C) and the alkaline porewater at Ballidon (pH >8) may be responsible and Glass 6 (rich in Ca and Al) may be more susceptible to dissolution under mildly acidic conditions. It is interesting to note that some iridescence was observed on Glass 6 buried at Wareham after 5 years^13^ where samples were altered under acidic conditions (pH <5) and where interdiffusion/ion exchange will have been the dominant dissolution mechanism.

Leaching of exchangeable alkali elements from glass surfaces was not observable by SEM-EDX, however, depletion of Na was observed in the outer 5 µm of Glass 1 (Roman) (Fig. 9b) when analysed by EPMA. Mcloughlin et al.^3^ reported a Na depleted zone of ~2 µm (1 year), ~6 µm (nine years) and up to >10 µm (32 years) and changes in infrared reflectance spectra that suggested structural changes to the Si in the outer 2–5 µm in the 32-year sample. It is not known if samples are directly comparable as Mcloughlin et al did not report if the sample measured was from the upper or the lower section of the earthwork^3^. Several authors, including Ojovan et al.^29^ and Strachan et al.^51^ have highlighted the importance of ion-exchange under low-temperature conditions and propose it as the dominant mechanism for elemental release from durable glasses over long time periods. Initial observations from the Ballidon site appear to support this and future work will employ higher resolution chemical analysis to assess surface chemistry of Glasses 1, 4, 5, 6 and 7 on the nanometre scale to determine the extent of leaching.

Alteration layers were observed on Glass 1 (Roman), Glass 2 (Medieval), Glass 3 (Hangleton), Glass 8 (Soda Lime Optical) and 9 (Lead Optical) with thicknesses of 1–3 µm, up to 50 µm, up to 1000 µm, 5–10 µm, and 1–2 µm respectively. The order of durability agreed with that predicted by short term laboratory tests (Table 7) with Glasses 1, 8 and 9 showing high durability reflecting their high SiO_2_ content: Glass 1 (69.8 mol %), Glass 8 (72.2 mol%) and Glass 9 (73.6 mol %) and Glasses 2 and 3 showing the thickest alteration layers reflecting their lower SiO_2_ contents of 53.0 mol % and 49.8 mol% respectively.

Although the formation of an alteration layers gives a method by which to track glass dissolution it cannot be assumed that the volume of the alteration layer is equal to that of the dissolved glass, especially in this near surface environment where drying causes separation between lamellae and a change in density. It is also possible, in this open system, that elements from the surrounding porewater can be sequestered and contribute to secondary mineral formation increasing the alteration layer thickness.

For Glass 3, a rate of alteration layer growth of approximately 3–4 µm per year was calculated for the first 9 years^3^ whilst a rate of 5–20 µm per year was calculated from the 52 year samples. It is possible that the dissolution rate of this glass has increased over the burial period (for example due to changing pH, temperature or saturation), however, another more probable explanation is that the alteration layers have expanded over time due to a combination of layer separation, inclusion of external elements and mineral precipitation. This observation was supported by comparison of field alteration with laboratory tests, conducted under ultra-dilute conditions at a pH of 8.2 and temperature of 10 °C representative of the Ballidon site. These tests measured the initial alteration rate; the rate of dissolution assuming a solution undersaturated with regard to Si and a non-protective alteration layer. Assuming a 1:1 ratio between glass dissolved and layer formed, laboratory tests predicted alteration layer 5 times smaller than those observed in the field. Accurate measurement of a step height was hampered by non-uniform dissolution, however, even using the maximum step-height from each line-scan the estimated initial dissolution rate of 4.3 ± 1.1 µm y^−1^ (220 ± 49 µm alteration after 52 years) is not sufficient to account for the alteration layers of up to 1 mm that were observed on field samples. There may be several explanations for this difference. As discussed, alteration layers may have a lower density to the glass and may separate over time when subject to wetting and drying cycles. Secondly, sequestration of elements from the environment (including Si, Ca and P) may cause an increase in layer volume and/or precipitation of secondary minerals between layers. Thirdly, as glass corrosion has a non-linear relationship with temperature, conducting dissolution tests at the average temperature of 10 °C will not have accounted for faster corrosion during the summer months where temperatures were upwards of 15 °C. Overall, laboratory and field experiments suggest the initial rate was maintained throughout the 52-year time-period with the alteration layer offering no significant protection either due to extensive fracturing or high porosity. Earlier studies suggested that the alteration layer on Glass 2 was protective^3^ but this study demonstrates that it has grown in thickness from ~20 µm after 30 years to ~50 µm after 52 years (an average of 1 micron a year).

### Alteration chemistry and morphology

Two types of banding can be observed in alteration layers; banding in the silica rich zones, likely corresponding to changes in the structure of the Si particles, and chemical banding caused by the regular precipitation of layers rich in Ca and P (and sometimes Fe). The banding in the silica rich zones is apparent on glasses 2, 3 and 8. It is tempting to equate the formation of lamellae to cyclical climate events in the manner of tree rings, despite similar features being observed occasionally in laboratory systems e.g. refs. ^30,47, 52^. At a near surface site like Ballidon the ‘tree ring’ theory has some merit as yearly variation in both temperature and rainfall (leading to variation in porewater composition and pH) may be the driver behind the changes in silica precipitation that lead to visible banding. Where an entire un-fractured alteration layer is visible (e.g. on Glass 2), the number of bands (at the resolution available in this study) appears to correspond approximately to the number of years the sample was buried (e.g. Fig. 11). Schalm et al.^47^ demonstrates formation of 1 µm lamellae at a temperature of 22 °C under laboratory conditions and proposes a mechanism: pH cycling in the local/interface solution leading to supersaturation and precipitation reactions that occurs only in the mildly alkaline pH regime (similar to that at the Balldion site). We suggest that the two hypothesis for lamellae formation are not mutually exclusive and that changes in pH at the glass-alteration layer interface caused by seasonal changes in temperature/rainfall could cause banding in a similar way. For example, faster dissolution during wet and warm periods could lead to an increase in pH in the interfacial fluid affecting polymerisation of silica released from the glass and leading to changes in the structure/packing of the silica particles e.g. refs. ^45–47^. Although multiple variables increase complexity, the general trend of alteration layer thickness across the layer appears to correlate with increasing summer temperatures. Whilst not wishing to make a definite conclusion or link alteration rate with a single factor it seems plausible that banding associated with silica could be linked to yearly climate variation. Analysis is hampered by the fact that layers have become separated over time and that post dissolution layer evolution obscures earlier patterns, particularly in the least durable glass (Glass 3).

The second type of banding - chemical banding – was defined by separation of the alteration layer into Si and Al rich zones and Ca, Fe and P rich zones. These bands are much less regular in thickness and often appear to have formed after the alteration layer or moved over time (e.g. Figs. 12 and 15). Banding associated with a change in chemistry has been observed on other glasses altered in natural environments^4,53–55^ and some laboratory systems^30,47,52^. Gel layer composition can be affected both by elements released from the glass and by those from the surrounding porewater, especially where porewater can infiltrate down cracks without the need for diffusion through an in-tact alteration layer and spread laterally between separated alteration lamellae. The crosscutting patterns and bending of banding around cracks (Figs. 12, 13 and 15) provide evidence that layers are continually evolving in response to new influx of fluid (e.g. through a new crack or channel). Lombardo et al.^54^ observed similar crosscutting patterns associated with Ca and P rich alteration lamellae/bands in medieval type glass and also attributed it to later ingress of fluid via cracks. Visually, the chemical bands somewhat resemble the natural phenomenon of Lisegang rings, structures formed by fluid diffusing through a porous medium and precipitation of mineral products at distinct horizons as saturation is reached^56^. Stephanich observed alternating layers comprising silica gel and crystalline calcium phosphate phases on phosphorus-containing glasses and hypothesised that ‘over-concentrations’ in the porosity between silica gel layers preclude the precipitation of a secondary phases. They supposed that differences in the leaching kinetics and rates of diffusion of elements within the alteration layer caused elements to ‘catch-up’ with each other and accumulate leading to super-saturation and the precipitation of secondary phases^54^. At Ballidon, in addition to diffusion through the gel layer it is also possible that porewater is flowing down cracks and then laterally into high porosity layers leading to super saturation and precipitation. Interestingly, in Backhouse et al., where banding was observed under laboratory conditions, bands rich in Zr (from the glass) formed in solution saturated with Ca(OH)_2_ and could have represented chemical banding formed by a similar process to those formed at Ballidon^30^.

Analysis of alteration layer chemistry also highlights the contribution of elements, notably Ca and P, from the environment. Phosphorus was observed to react with Pb released from Glass 9 (Lead Optical) to form a phase most closely resembling lead-apatite. In other samples P co-located with Ca, even where no Ca is present in the glass. As the most abundant element in porewater at Ballidon, Ca, was found to be present in all alteration layers regardless of glass composition.

Finally, localised corrosion was observed on most samples but with different morphologies depending on the durability of the glass. Vermiform features – long thin alteration channels- have been observed previously on glasses altered both fully saturated and atmospheric environments both in the field and laboratory e.g. refs. ^30,33,40, 57–60^. Samples from Ballidon provide convincing evidence that vermiform features formed preferentially on rougher surfaces lending weight to the theory that they originate from surface defects (e.g. pits or microcracking of a surface during cutting and polishing). Vermiform features were frequent and narrow on glasses with a high durability and a rough surface finish (those glasses with a flame polished or ‘as made’ surface showed few vermiform features). For example, the rougher surface on Glass 8, resulting from cutting, likely accounts for the prevalence of vermiform features observed when compared to the infrequent vermiform features observed on the Roman glass (that had an ‘as formed’ surface finish) despite the two glasses having similar alteration rates in laboratory experiments. It seems at first contrary that vermiform features were observed to be most prevalent on the most durable glasses, with the highest silica content, that otherwise did not form alteration layers. Relics of these features were observed on less durable glasses (e.g. Glass 3) having been quickly widened and obscured by more uniform corrosion. At Ballidon, the less durable the glass, the wider the vermiform features: 1–2 µm (Glasses 7, 4 and 5), 5 µm (Glass 9), 5–10 µm (Glass 8) and 10–100 µm (Glasses 2 and 3). In the most durable glasses, vermiform features are deep but narrow, implying that dissolution is occurring primarily at the tip of the channel. One hypothesis is that fluid, or vapour, collects at the tip becoming hyper-alkaline as ion-exchange occurs and this increases the solubility of silica locally within the feature (ref. ^61^ and references therein). It is also possible that crack propagation in these glass is accelerated by the presence of water or water vapour and this persists longer in more durable glasses where the cracks are not rounded off and pacified by alteration. The silica content of porewater within the earthwork was relatively low compared to the surrounding soil due to the predominantly limestone geology (Table 5) and undersaturated with respect to silica. No secondary phases were observed inside the vermiform features, meaning that the oligomerisation rate (the rate at which silicic acid nucleates into colloids) for silica gel remained low in these systems^61,62^. Although by no means uniformly orientated, there does appear to be some directional preference to vermiform features on some faces. Some appear remarkably straight, whilst some curl in a uniform direction (e.g. Fig. 8). Because sample orientation was not recorded at the time of excavation, it was not possible to determine if gravity plays a part in the formation of these features, although it is likely that they follow the direction of microcracks/defects in the first instance and in three dimensions may resemble typical concoidal glass fractures. Future sample retrievals from this or other sites should ensure that sample orientation is recorded.

Future analysis of the Ballidon glasses will discuss the alteration observed on UK, US and Russian radioactive waste glass compositions and what the findings of the Ballidon study mean in the context of radionuclide release rates from vitrified radioactive wastes. In addition, inspired by this study, further experiments will endeavour to perform systematic laboratory studies at variable temperature and pH in order to model corrosion at Ballidon with a view to validating predictive models for glass dissolution.

## Methods

### Excavation of glasses and site characterisation

On 27^th^ June 2022, the topsoil was removed from section G of the earthwork at Ballidon and the area staked out using wooden pegs. A trowel was used to remove first soil and then limestone chatter until the level of the first samples. Samples of sediment were taken from each level (mid-mound and the base of the mound) for analysis by X-ray diffraction (XRD). XRD data was collected on a Bruker D2 Phaser X-ray diffractometer with a Lynxeye position sensitive detector and Ni filter. Data was collected using a Cu-Kα source (λ = 1.5418), within the range 5–80°, step size of 0.02 and count time of ~2 s per step with 10 degrees of rotation. Phases were identified by comparison to known standards in the Crystallographic Open Database. Total organic carbon (TOC) was determined by Sievers M9 TOC analyser. The soil pH was measured in-situ by equilibration of 25 g sediment with 40 ml deionised water to obtain a 2:1 water to soil ratio and left for 1 h before measuring with a calibrated handheld pH probe (Oakton Handheld Meter with Oakton pH Electrode). The presence of Fe(II) in sediments was measured in-situ using portable UV-vis spectroscopy (Lovibond Photometer MD 600); 0.5 g of sediment was added to 10 ml 0.5 N HCl for one hour followed by analysis using the ferrozine assay as described in Viollier et al.^63^.

### Climate monitoring

Prior to excavation the temperature, soil water content, and rainfall at the Ballidon site were monitored at intervals of 4 h for 12 months (November 2020 to October 2021) using a HOBO weather station. Soil water content was measured using a HOBO EC-5 Soil Moisture Smart Sensor (reported detection range 0–0.550 m³/m³ and reported accuracy ±0.031 m³/m³ (±3.1%)). Temperature was measured using a HOBO 12-bit Temp Smart Sensor (reported accuracy ±0.25 °C from –40° to 0 °C, and ±0.20 °C from 0 °C to 70 °C). Water content and temperature probes were inserted ~15 cm into the earthworks whilst rainfall was monitored using a catchment system mounted to one of the posts that marked the earthworks. Weather observations collected from nearby weather stations can be viewed as gridded estimates of daily and monthly areal rainfall for the United Kingdom from the NERC EDS Environmental Information Data Centre and the Met Office archives^24–26^.

### Microbiology at the site

Bacterial and fungal species present at the site were assessed by 16S and 18S polymerase chain reaction (PCR) ribosomal ribonucleic acid (rRNA) gene analysis. 16S and 18S PCR rRNA analysis was performed on raw sediment samples from the base of the earthworks and the middle of the earthworks as well as on swabs taken from the surface of each glass immediately after they were removed. Swabbing was carried out using a sterile cotton bud dipped in filter-sterilized buffer solution as described in Plymale et al.^64^.

Deoxyribonucleic acid (DNA) was extracted from solid samples and swabs using a Dneasy PowerSoil Pro Kit (Qiagen, Manchester, U.K). Sequencing of PCR amplicons of 16S and 18S rRNA genes was conducted with the Illumina MiSeq platform (Illumina, San Diego, CA, USA) targeting the V4 hyper variable region (forward primer, 515 F, 5′-GTGYCAGCMGCCGCGGTAA-3′; reverse primer, 806R, 5′-GGACTACHVGGGTWTCTAAT-3′) for 2 × 250-bp paired-end sequencing (Illumina)^65,66^. PCR amplification was performed using Roche FastStart High Fidelity PCR System (Roche Diagnostics Ltd, Burgess Hill, UK) in 50 μl reactions under the following conditions: initial denaturation at 95 °C for 2 min, followed by 37 cycles of 95 °C for 30 s, 55 °C for 30 s, 72 °C for 1 min, and a final extension step of 3 min at 72 °C. The PCR products were purified and normalised to ~20 ng each using the SequalPrep Normalization Kit (Fisher Scientific, Loughborough, UK). The PCR amplicons from all samples were pooled in equimolar ratios. The run was performed using a 4.5 pM sample library spiked with 4.5 pM PhiX to a final concentration of 12% following the method of Schloss and Kozich^67^.

For QIIME2 analysis, sequences were imported into QIIME2 q2cli v2021.04^68^. The sequences were trimmed with cutadapt, visually inspected with demux, and denoised with DADA2^69^ to remove PhiX contamination, trim reads, correct errors, merge read pairs and remove PCR chimeras. Representative amplicon sequence variant (ASV) sequences and their abundances were extracted by feature-table^70^. QIIME2 plugins were executed with DADA2 quality settings “- -p-trunc-len-f” of 230 and “- -p-trunc-len-r” of 220. Taxonomical assignment was obtained with the *q2-feature classifier* plugin^71^ using the classify-sklearn naïve Bayes taxonomy classifier against the Silva v138 99% reference sequence database^72^. Contaminant sequences identified in extraction and PCR controls were manually removed.

### Characterisation of glass alteration

After removal from the earthwork, glass samples were preserved in Epothin-2 low viscosity epoxy resin (Buehler), cross sectioned using a slow saw (Buehler Isomet 1000 with diamond blade) and polished to a 1 µm finish with oil based diamond suspension. Samples were either gold or carbon coated for SEM imaging (in secondary electron and backscattered detection mode) and energy dispersive x-ray analysis (EDX) on an FEI Quanta 250 equipped with an Oxford Instruments EDX detector at the Dalton Cumbrian Facility or the Inspect F at The University of Sheffield. Selected samples were then cleaned and carbon coated for electron microprobe analysis (EPMA; JEOL JXA 8530F PLUS, Royce Institute, Sheffield) to quantify the chemical composition of altered glass layers. EPMA maps, line scans and spot analysis were taken at 10 keV, 50 nA, with a dwell time of 70.00 ms at intervals of 0.20 µm. Image analysis was conducted using ImageJ (open-source) that was used to plot changes in grayscale across SEM images of alteration layers to more accurately identify banding and measure banding thickness.

### Stirred reactor coupon analysis (SRCA)

A set of comparable pristine and unaltered samples, which had not been buried at Ballidon was also available for investigation. These samples had been kept in ambient conditions in plastic bags (it is presumed that they had been kept like this since 1970 as they were received in this state by one of the current authors (RJH) in 2002). Sections from these pristine samples were used to measure the intrinsic dissolution rate for the nine Ballidon glasses and the dissolution rate of the glasses at pH 8.2 (held by 0.1 N TRIS buffer adjusted with nitric acid) and 10 °C representative of average conditions at the Ballidon site. The SRCA procedure is described in detail in the ASTM standard C1926^19^. Briefly, samples were cut, polished to a 1 µm surface finish, and a silicon mask was applied to part of each sample before they were exposed to solution in a stirred reactor for seven days under ultra-dilute conditions. The step height between the masked and unmasked part of the sample was measured by vertical scanning interferometry (VSI) (Contour Elite). Step height was calculated by measuring the difference in surface height over an area at least 1 mm either side of the step using white light and at a magnification of ×20 (Supplementary Figs 21 and 22). Recorded step heights are the average of at least five measurements at different points across the sample. The dissolution rate in g m^−2^ d^–1^ at 90 °C was calculated using the equation *Rate* = *[(h x* ρ*)/t]* where h is the step height (m), ρ is the density (kg/m^3^) and *t* is time in days. Density was measured by Archimedes balance.

## Supplementary information

Supplemental Material

## Data Availability

The datasets used and/or analysed during the current study available from the corresponding author on reasonable request. The raw 16S sequencing data obtained in this project were deposited to NCBI SRA (Sequence Read Archive; http://www.ncbi.nlm.nih.gov/sra/) under the project accession number: PRJNA1102989.
