# Supplementary material for: Insights into long term glass corrosion mechanisms from the Ballidon experiment
Source: Npj Mater Degrad. 2025 Mar 17;9(1):27. doi: 10.1038/s41529-025-00571-0 (PMC11913731; doi:10.1038/s41529-025-00571-0)

Supplementary Information

Insights into long term glass corrosion mechanisms from the Ballidon project.

Thorpe, C.L.,*[1] Fisher, A.J.,[2] Manifold, G.,[1] Creasey-Gray, S.,[1] Jackson, C.M.,[3] Stone, B.,[3] Corkhill, C.L.,[4] Boothman, C.,[5] Lloyd, J.R.[5] and Hand, R.J.[1]

[1] School of Chemical, Materials, and Biological Engineering, University of Sheffield, Sheffield, UK.
[2] Dalton Cumbrian Facility, University of Manchester, Westlakes Science Park, Cumbria, UK.
[3] School of History, Philosophy and Digital Humanities, University of Sheffield, Sheffield, UK.
[4] School of Earth Sciences, University of Bristol, Bristol, UK.
[5] Williamson Research Centre, University of Manchester, Manchester, UK.

Figure	Page	Figure	Table	Page	Table
Figure 1	2	Rainfall data for the Ballidon area 1970 – 1977 summarised from publically available Met office data.	Table 1	24	Results by class of 16S rRNA analysis on glass samples from the mid-earthworks level, swabbed limestone clast and finer sediment particles.
Figure 2	3	Rainfall data for the Ballidon area 1978 – 1985 summarised from publically available Met office data.	Table 2	25	Results by class of 16S rRNA analysis on glass samples from the lower-earthworks level, swabbed limestone clast and finer sediment particles.
Figure 3	4	Rainfall data for the Ballidon area 1986 – 1993 summarised from publically available Met office data.	Table 3	26	Simpsons index and inverse Simpsons index as a measure of microbial community diversity.
Figure 4	5	Rainfall data for the Ballidon area 1994 – 2001 summarised from publically available Met office data.	Table 4	27	Results by species of 16S rRNA analysis on glass samples from the mid-earthworks level listing those known to be capable of Fe(III) reduction.
Figure 5	6	Rainfall data for the Ballidon area 2002 – 2009 summarised from publically available Met office data.	Table 5	28	Results by species of 16S rRNA analysis on glass samples from the lower-earthworks level listing those known to be capable of Fe(III) reduction.
Figure 6	7	Rainfall data for the Ballidon area 2010 – 2017 summarised from publically available Met office data.	Table 6	29	Results by species of 16S rRNA analysis on sediment and limestone samples from the mid- and lower-earthworks level listing those known to be capable of Fe(III) reduction.
Figure 7	8	Rainfall data for the Ballidon area 2018 – 2022 summarised from publically available Met office data.	Table 7	30	Results by species of 16S rRNA analysis on glass samples from the mid-earthworks level listing those known to be capable of sulphate reduction.
Figure 8	9	Average soil moisture for the Ballidon area	Table 8	31	Results by species of 16S rRNA analysis on glass samples from the lower-earthworks level listing those known to be capable of sulphate reduction.
Figure 9	10	Average summer temperature for the UK	Table 9	32	Results by species of 16S rRNA analysis on sediment and limestone samples from the mid- and lower earthworks level listing those known to be capable of sulphate reduction.
Figure 10	11	SEM-Backscatter detection images from Glass 1 (Roman).	Table 10	33-34	Results by order of 18S rRNA analysis on glass samples from the mid-earthworks level.
Figure 11	12	SEM-Backscatter detection images from Glass 2 (Medieval).	Table 11	35-36	Results by order of 18S rRNA analysis on glass samples from the lower-earthworks level.
Figure 12	13	Line scan across the alteration layer of Glass 2 showing the variation for key elements (measured in counts).	Table 12	37-38	Results by order of 18S rRNA analysis on sediment samples from the limestone, mid-earthworks level and lower earthworks
Figure 13	14	SEM-Backscatter detection images from Glass 3 (Hangleton).	Table 13	39	Atom % of elements measured by EPMA at points 1-14 indicated on the image below. Note that these data are semi-quantitive due to loss of Na, K during analysis. Results are given to 3 dp.
Figure 14	15	SEM-Backscatter detection images from Glass 4 (Plate glass).			
Figure 15	16	SEM-Backscatter detection images from Glass 5 (Plate glass).			
Figure 16	17	SEM-Backscatter detection images from Glass 6 (E-Glass marble).			
Figure 17	18	SEM-Backscatter detection images from Glass 7 (Borosilicate).			
Figure 18	19	SEM-Secondary electron image from Glass 7 (Borosilicate) and EDX of spot analysis.			
Figure 19	20	SEM-Backscatter detection images from Glass 8 (Soda Lime Optical).			
Figure 20	21	SEM-Backscatter detection images from Glass 9 (Lead Optical).			
Figure 21	22	Step height for Glass 3, Hangleton Linen Smoother, exposed to SRCA testing at pH 8.2, 10 ^{o}C for 28 days.			
Figure 22	23	measurement of step height by vertical scanning interferometry; average sample height are taken either side of the step (X profile).			

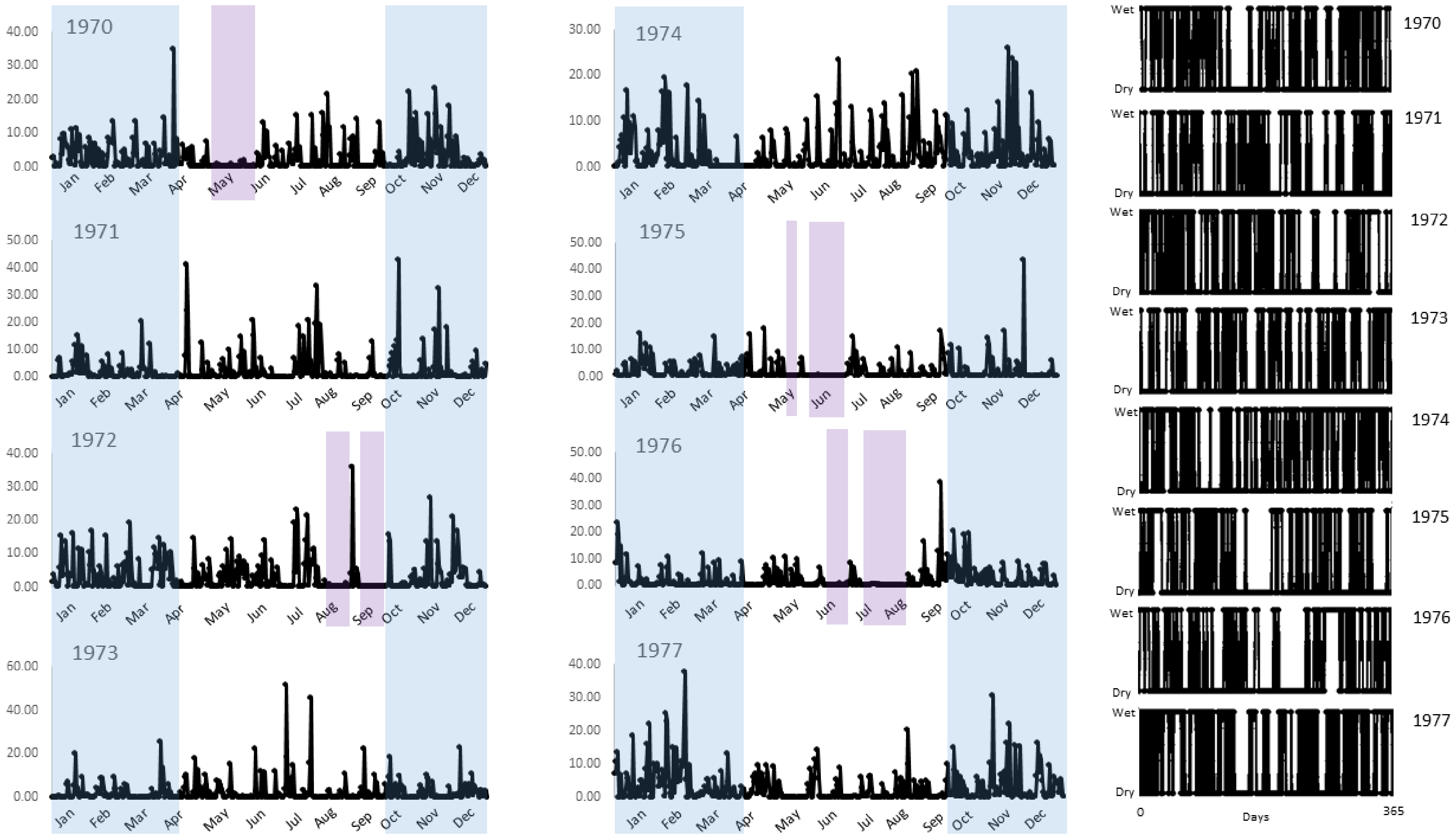

Supplementary Figure 1: Rainfall data for the Ballidon area 1970 – 1977 summarised from publically available Met office data from the Middleton Hillside (weather station 6.5 km from Ballidon). Data highlighted blue = winter months where the earthworks is continually wet and highlighted purple are extended dry periods in the summer where the earthworks may have dried out.

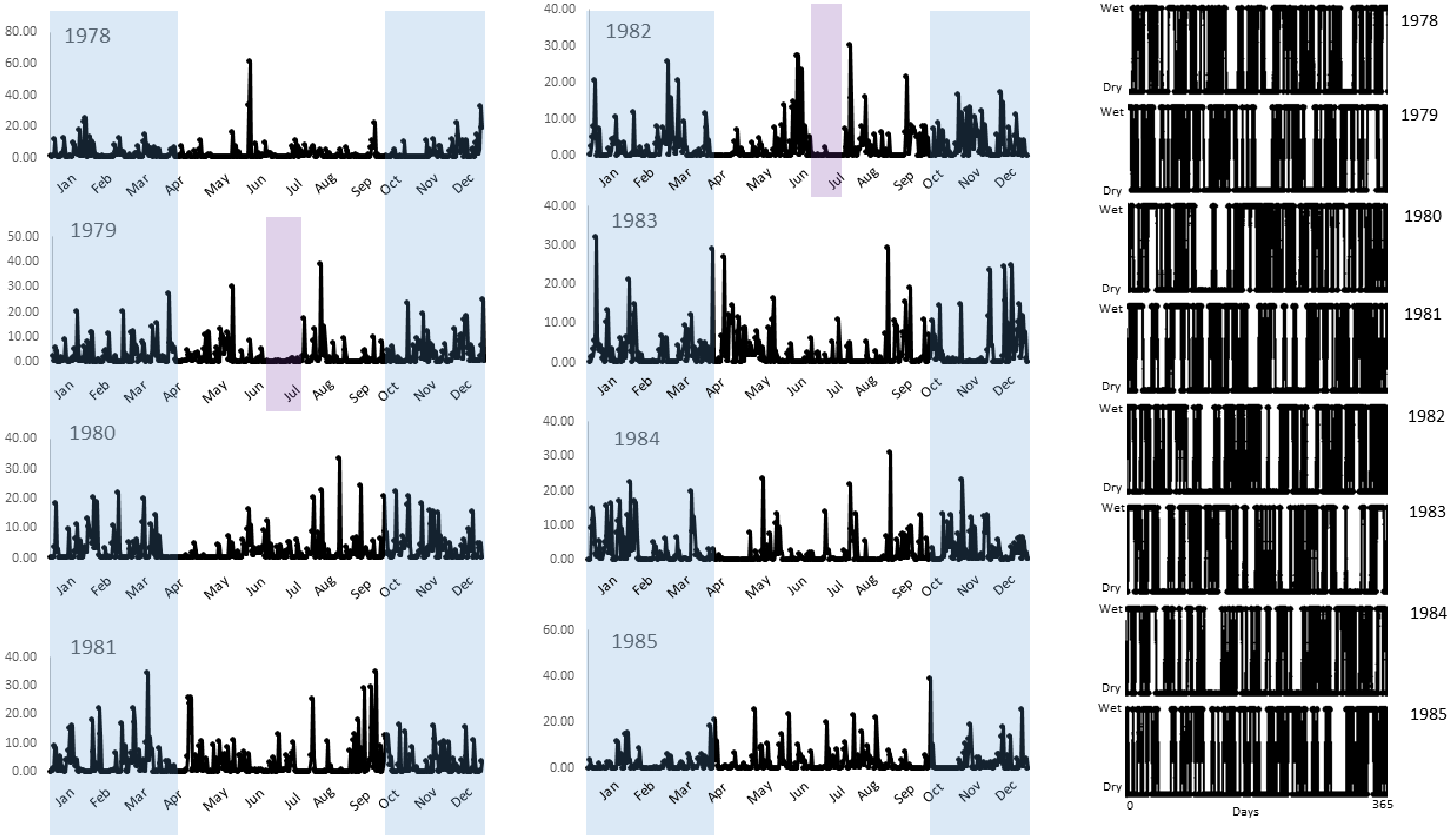

Supplementary Figure 2: Rainfall data for the Ballidon area 1978 – 1985 summarised from publically available Met office data from the Middleton Hillside (weather station 6.5 km from Ballidon). Data highlighted blue = winter months where the earthworks is continually wet and highlighted purple are extended dry periods in the summer where the earthworks may have dried out.

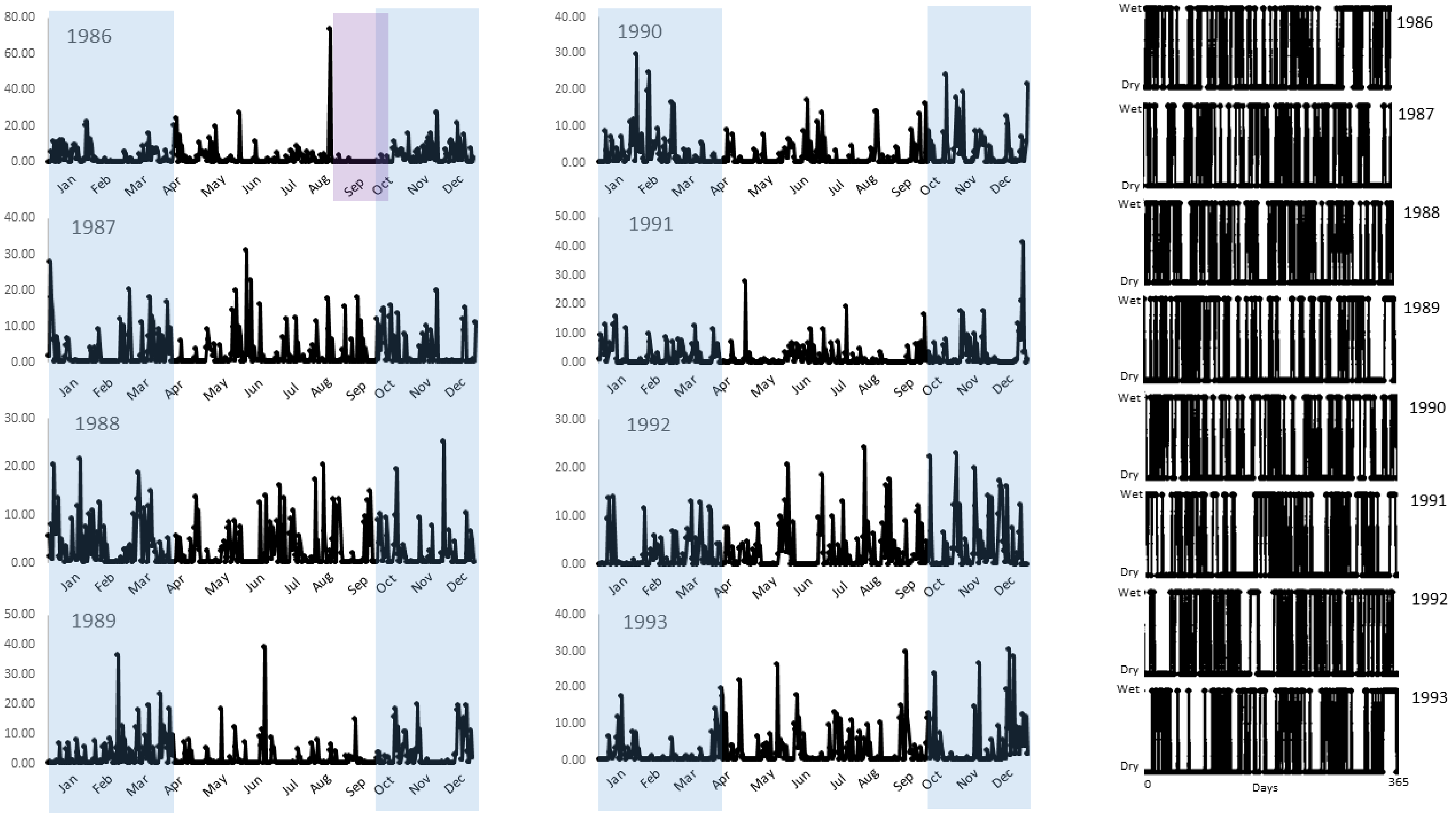

Supplementary Figure 3: Rainfall data for the Ballidon area 1986 – 1993 summarised from publically available Met office data from the Middleton Hillside (weather station 6.5 km from Ballidon). Data highlighted blue = winter months where the earthworks is continually wet and highlighted purple are extended dry periods in the summer where the earthworks may have dried out.

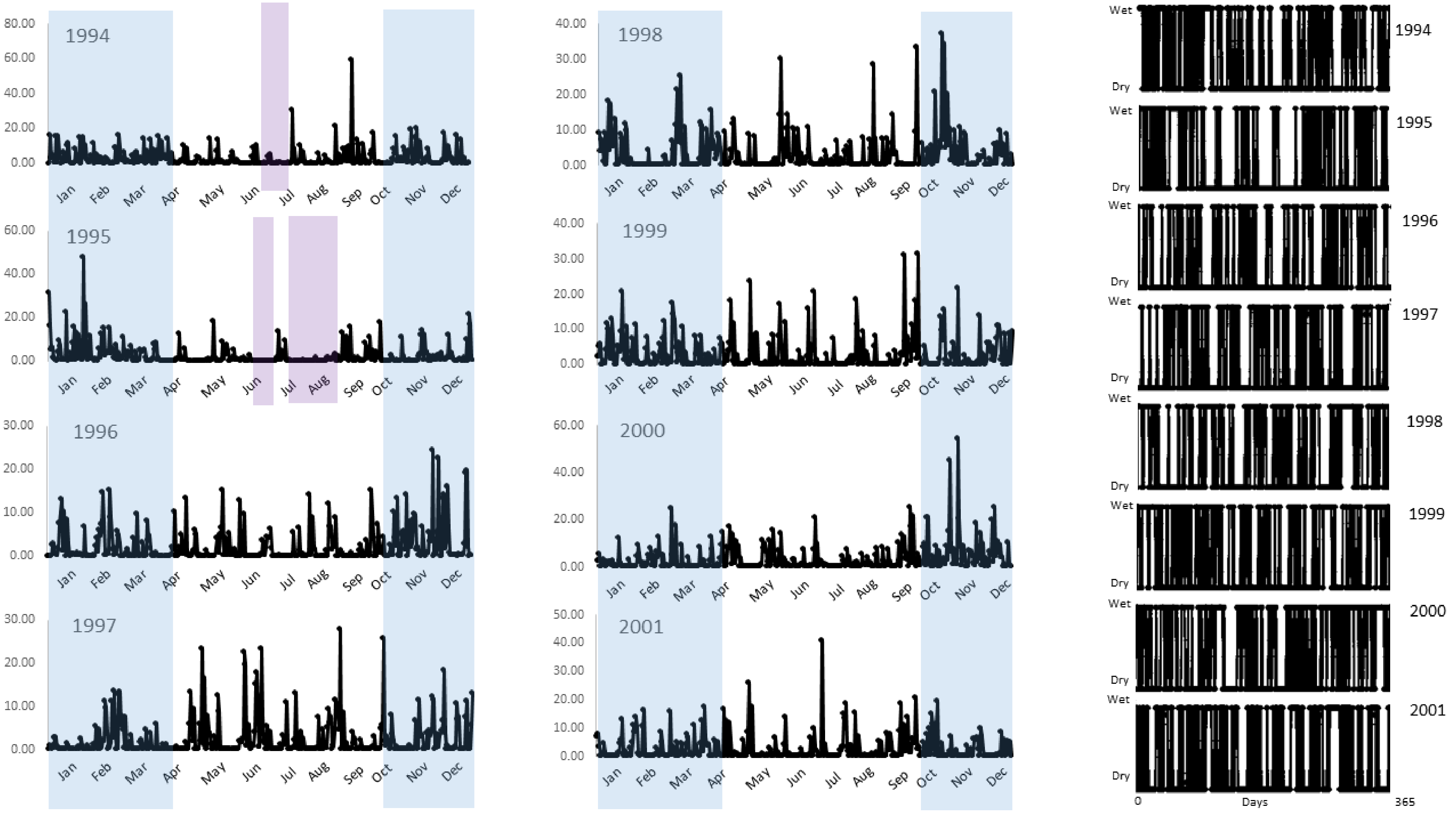

Supplementary Figure 4: Rainfall data for the Ballidon area 1994 – 2001 summarised from publically available Met office data from the Middleton Hillside (weather station 6.5 km from Ballidon). Data highlighted blue = winter months where the earthworks is continually wet and highlighted purple are extended dry periods in the summer where the earthworks may have dried out.

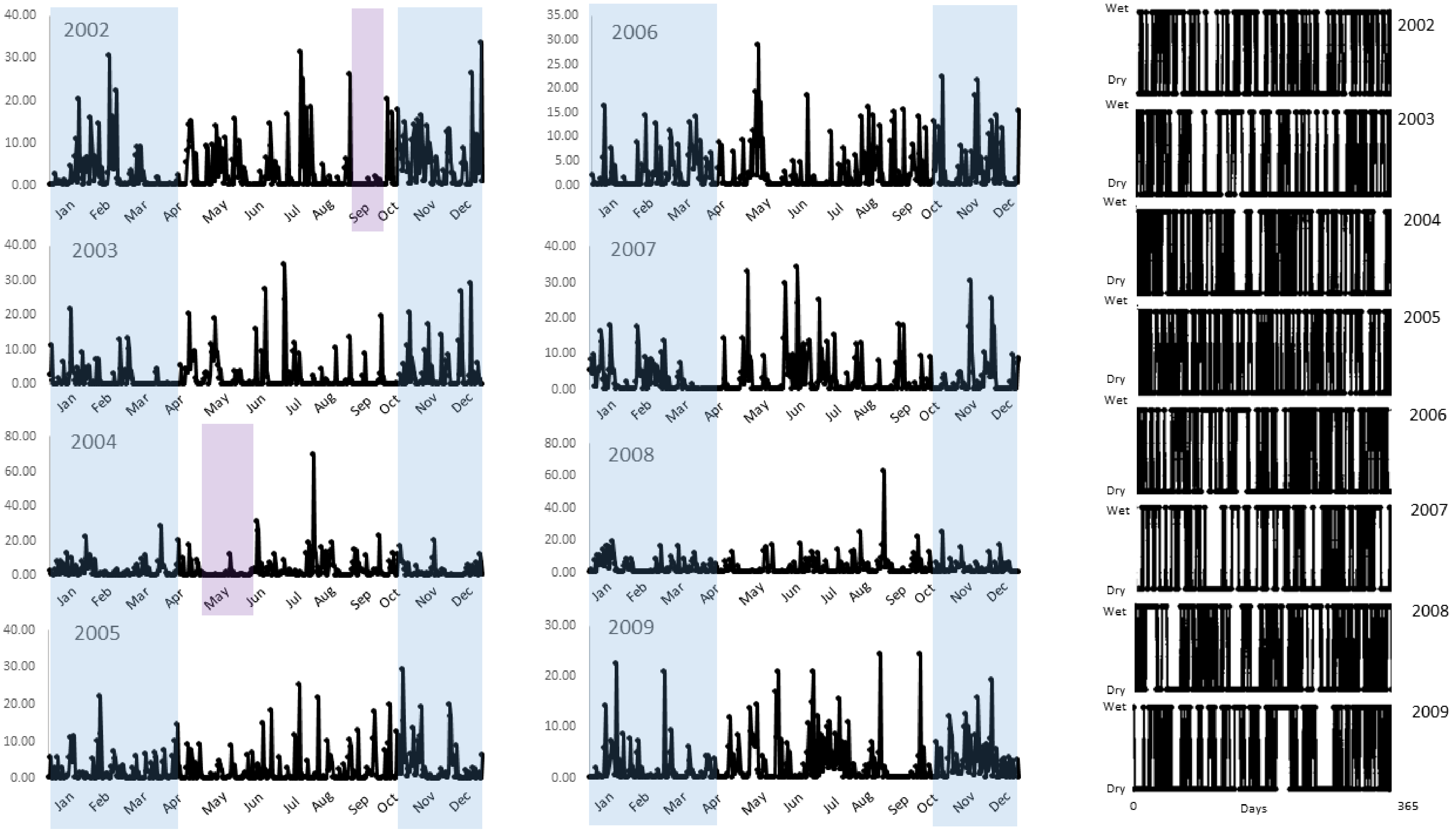

Supplementary Figure 5: Rainfall data for the Ballidon area 2002 – 2009 summarised from publically available Met office data from the Middleton Hillside (weather station 6.5 km from Ballidon). Data highlighted blue = winter months where the earthworks is continually wet and highlighted purple are extended dry periods in the summer where the earthworks may have dried out.

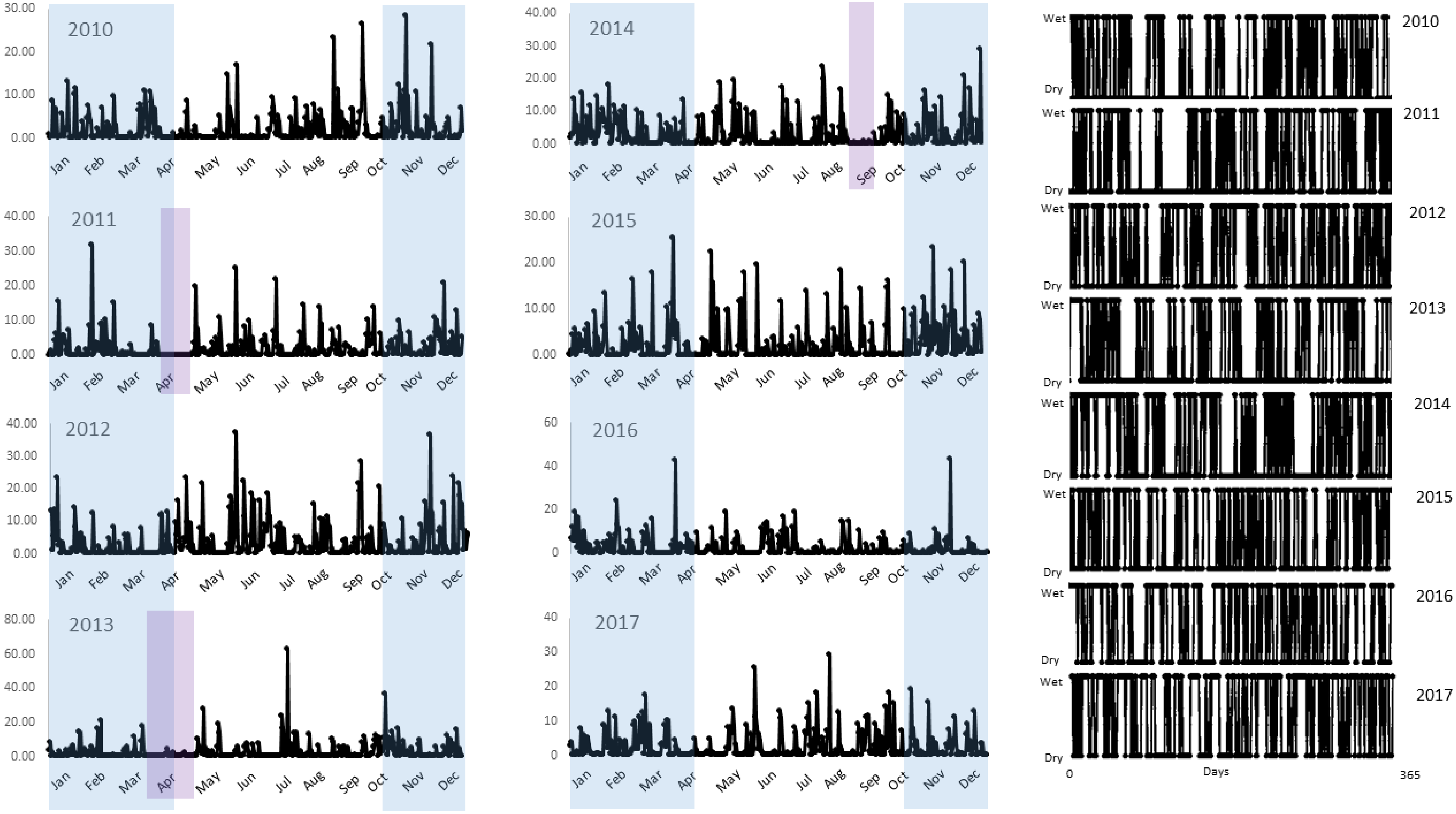

Supplementary Figure 6: Rainfall data for the Ballidon area 2010 – 2017 summarised from publically available Met office data from the Middleton Hillside (weather station 6.5 km from Ballidon). Data highlighted blue = winter months where the earthworks is continually wet and highlighted purple are extended dry periods in the summer where the earthworks may have dried out.

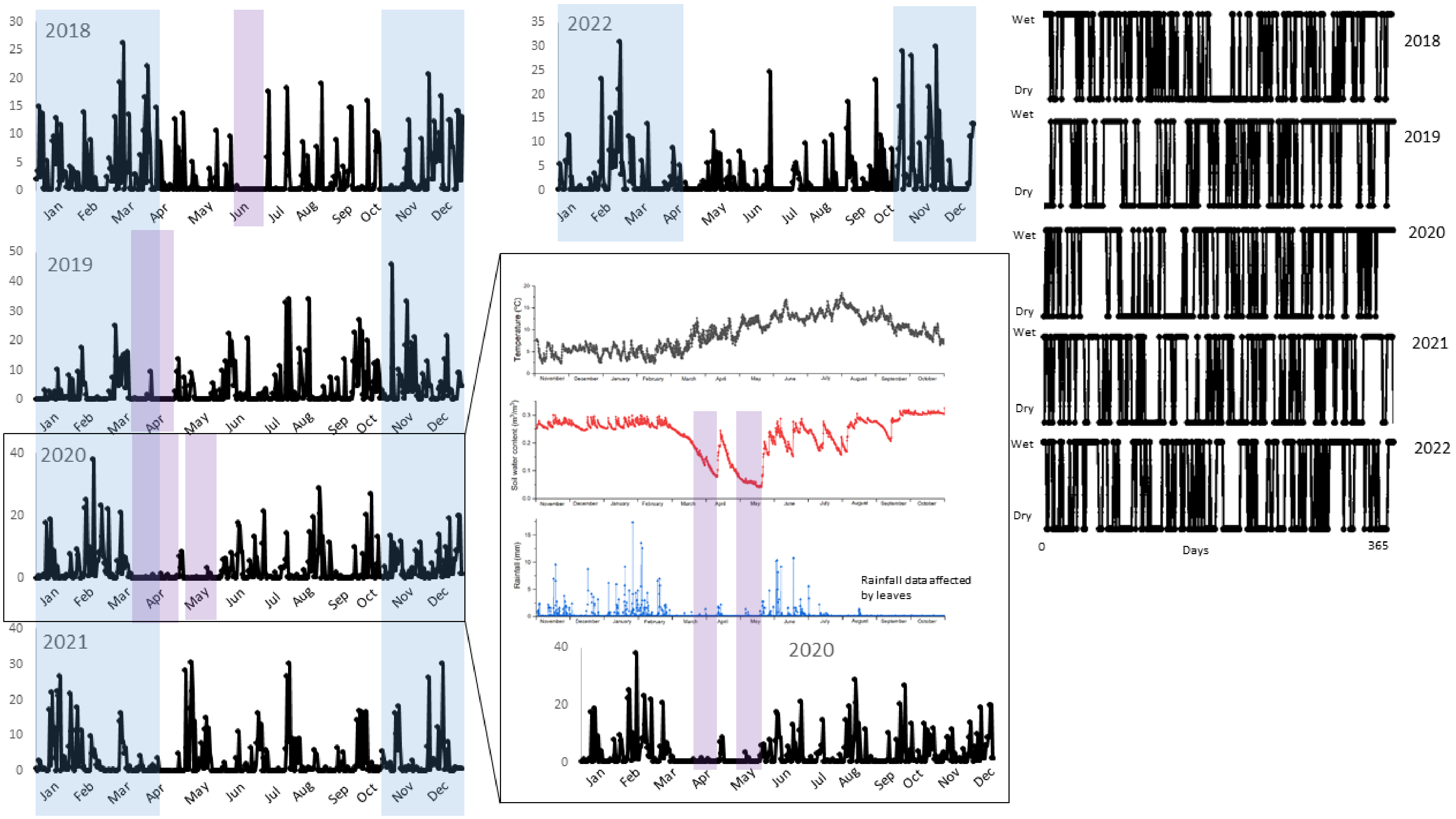

Supplementary Figure 7: Rainfall data for the Ballidon area 2018 – 2022 summarised from publically available Met office data from the Middleton Hillside (weather station 6.5 km from Ballidon). Data highlighted blue = winter months where the earthworks is continually wet and highlighted purple are extended dry periods in the summer where the earthworks may have dried out. Also inset is the comparison of data collected during 2020 by the onsite weather station compared to data from nearby weather stations.

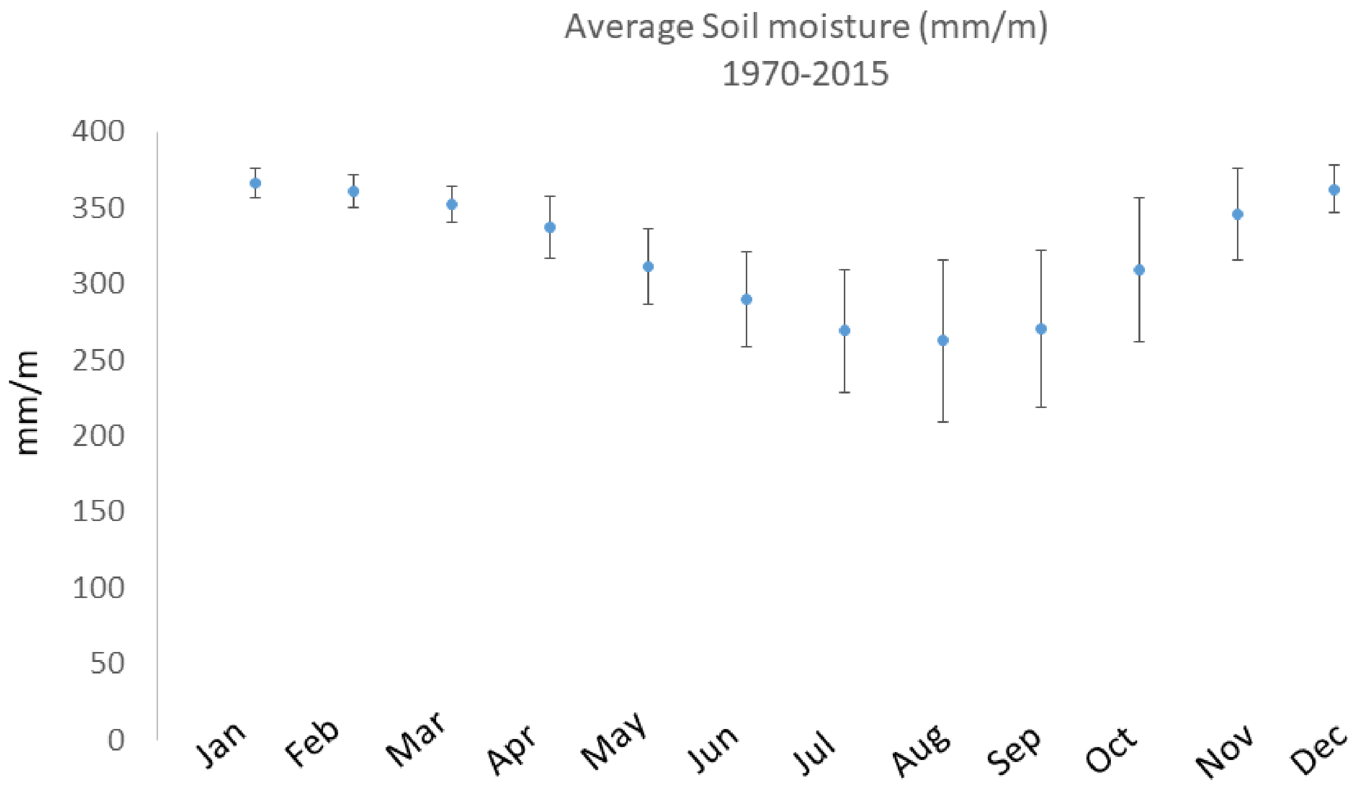

Supplementary Figure 8: Average soil moisture for the Ballidon area obtained from: Met Office (2018): HadUK-Grid gridded and regional average climate observations for the UK. Centre for Environmental Data Analysis, date of citation. http://catalogue.ceda.ac.uk/uuid/4dc8450d889a491ebb20e724debe2dfb

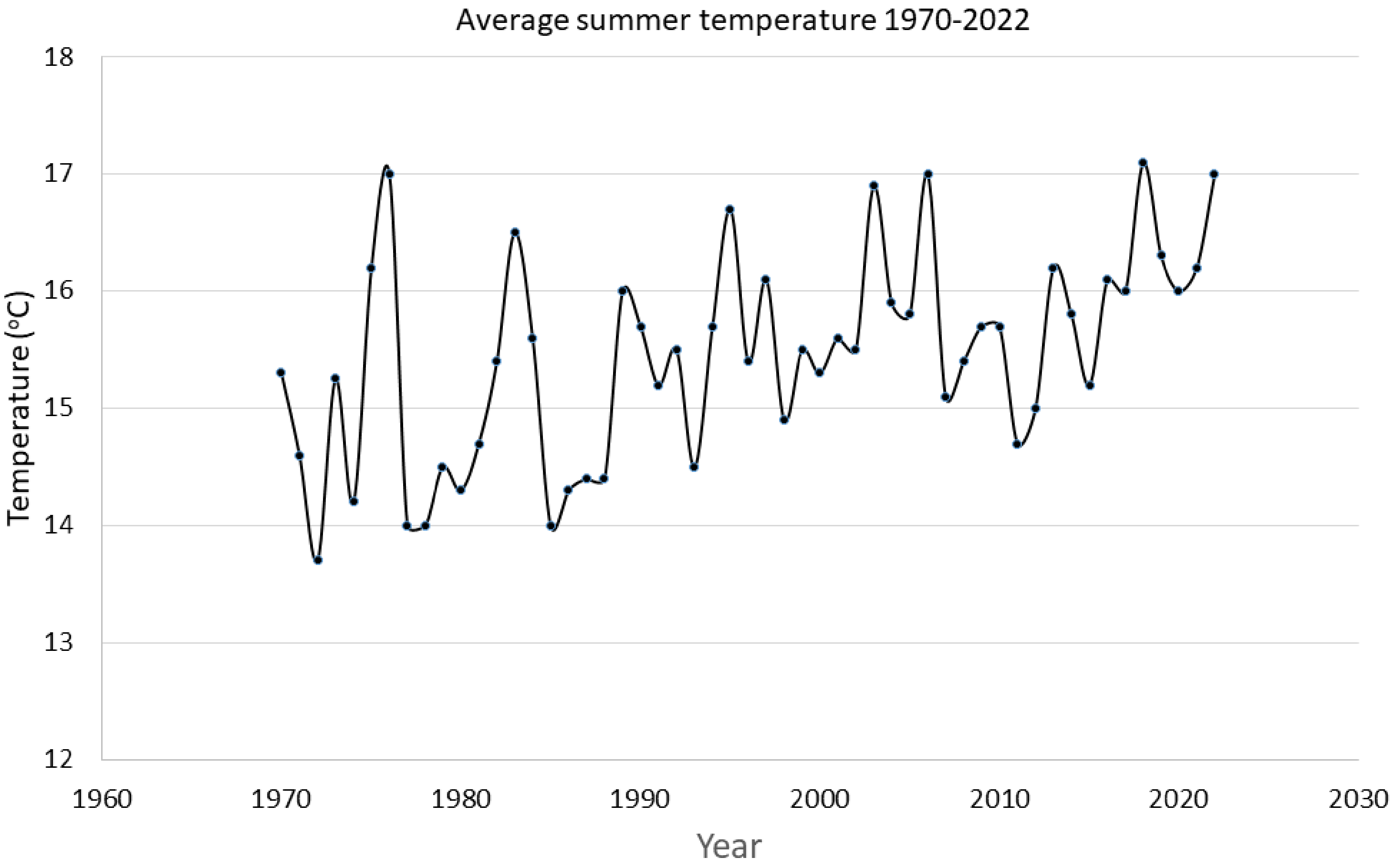

Supplementary Figure 9: Average summer temperature for the UK adapted from: Met Office; Hollis, D.; McCarthy, M.; Kendon, M.; Legg, T. (2023): HadUK-Grid Gridded Climate Observations on a 60km grid over the UK, v1.2.0.ceda (1836-2022). NERC EDS Centre for Environmental Data Analysis, 30 August 2023. doi:10.5285/22df6602b5064b1686dda7e9455f86fc.

Glass 1 - Roman

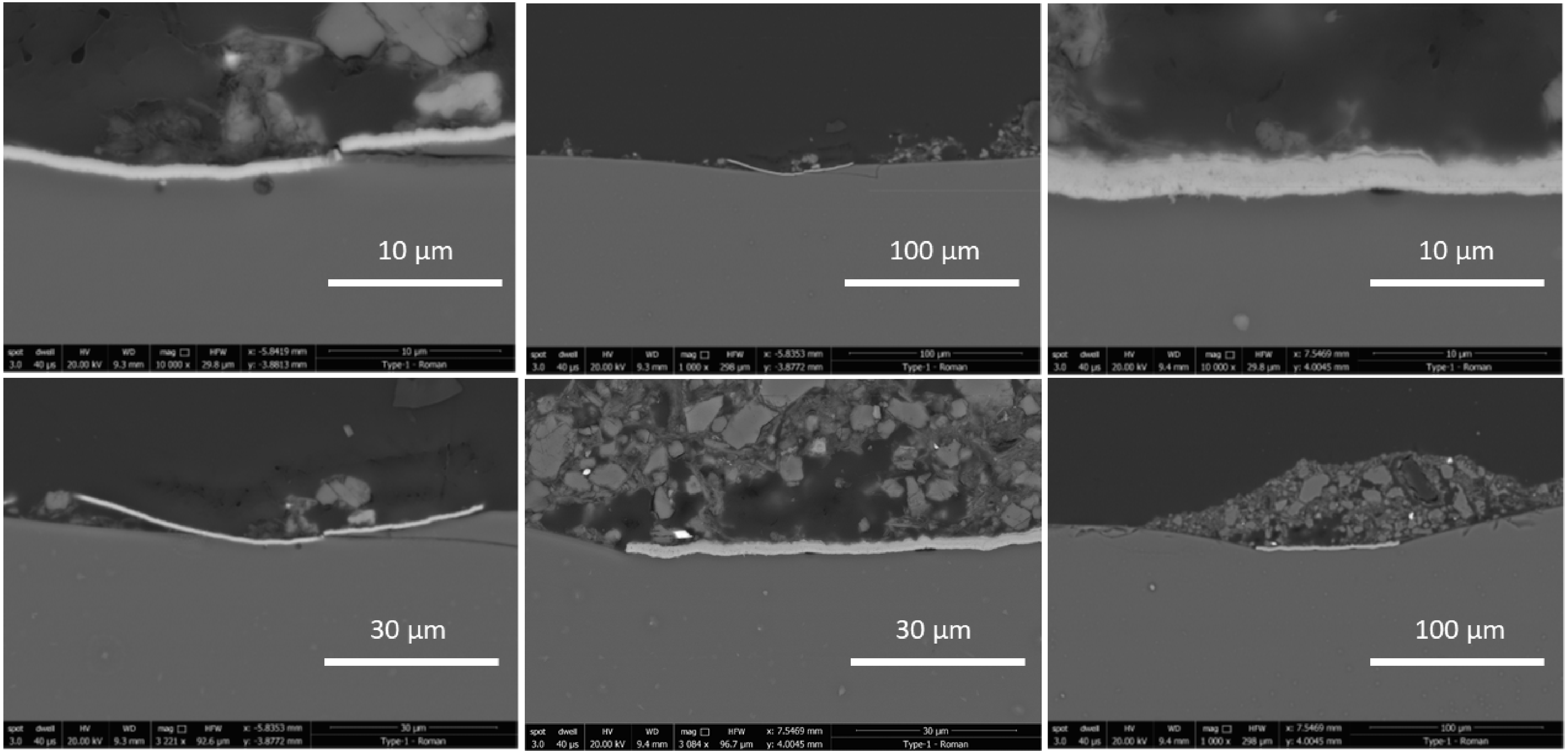

Supplementary Figure 10: SEM-Backscatter detection images from Glass 1 (Roman).

Glass 2 – Medieval

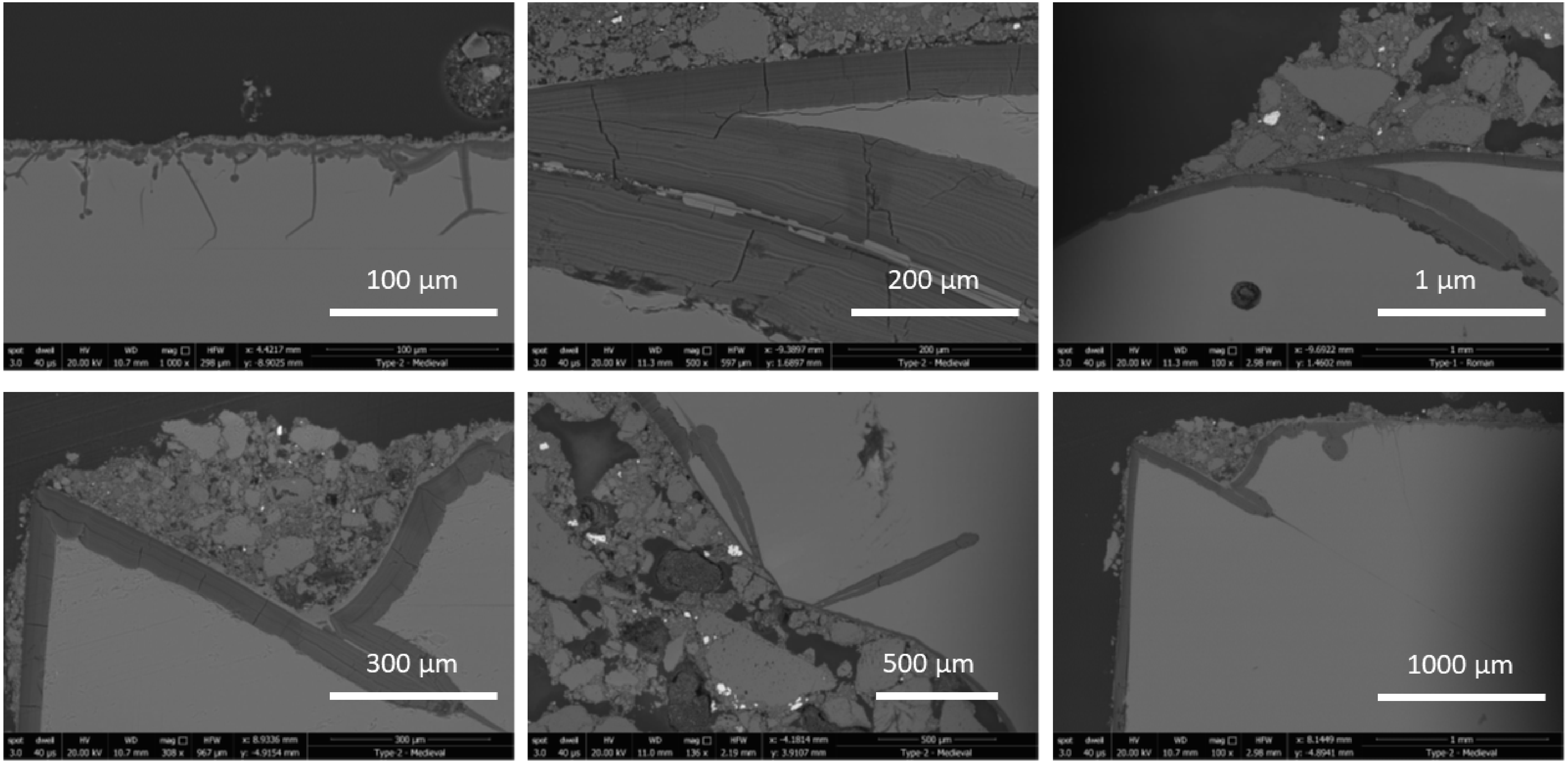

Supplementary Figure 11: SEM-Backscatter detection images from Glass 2 (Medieval).

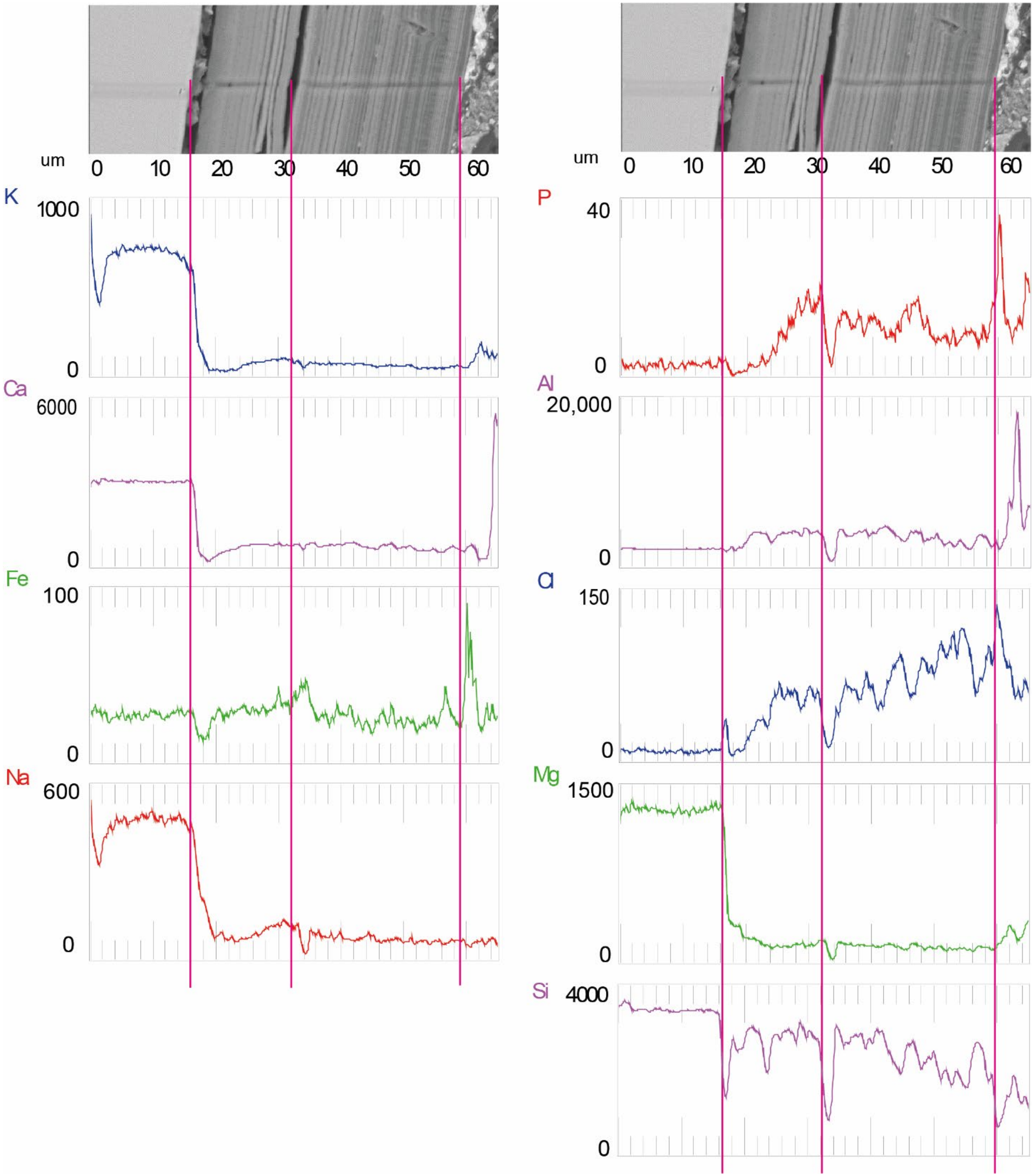

Supplementary Figure 12: Line scan across the alteration layer of Glass 2 showing the variation for key elements (measured in counts). This data is semi-quantitive and indicative of the relative abundance of each elements across the alteration layer.

Glass 3 – Hangleton Linen Smoother

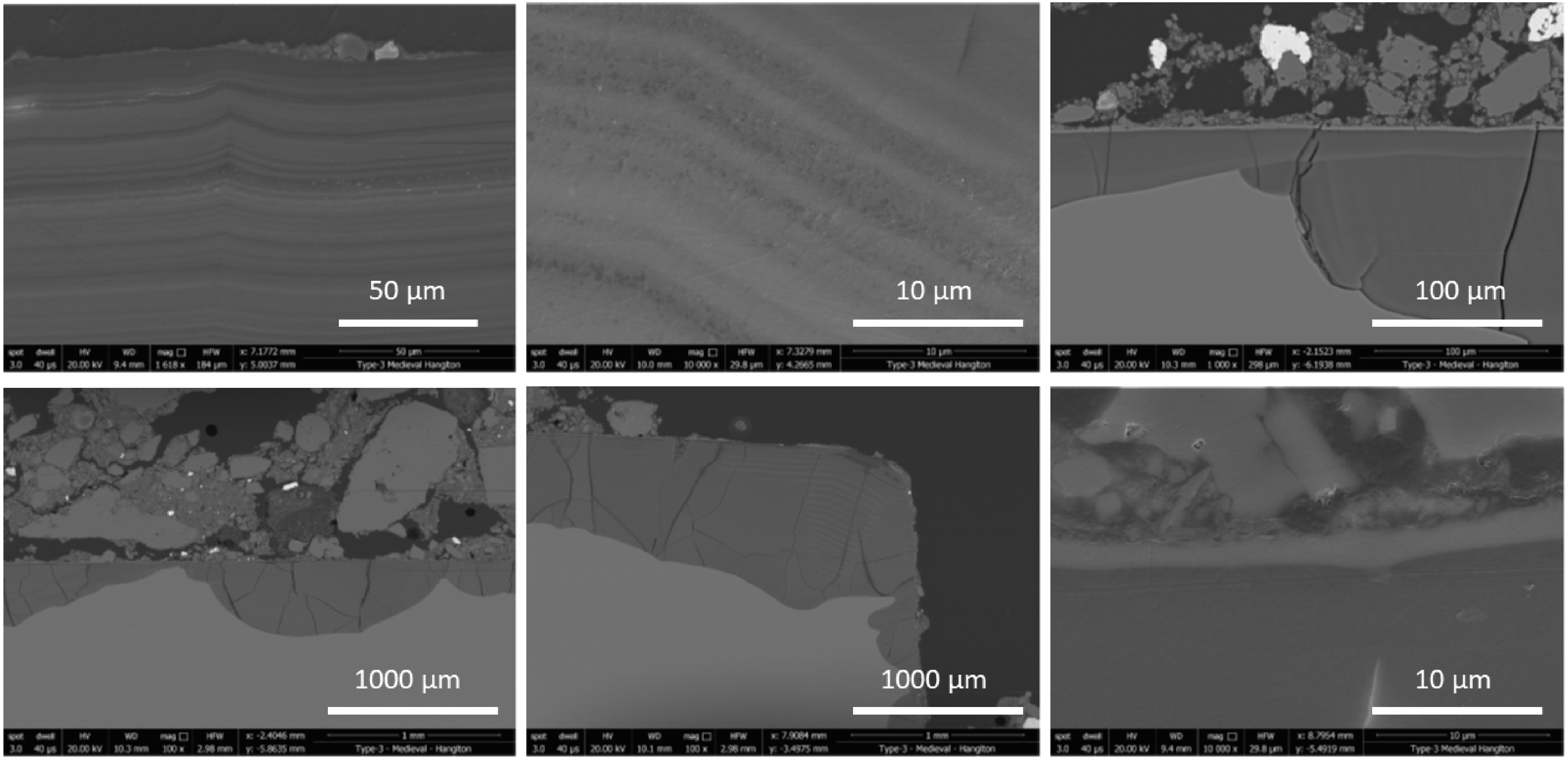

Supplementary Figure 13: SEM-Backscatter detection images from Glass 3 (Hangleton).

Glass 4 – Plate Glass

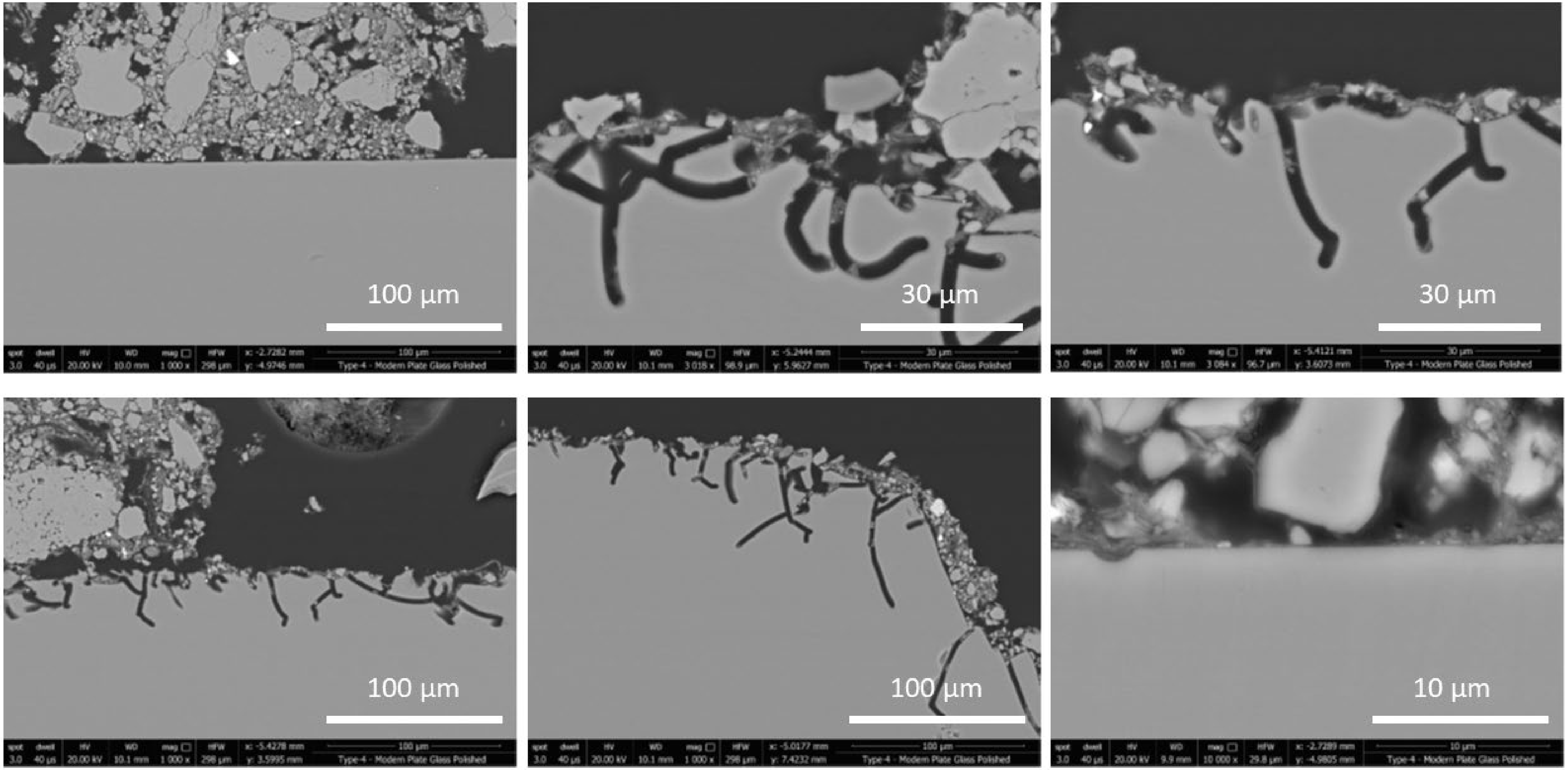

Supplementary Figure 14: SEM-Backscatter detection images from Glass 4 (Plate glass).

Glass 5 – Plate Glass

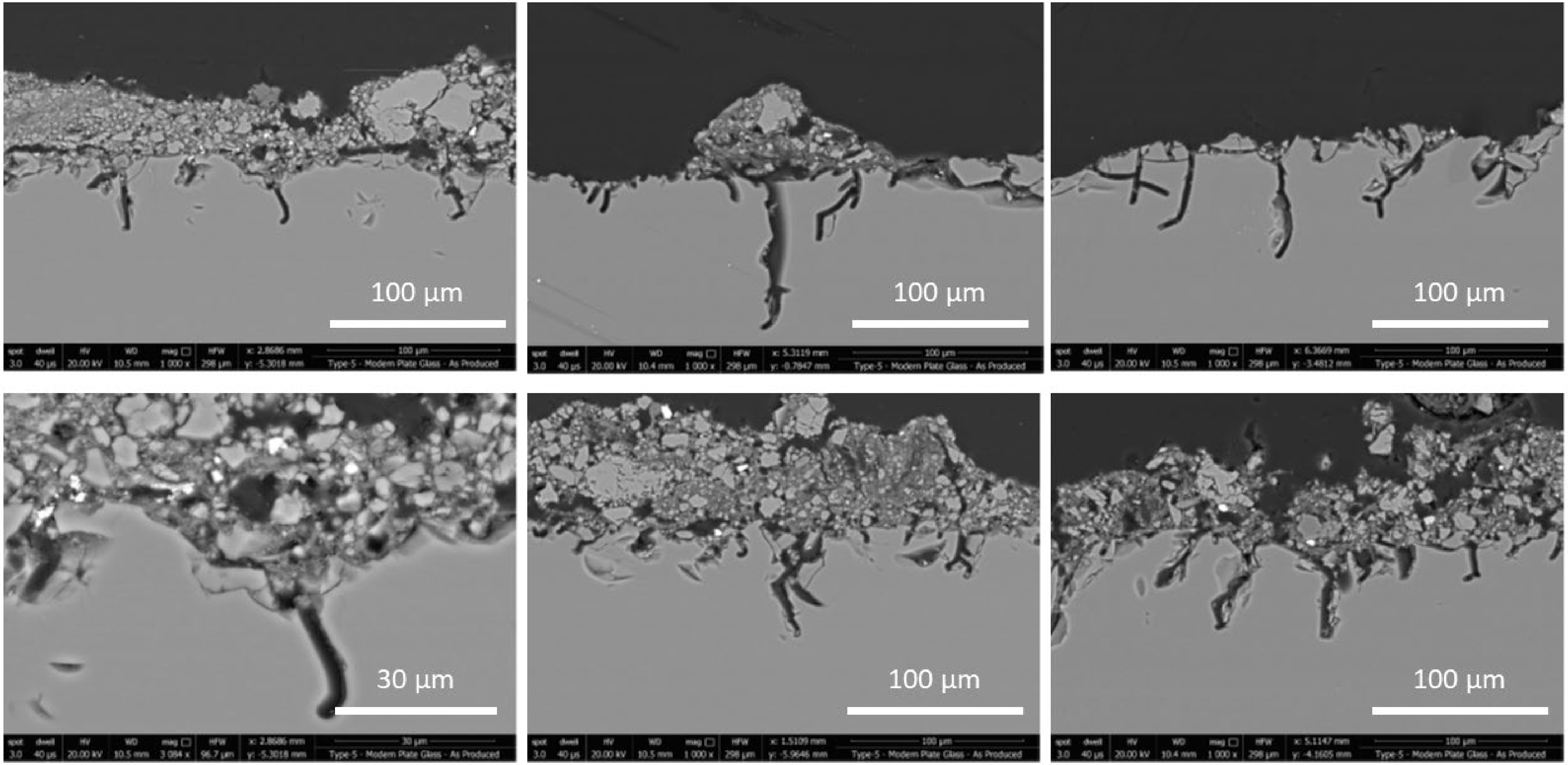

Supplementary Figure 15: SEM-Backscatter detection images from Glass 5 (Plate glass).

Glass 6 – E glass

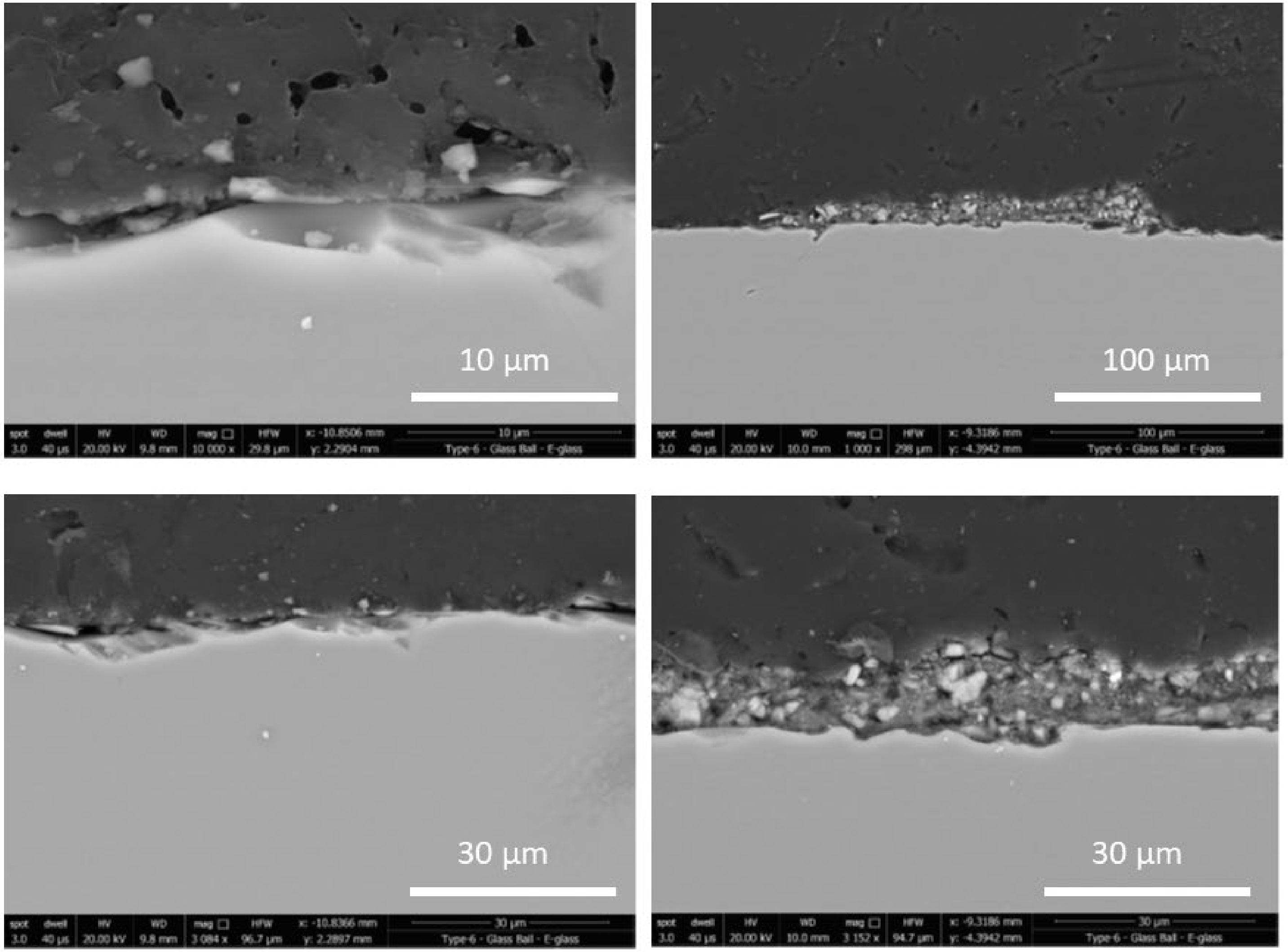

Supplementary Figure 16: SEM-Backscatter detection images from Glass 6 (E-Glass marble).

Glass 7 – Borosilicate

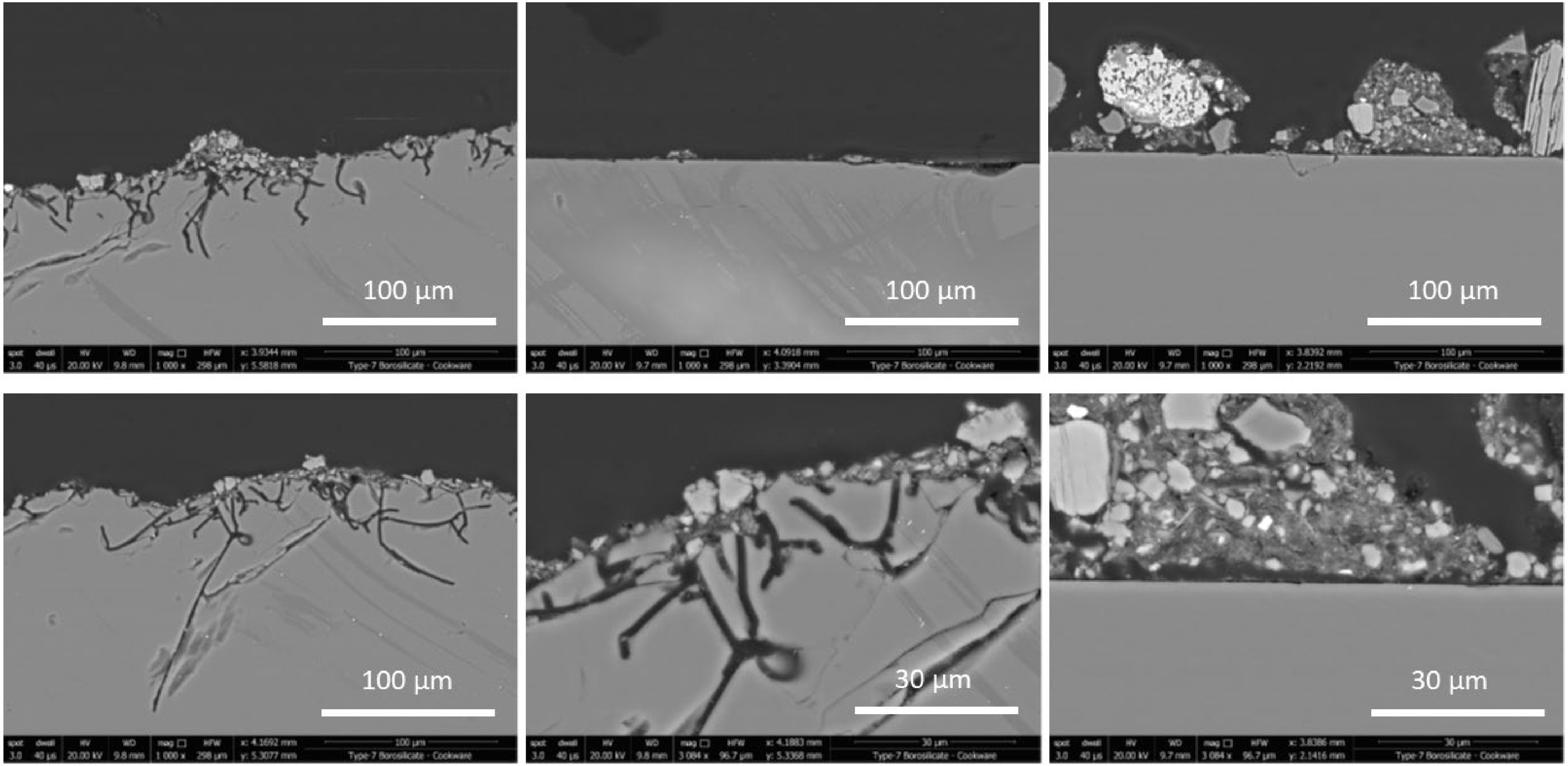

Supplementary Figure 17: SEM-Backscatter detection images from Glass 7 (Borosilicate).

Glass 7 – Borosilicate

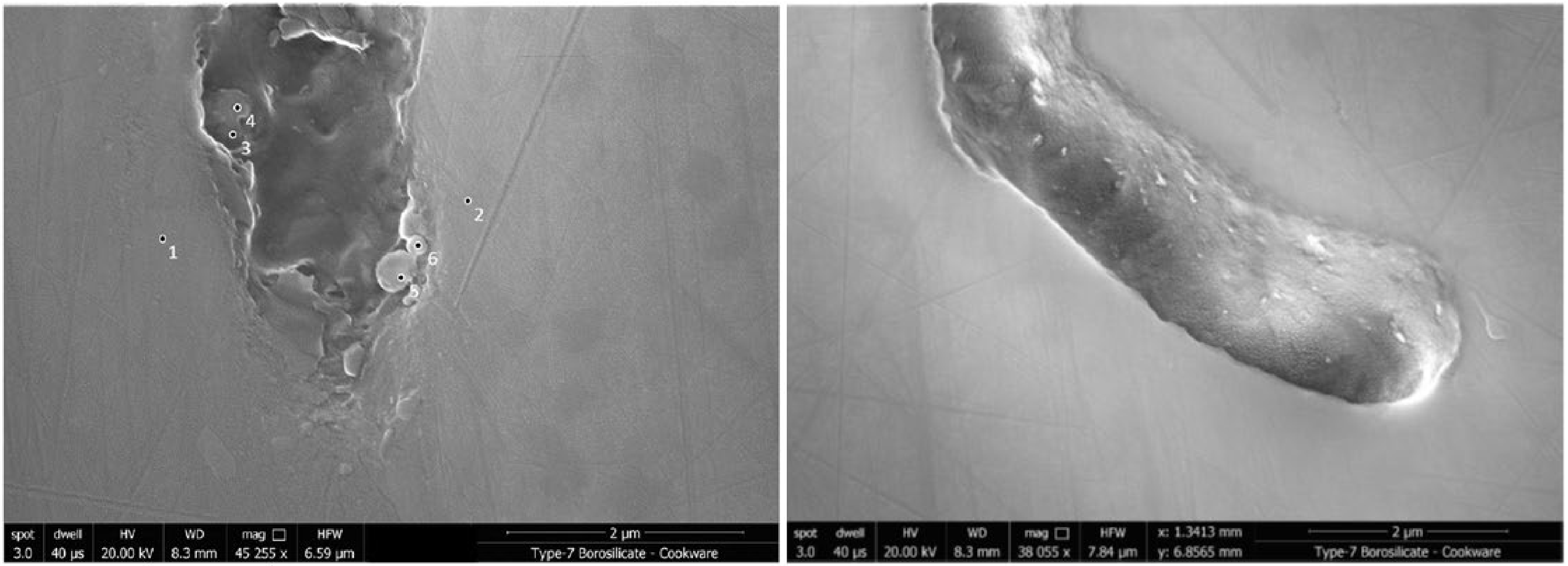

Spot	Description	Element At. %								
		Si	Al	Na	K	Mg	Ca	P	Cl	Fe
1	Pristine	90.2 ± 0.9	4.0 ± 0.4	2.3 ± 0.2	0.60 ± 0.06	-	1.7 ± 0.2	-	0.53 ± 0.05	0.73 ± 0.07
2	Pristine	94.0 ± 9.4	3.0 ± 0.3	1.8 ± 0.2	0.28 ± 0.03	-	0.78 ± 0.08	-	-	0.28 ± 0.03
3	Precipitate	63.8 ± 6.4	7.0 ± 0.7	1.8 ± 0.2	1.9 ± 0.2	0.51 ± 0.05	21.6 ± 2.2	-	1.0 ± 0.1	2.6 ± 0.3
4	Precipitate	64.0 ± 6.4	7.0 ± 0.7	1.6 ± 0.2	1.7 ± 0.2	0.62 ± 0.06	20.1 ± 2.0	1.2 ± 0.1	1.0 ± 0.1	2.8 ± 0.3
5	Precipitate	84.8 ± 8.5	4.2 ± 0.4	1.8 ± 0.2	0.76 ± 0.08	-	7.0 ± 0.7	-	0.69 ± 0.07	0.83 ± 0.08
6	Precipitate	88.4 ± 8.8	3.5 ± 0.4	1.6 ± 0.2	0.42 ± 0.04	-	4.6 ± 0.5	-	0.85 ± 0.08	0.64 ± 0.06

Supplementary Figure 18: SEM-Secondary electron image from Glass 7 (Borosilicate) and EDX of spot analysis.

Glass 8 – Soda lime Optical

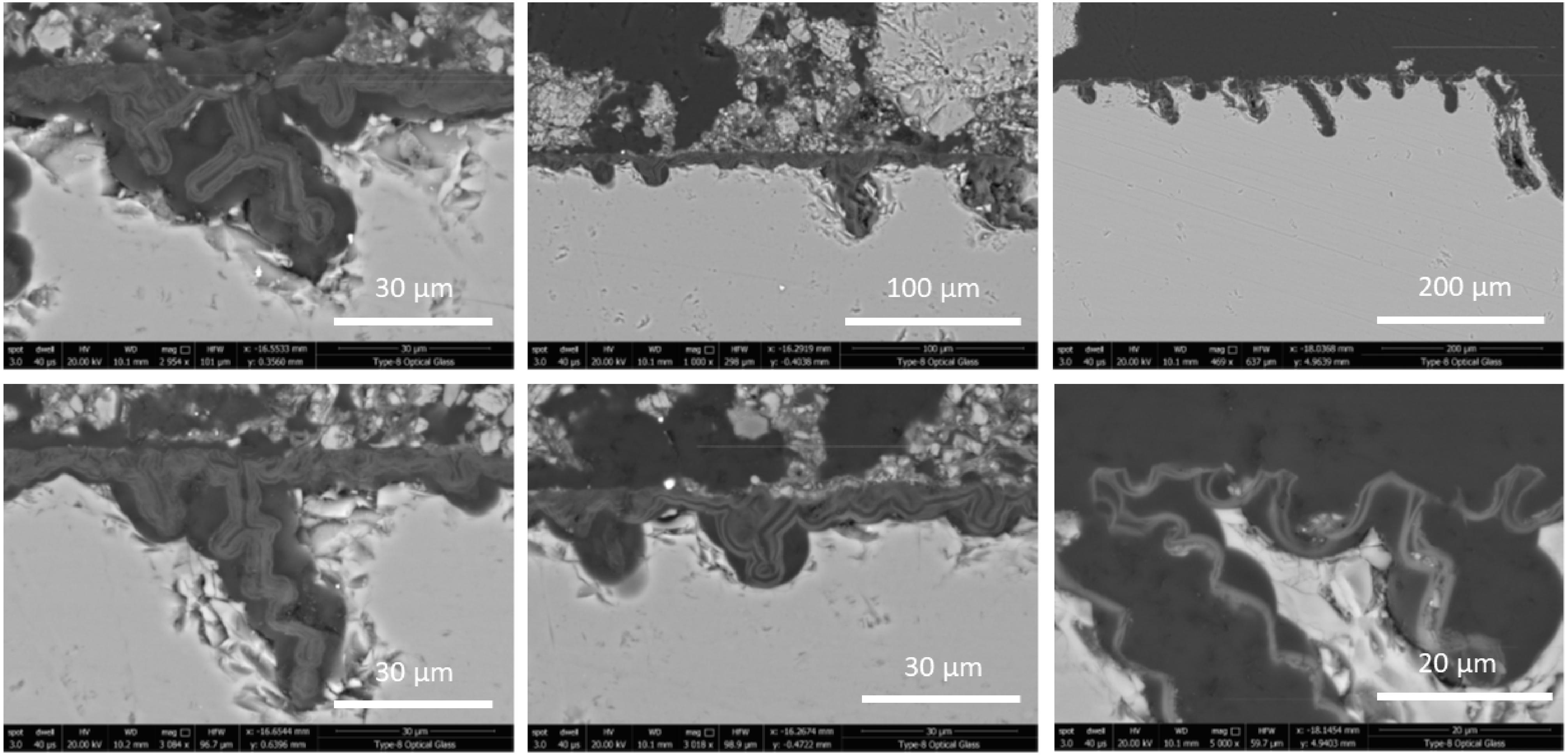

Supplementary Figure 19: SEM-Backscatter detection images from Glass 8 (Soda Lime Optical).

Glass 9 – Lead Optical

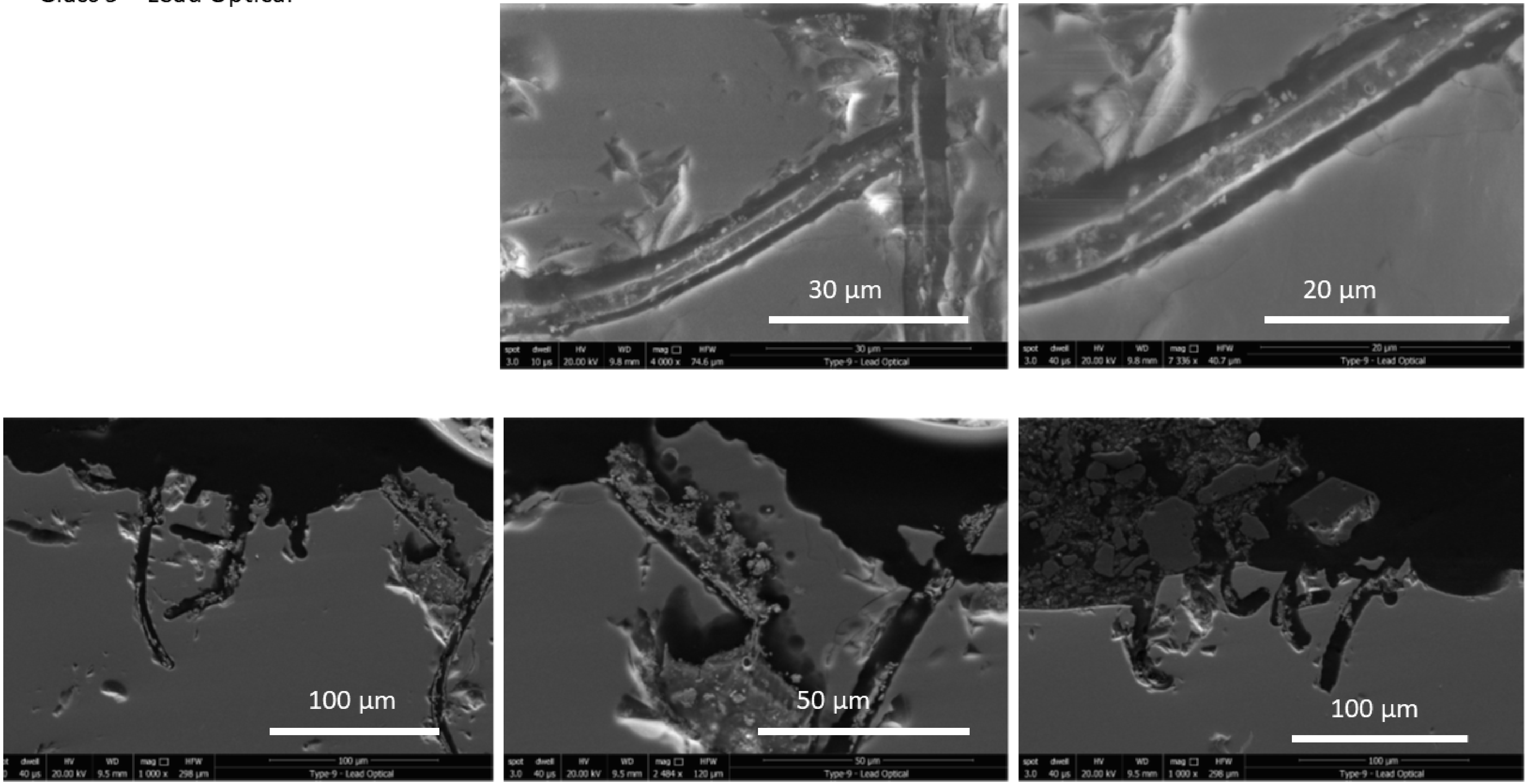

Supplementary Figure 20: SEM-Backscatter detection images from Glass 9 (Lead Optical).

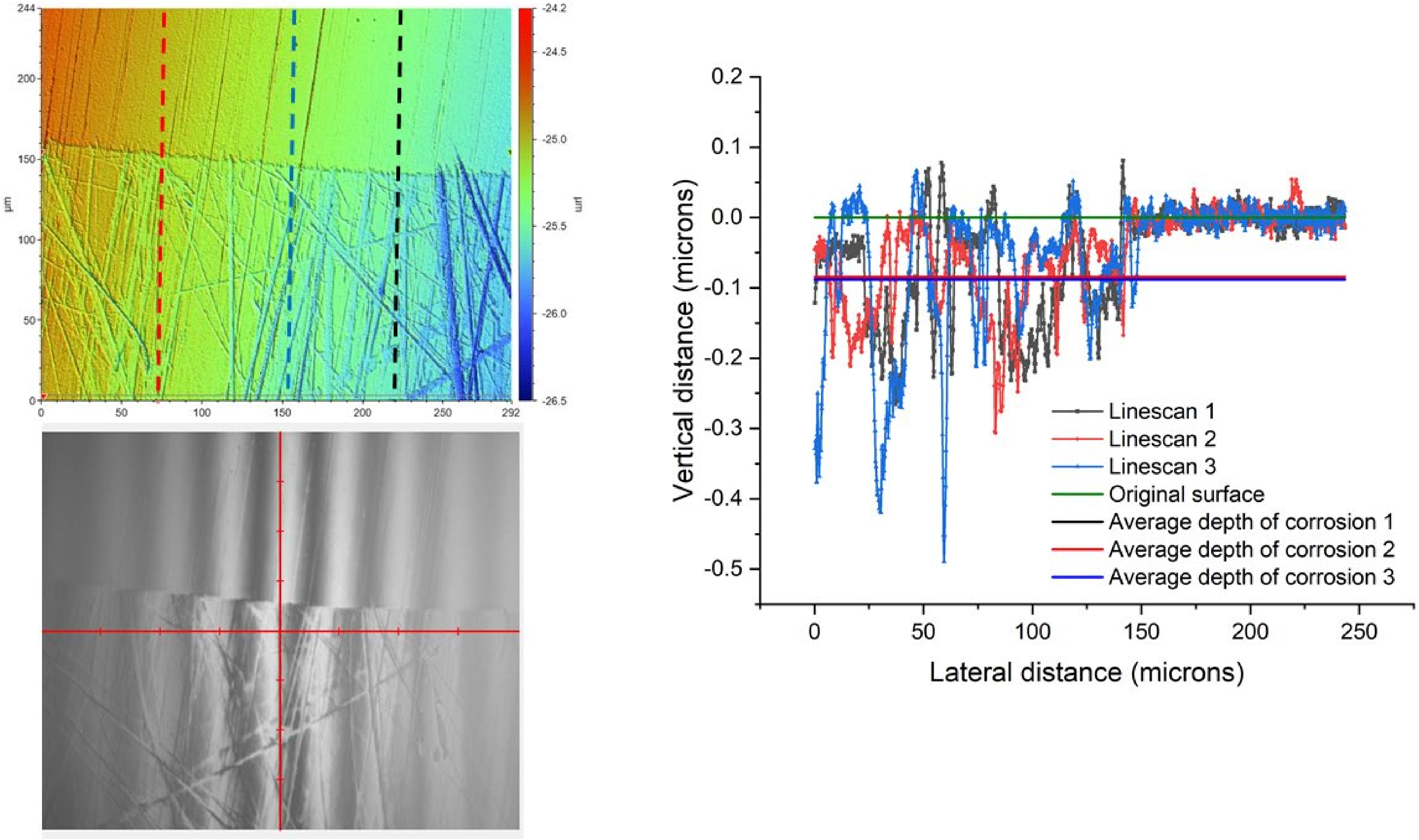

Supplementary Figure 21: Step height for Glass 3, Hangleton Linen Smoother, exposed to SRCA testing at pH 8.2, 10 ^{o}C for 28 days.

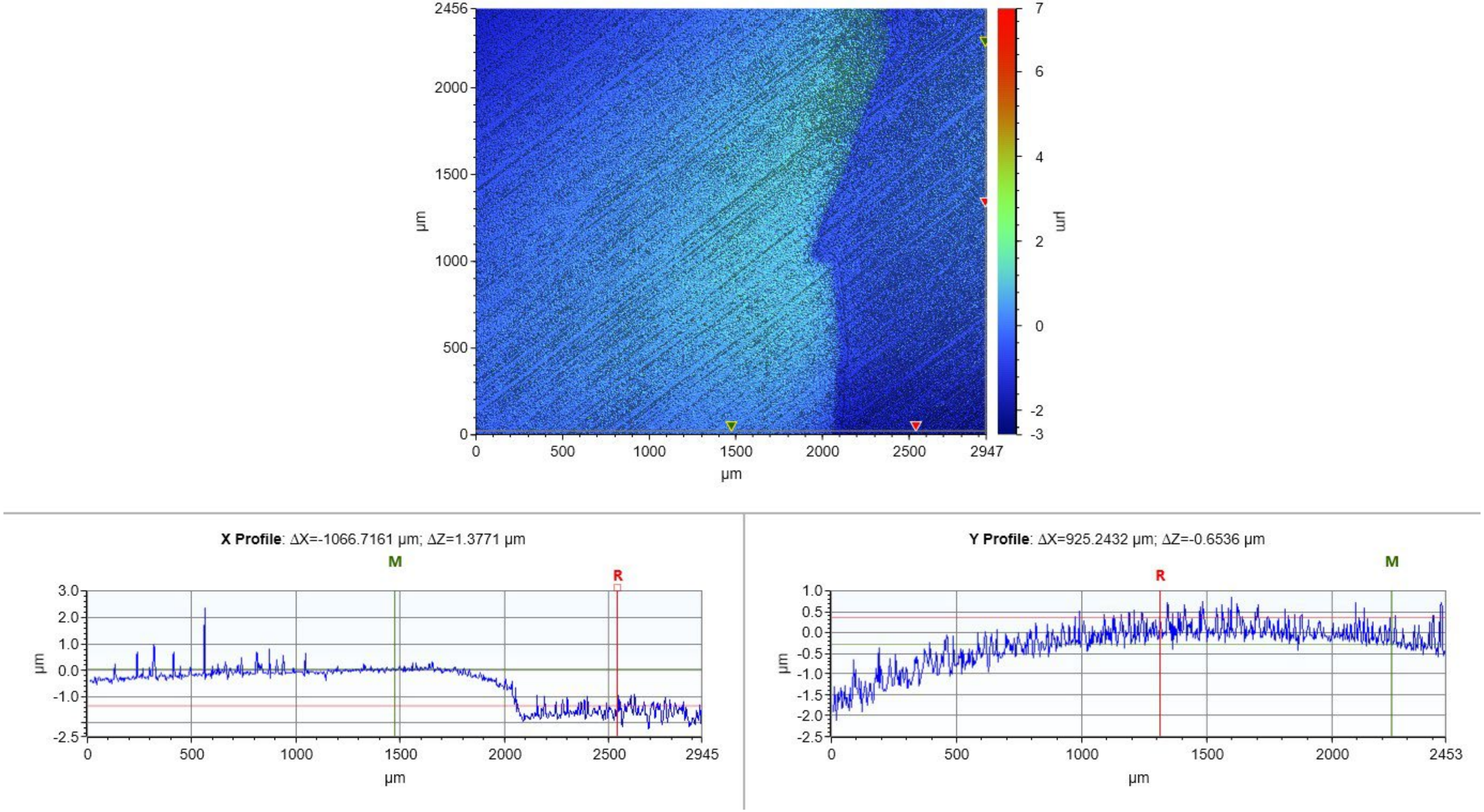

Supplementary Figure 22: measurement of step height by vertical scanning interferometry; average sample height are taken either side of the step (X profile).

Supplementary Table 1: Results by class of 16S rRNA analysis on glass samples from the mid-earthworks level, swabbed limestone clast and finer sediment particles.

Total reads	60861	52882	57741	35920	48588	78727	95637	73548	57517	77620	54563	27383	55592
**Sample from with swab was taken**	**Soda Lime Optical**	**Lead Optical**	**E-Glass**	**Plate glass Unpolished**	**Hangleton Linen smoother**	**Medieval**	**Borosilicate**	**Roman**	**Plate glass polished**	**Limestone**	**Sediment**	**Sediment**	**Sediment**
Unclassified	11.12%	11.82%	14.48%	10.01%	13.85%	12.12%	10.73%	12.35%	11.91%	12.68%	6.97%	0.43%	12.11%
Nitrososphaeria	1.06%	1.71%	1.31%	2.10%	1.81%	1.28%	1.63%	4.08%	2.28%	1.94%	1.28%	0.17%	1.82%
Acidobacteriae	0.40%	0.34%	0.25%	0.18%	0.22%	0.80%	0.30%	0.45%	0.12%	0.37%	0.23%	0.00%	0.33%
Blastocatellia	3.92%	2.19%	1.59%	2.15%	2.57%	2.40%	1.42%	2.76%	2.08%	1.65%	0.69%	0.05%	1.17%
Holophagae	0.46%	0.87%	0.08%	0.28%	0.39%	0.23%	0.31%	0.87%	0.07%	0.18%	0.71%	0.00%	0.48%
Thermoanaerobaculia	0.39%	0.36%	0.57%	0.38%	0.81%	0.61%	0.44%	0.26%	0.19%	0.32%	0.21%	0.03%	0.23%
Vicinamibacteria	18.36%	26.63%	25.67%	16.05%	21.58%	19.96%	22.49%	17.18%	22.62%	21.94%	27.77%	1.55%	27.10%
Acidimicrobiia	0.84%	0.82%	0.50%	0.77%	0.86%	1.21%	1.39%	0.62%	1.09%	1.03%	1.03%	0.00%	1.01%
Actinobacteria	1.87%	1.80%	0.97%	1.22%	1.05%	1.35%	1.77%	2.05%	1.68%	1.54%	0.94%	0.03%	0.89%
Thermoleophilia	2.27%	2.72%	1.26%	0.96%	2.72%	2.50%	3.20%	1.73%	2.81%	2.76%	1.10%	0.07%	1.94%
Bacteroidia	2.53%	1.67%	2.44%	4.57%	1.59%	2.79%	3.12%	2.10%	2.25%	1.89%	5.63%	0.16%	2.38%
Anaerolineae	2.46%	1.90%	2.00%	3.60%	2.57%	3.02%	1.52%	3.28%	2.19%	2.11%	1.55%	0.27%	1.90%
Chloroflexia	0.31%	0.46%	1.02%	1.27%	0.44%	0.90%	0.29%	1.12%	0.98%	1.28%	0.48%	0.00%	0.30%
Dehalococcoidia	0.49%	0.40%	0.76%	0.23%	0.59%	0.52%	0.34%	0.39%	0.37%	0.41%	0.25%	0.00%	0.35%
Desulfobacteria	0.00%	0.00%	0.00%	0.00%	0.00%	0.00%	0.00%	0.00%	0.00%	0.00%	0.23%	0.00%	0.00%
Desulfuromonadia	0.06%	0.01%	0.34%	6.83%	0.00%	0.09%	0.07%	0.00%	0.09%	0.06%	0.07%	4.55%	0.03%
Uncultured Desulfobacterota	0.57%	0.38%	0.36%	0.44%	0.58%	0.46%	0.08%	0.45%	0.29%	0.30%	0.17%	0.00%	0.06%
Entotheonellia	0.27%	0.16%	0.11%	0.08%	0.06%	0.09%	0.24%	0.11%	0.15%	0.16%	0.05%	0.00%	0.00%
Bacilli	0.48%	0.79%	0.56%	1.34%	0.24%	0.35%	0.66%	0.27%	0.43%	1.40%	6.66%	5.80%	6.27%
Clostridia	0.34%	0.00%	3.17%	15.87%	0.06%	0.09%	0.08%	0.04%	0.14%	0.13%	2.03%	14.18%	0.48%
Gemmatimonadetes	1.12%	0.84%	1.03%	1.34%	2.53%	4.36%	0.84%	3.37%	1.75%	1.90%	0.60%	0.00%	0.98%
Halanaerobiia	0.12%	0.00%	0.08%	0.08%	0.00%	0.00%	0.00%	0.00%	0.00%	0.00%	0.60%	1.48%	0.94%
Latescibacteria	0.63%	0.40%	0.24%	0.31%	0.34%	0.48%	0.58%	0.22%	0.55%	0.67%	1.15%	0.11%	0.58%
Latescibacterota	1.89%	1.63%	3.16%	0.64%	2.66%	1.67%	1.95%	3.26%	2.52%	2.12%	1.33%	0.29%	3.20%
Methylomirabilia	4.23%	3.72%	4.48%	2.78%	4.42%	5.25%	2.72%	5.77%	4.17%	4.81%	2.29%	0.25%	4.01%
Myxococcia	0.13%	0.19%	0.24%	0.10%	0.26%	0.05%	0.14%	0.03%	0.09%	0.03%	0.09%	0.00%	0.38%
Polyangia	2.04%	1.58%	1.37%	0.97%	1.16%	1.01%	1.67%	1.29%	1.11%	1.55%	0.91%	0.15%	1.32%
Nitrospiria	1.03%	1.71%	1.70%	1.10%	1.67%	1.76%	1.17%	1.96%	1.19%	1.48%	0.46%	0.10%	1.16%
Phycisphaerae	1.13%	0.86%	1.08%	0.50%	1.11%	0.61%	0.81%	0.92%	1.08%	0.98%	0.32%	0.00%	0.92%
Planctomycetes	6.18%	5.31%	6.76%	2.07%	5.31%	7.09%	6.57%	5.04%	5.84%	6.38%	3.65%	0.22%	4.85%
Alphaproteobacteria	9.95%	8.03%	6.23%	7.86%	10.84%	9.87%	10.18%	8.20%	9.41%	8.74%	6.45%	0.33%	6.40%
Gammaproteobacteria	14.74%	13.20%	10.60%	11.06%	12.31%	13.07%	14.40%	14.81%	13.17%	11.98%	21.45%	69.69%	12.52%
Verrucomicrobiae	8.60%	7.49%	5.62%	2.87%	5.39%	4.02%	8.90%	5.04%	7.38%	7.20%	2.68%	0.10%	3.90%
	100.00%	100.00%	100.00%	100.00%	100.00%	100.00%	100.00%	100.00%	100.00%	100.00%	100.00%	100.00%	100.00%

Supplementary Table 2: Results by class of 16S rRNA analysis on glass samples from the lower-earthworks level, swabbed limestone clast and finer sediment particles.

Total reads	62893	57172	68572	68103	77993	57132	40310	67214	71481	77620	47425	69313	55367
**Sample from with swab was taken**	**Soda lime optical**	**Lead optical**	**e-glass**	**Plate Unpolished**	**Plate polished**	**Medieval**	**Hangleton Linen Smoother**	**Roman**	**Borosilicate**	**Limestone**	**Soil**	**Soil**	**Soil**
Unclassified	14.09%	11.44%	13.11%	15.38%	16.35%	12.70%	15.00%	13.33%	13.54%	12.68%	7.88%	12.04%	11.50%
Nitrososphaeria	2.42%	2.47%	2.99%	2.44%	2.14%	3.37%	2.86%	5.31%	3.16%	1.94%	2.14%	4.79%	4.16%
Acidobacteriae	1.01%	0.66%	0.61%	0.76%	0.73%	0.28%	1.22%	0.39%	0.51%	0.37%	0.32%	0.18%	0.23%
Blastocatellia	3.09%	1.82%	0.97%	2.54%	2.89%	2.20%	1.73%	1.91%	1.24%	1.65%	1.22%	1.43%	1.50%
Holophagae	0.51%	0.52%	0.21%	0.29%	0.72%	0.43%	0.62%	0.23%	0.28%	0.18%	0.33%	0.37%	0.30%
Thermoanaerobaculia	0.12%	0.92%	0.35%	0.35%	0.35%	1.12%	3.79%	0.87%	1.04%	0.32%	0.19%	0.47%	0.45%
Vicinamibacteria	22.29%	18.68%	21.53%	22.24%	24.11%	22.51%	18.14%	21.40%	19.46%	21.94%	14.73%	17.92%	13.95%
Acidimicrobiia	0.77%	0.68%	0.60%	0.47%	0.57%	1.05%	0.46%	0.85%	0.60%	1.03%	1.02%	1.64%	0.93%
Actinobacteria	1.67%	1.28%	1.43%	0.84%	0.77%	1.13%	0.71%	1.19%	1.10%	1.54%	0.94%	3.06%	1.66%
Thermoleophilia	1.66%	2.34%	1.95%	1.71%	2.23%	2.59%	2.58%	2.86%	2.13%	2.76%	1.74%	5.73%	2.49%
Bacteroidia	2.44%	2.21%	1.46%	1.08%	0.98%	0.94%	1.27%	1.22%	1.87%	1.89%	13.66%	1.15%	1.09%
Anaerolineae	3.69%	2.50%	3.05%	3.01%	1.37%	3.32%	2.71%	2.66%	2.80%	2.11%	1.38%	0.95%	1.25%
Chloroflexia	0.74%	0.20%	0.73%	1.13%	0.46%	0.94%	0.28%	0.45%	0.49%	1.28%	0.40%	0.66%	0.31%
Dehalococcoidia	0.78%	0.61%	0.68%	0.50%	0.76%	0.82%	1.34%	0.50%	0.33%	0.41%	0.00%	0.25%	0.40%
Desulfobacteria	0.00%	0.00%	0.00%	0.00%	0.00%	0.00%	0.00%	0.00%	0.00%	0.00%	0.78%	0.00%	0.00%
Desulfuromonadia	0.04%	0.13%	0.18%	0.09%	0.12%	0.27%	0.40%	0.35%	0.15%	0.06%	0.54%	0.40%	0.80%
Uncultured Desulfobacterota	0.97%	0.50%	0.59%	0.53%	0.50%	0.52%	0.48%	0.48%	0.36%	0.30%	0.13%	0.66%	0.40%
Entotheonellia	0.22%	0.16%	0.20%	0.14%	0.58%	0.16%	0.10%	0.10%	0.22%	0.16%	0.06%	0.06%	0.00%
Bacilli	1.31%	3.71%	3.15%	1.36%	0.96%	3.58%	0.60%	4.13%	2.67%	1.40%	23.59%	7.46%	27.42%
Clostridia	0.12%	0.41%	0.52%	0.18%	2.03%	0.30%	0.09%	0.58%	0.29%	0.13%	2.05%	0.80%	1.52%
Gemmatimonadetes	2.01%	2.13%	2.52%	2.06%	1.95%	2.52%	3.36%	1.98%	1.62%	1.90%	0.84%	1.42%	1.59%
Halanaerobiia	0.00%	0.18%	0.00%	0.00%	0.48%	0.00%	0.00%	0.00%	0.05%	0.00%	0.00%	0.00%	0.00%
Latescibacteria	0.10%	0.52%	0.42%	0.14%	0.49%	0.38%	0.13%	0.70%	0.79%	0.67%	0.27%	1.33%	0.79%
Latescibacterota	0.61%	2.37%	2.69%	4.02%	3.82%	1.72%	2.65%	1.81%	2.30%	2.12%	1.10%	1.27%	1.85%
Methylomirabilia	4.65%	4.39%	6.62%	5.86%	4.84%	6.68%	6.49%	5.48%	5.43%	4.81%	2.02%	4.32%	3.60%
Myxococcia	0.11%	0.34%	0.17%	0.14%	0.15%	0.09%	0.20%	0.13%	0.24%	0.03%	0.18%	0.19%	0.07%
Polyangia	0.98%	1.45%	1.19%	1.98%	1.22%	0.67%	0.72%	1.13%	1.57%	1.55%	1.13%	1.87%	1.00%
Nitrospiria	2.54%	1.74%	1.02%	1.47%	1.31%	1.10%	2.18%	0.65%	1.22%	1.48%	0.47%	0.96%	0.52%
Phycisphaerae	0.63%	0.74%	0.73%	0.74%	1.38%	0.72%	0.70%	0.80%	0.84%	0.98%	0.43%	0.51%	0.46%
Planctomycetes	5.09%	5.47%	6.02%	5.71%	7.91%	4.78%	3.22%	4.75%	5.64%	6.38%	2.55%	5.24%	3.11%
Alphaproteobacteria	8.23%	7.80%	7.87%	7.15%	5.56%	9.26%	8.33%	7.89%	8.85%	8.74%	5.05%	10.80%	6.42%
Gammaproteobacteria	12.56%	15.61%	11.03%	10.88%	8.44%	9.30%	13.97%	10.09%	12.05%	11.98%	11.14%	7.75%	7.34%
Verrucomicrobiae	4.54%	5.99%	5.38%	4.83%	3.84%	4.56%	3.65%	5.77%	7.17%	7.20%	1.72%	4.33%	2.89%
	100.00%	100.00%	100.00%	100.00%	100.00%	100.00%	100.00%	100.00%	100.00%	100.00%	100.00%	100.00%	100.00%

Supplementary Table 3: Simpsons index and inverse Simpsons index as a measure of microbial community diversity.

Samples from the mid-level	Simpsons diversity	Inverse Simpsons	Samples from the lower level	Simpsons diversity	Inverse Simpsons
**1 Roman Glass**	0.996074	1.003942	**1 Roman Glass**	0.99685	1.00316
**2 Med**	0.996946	1.003064	**2 Med**	0.997266	1.002742
**3 Hangleton**	0.997261251	1.00274627	**3 Hangleton**	0.995351724	1.004669983
**4 Plate Glass**	0.982468116	1.017844735	**4 Plate Glass**	0.997454762	1.002551732
**5 Plate Glass**	0.997669	1.002336	**5 Plate Glass**	0.99729	1.002718
**6 E glass**	0.997156672	1.002851436	**6 E glass**	0.997692029	1.00231331
**7 Borosilicate**	0.998071	1.001933	**7 Borosilicate**	0.99790039	1.002104027
**8 Soda Optical**	0.997709757	1.0022955	**8 Soda Optical**	0.996995	1.003015
**9 Lead Optical**	0.997296969	1.002710357	**9 Lead Optical**	0.997340506	1.002666586
**Limestone**	0.997982631	1.002021447	**Limestone**	0.997982631	1.002021447
**Mid soil 1**	0.996226	1.003789	**Lower soil 1**	0.98609672	1.01409931
**Mid soil 2**	0.517201	1.933485	**Lower soil 2**	0.99696981	1.0030394
**Mid soil 3**	0.997033	1.002975	**Lower soil 3**	0.986533628	1.013650191

Supplementary Table 4: Results by species of 16S rRNA analysis on glass samples from the mid-earthworks level listing those known to be capable of Fe(III) reduction.

Species	Confid ence	Soda Lime Optical	Lead Optical	E-Glass	Plate glass Unpolished	Hangleton Linen smoother	Medieva l	Borosilicate	Roman	Plate glass polished
d__Bacteria; p__Acidobacteriota; c__Acidobacteriae; o__Acidobacteriae; f__Acidobacteriae; g__Paludibaculum; s__metagenome	0.99	0.08%	0.04%	0.08%	0.09%	0.05%	0.05%	0.00%	0.00%	0.00%
d__Bacteria; p__Acidobacteriota; c__Acidobacteriae; o__Acidobacteriae; f__Acidobacteriae; g__Paludibaculum; s__uncultured_bacterium	0.83	0.02%	0.02%	0.00%	0.00%	0.00%	0.02%	0.05%	0.02%	0.00%
d__Bacteria; p__Acidobacteriota; c__Holophagae; o__Holophagales; f__Holophagaceae; g__Geothrix; s__uncultured_bacterium	0.93	0.00%	0.01%	0.00%	0.03%	0.00%	0.00%	0.00%	0.00%	0.00%
d__Bacteria; p__Desulfobacterota; c__Desulfuromonadia	1.00	0.00%	0.00%	0.00%	0.00%	0.00%	0.01%	0.04%	0.00%	0.02%
d__Bacteria; p__Desulfobacterota; c__Desulfuromonadia; o__Desulfuromonadia; f__Geoalkalibacteraceae; g__Geoalkalibacter	1.00	0.00%	0.00%	0.32%	0.00%	0.00%	0.00%	0.00%	0.00%	0.00%
d__Bacteria; p__Desulfobacterota; c__Desulfuromonadia; o__Desulfuromonadia; f__Geoalkalibacteraceae; g__Geoalkalibacter; s__uncultured_bacterium	0.70	0.05%	0.00%	0.00%	6.83%	0.00%	0.08%	0.01%	0.00%	0.02%
d__Bacteria; p__Desulfobacterota; c__Desulfuromonadia; o__Geobacterales; f__Geobacteraceae; g__Geobacter	1.00	0.01%	0.01%	0.02%	0.00%	0.00%	0.00%	0.02%	0.00%	0.02%
d__Bacteria; p__Myxococcota; c__Myxococcia; o__Myxococcales; f__Anaeromyxobacteraceae; g__Anaeromyxobacter	0.90	0.00%	0.00%	0.00%	0.00%	0.00%	0.00%	0.00%	0.00%	0.01%
d__Bacteria; p__NB1-j; c__NB1-j; o__NB1-j; f__NB1-j; g__NB1-j; s__uncultured_Desulfuromonadaceae	1.00	0.29%	0.16%	0.05%	0.21%	0.11%	0.20%	0.17%	0.29%	0.17%
d__Bacteria; p__Proteobacteria; c__Gammaproteobacteria; o__Gammaproteobacteria_Incertae_Sedis; f__Unknown_Family; g__Acidibacter	1.00	0.36%	0.32%	0.18%	0.32%	0.30%	0.08%	0.42%	0.13%	0.28%
d__Bacteria; p__Proteobacteria; c__Gammaproteobacteria; o__Gammaproteobacteria_Incertae_Sedis; f__Unknown_Family; g__Acidibacter	1.00	0.19%	0.17%	0.33%	0.29%	0.00%	0.06%	0.20%	0.16%	0.27%
d__Bacteria; p__Proteobacteria; c__Gammaproteobacteria; o__Gammaproteobacteria_Incertae_Sedis; f__Unknown_Family; g__Acidibacter	1.00	0.00%	0.00%	0.00%	0.36%	0.00%	0.00%	0.00%	0.00%	0.00%
d__Bacteria; p__Proteobacteria; c__Gammaproteobacteria; o__Gammaproteobacteria_Incertae_Sedis; f__Unknown_Family; g__Acidibacter	1.00	0.00%	0.00%	0.00%	0.00%	0.00%	0.00%	0.03%	0.00%	0.00%
d__Bacteria; p__Proteobacteria; c__Gammaproteobacteria; o__Gammaproteobacteria_Incertae_Sedis; f__Unknown_Family; g__Acidibacter; s__uncultured_bacterium	0.86	0.00%	0.00%	0.00%	0.00%	0.00%	0.00%	0.05%	0.06%	0.00%
d__Bacteria; p__Proteobacteria; c__Gammaproteobacteria; o__Gammaproteobacteria_Incertae_Sedis; f__Unknown_Family; g__Acidibacter; s__uncultured_proteobacterium	0.91	0.06%	0.00%	0.00%	0.00%	0.00%	0.00%	0.04%	0.04%	0.00%

Supplementary Table 5: Results by species of 16S rRNA analysis on glass samples from the lower-earthworks level listing those known to be capable of Fe(III) reduction.

Species	Confidence	Soda lime optical	Lead optical	e-glass	Plate Unpolished	Plate polished	Medieval	Hangleton Linen Smoother	Roman	Borosilicate
d__Bacteria; p__Acidobacteriota; c__Acidobacteriae; o__Acidobacteriae; f__Acidobacteriae; g__Paludibaculum; s__metagenome	0.99	0.00%	0.00%	0.00%	0.04%	0.00%	0.00%	0.00%	0.00%	0.00%
d__Bacteria; p__Acidobacteriota; c__Acidobacteriae; o__Acidobacteriae; f__Acidobacteriae; g__Paludibaculum; s__uncultured_bacterium	0.83	0.04%	0.00%	0.00%	0.00%	0.00%	0.00%	0.00%	0.00%	0.00%
d__Bacteria; p__Acidobacteriota; c__Holophagae; o__Holophagales; f__Holophagaceae; g__Geothrix; s__uncultured_bacterium	0.93	0.01%	0.00%	0.01%	0.00%	0.03%	0.02%	0.03%	0.00%	0.01%
d__Bacteria; p__Desulfobacterota; c__Desulfuromonadia	1.00	0.04%	0.08%	0.04%	0.09%	0.07%	0.13%	0.20%	0.15%	0.09%
d__Bacteria; p__Desulfobacterota; c__Desulfuromonadia; o__Desulfuromonadia; f__Geoalkalibacteraceae; g__Geoalkalibacter; s__uncultured_bacterium	0.70	0.00%	0.00%	0.11%	0.00%	0.00%	0.00%	0.20%	0.00%	0.00%
d__Bacteria; p__Desulfobacterota; c__Desulfuromonadia; o__Geobacterales; f__Geobacteraceae	1.00	0.00%	0.02%	0.00%	0.00%	0.00%	0.06%	0.00%	0.08%	0.00%
d__Bacteria; p__Desulfobacterota; c__Desulfuromonadia; o__Geobacterales; f__Geobacteraceae; g__Geobacter	1.00	0.00%	0.03%	0.00%	0.00%	0.05%	0.06%	0.00%	0.07%	0.03%
d__Bacteria; p__Desulfobacterota; c__Desulfuromonadia; o__Geobacterales; f__Geobacteraceae; g__Geobacter	1.00	0.00%	0.00%	0.03%	0.00%	0.00%	0.00%	0.00%	0.04%	0.03%
d__Bacteria; p__Latescibacterota; c__Latescibacterota; o__Latescibacterota; f__Latescibacterota; g__Latescibacterota; s__uncultured_Pelobacter	1.00	0.00%	0.00%	0.02%	0.00%	0.00%	0.00%	0.00%	0.00%	0.00%
d__Bacteria; p__Latescibacterota; c__Latescibacterota; o__Latescibacterota; f__Latescibacterota; g__Latescibacterota; s__uncultured_Pelobacter	1.00	0.00%	0.00%	0.05%	0.04%	0.00%	0.00%	0.04%	0.00%	0.00%
d__Bacteria; p__Myxococcota; c__Myxococcia; o__Myxococcales; f__Anaeromyxobacteraceae; g__Anaeromyxobacter	0.90	0.00%	0.05%	0.00%	0.00%	0.00%	0.00%	0.00%	0.00%	0.00%
d__Bacteria; p__Myxococcota; c__Myxococcia; o__Myxococcales; f__Anaeromyxobacteraceae; g__Anaeromyxobacter; s__uncultured_bacterium	0.99	0.00%	0.00%	0.00%	0.00%	0.13%	0.00%	0.00%	0.00%	0.00%
d__Bacteria; p__NB1-j; c__NB1-j; o__NB1-j; f__NB1-j; g__NB1-j; s__uncultured_Desulfuromonadaceae	1.00	0.29%	0.00%	0.16%	0.11%	0.06%	0.12%	0.03%	0.11%	0.24%
d__Bacteria; p__NB1-j; c__NB1-j; o__NB1-j; f__NB1-j; g__NB1-j; s__uncultured_Desulfuromonadaceae	1.00	0.00%	0.11%	0.00%	0.00%	0.00%	0.00%	0.00%	0.00%	0.12%
d__Bacteria; p__Proteobacteria; c__Gammaproteobacteria; o__Gammaproteobacteria_Incertae_Sedis; f__Unknown_Family; g__Acidibacter	1.00	0.09%	0.07%	0.08%	0.10%	0.00%	0.09%	0.00%	0.06%	0.14%
d__Bacteria; p__Proteobacteria; c__Gammaproteobacteria; o__Gammaproteobacteria_Incertae_Sedis; f__Unknown_Family; g__Acidibacter	1.00	0.09%	0.16%	0.00%	0.03%	0.28%	0.06%	0.06%	0.05%	0.10%

Supplementary Table 6: Results by species of 16S rRNA analysis on sediment and limestone samples from the mid- and lower-earthworks level listing those known to be capable of Fe(III) reduction.

Species	Confidence	Limestone	Lower soil	Lower soil	Lower soil	Mid section	Mid section	Mid section
d__Bacteria; p__Acidobacteriota; c__Acidobacteriae; o__Acidobacteriae; f__Acidobacteriae; g__Paludibaculum; s__metagenome	0.99	0.06%	0.00%	0.00%	0.00%	0.00%	0.00%	0.00%
d__Bacteria; p__Acidobacteriota; c__Acidobacteriae; o__Acidobacteriae; f__Acidobacteriae; g__Paludibaculum; s__uncultured_bacterium	0.83	0.03%	0.00%	0.00%	0.00%	0.00%	0.00%	0.00%
d__Bacteria; p__Desulfobacterota; c__Desulfuromonadia	1.00	0.04%	0.24%	0.19%	0.43%	0.03%	0.00%	0.00%
d__Bacteria; p__Desulfobacterota; c__Desulfuromonadia; o__Desulfuromonadia; f__Geoalkalibacteraceae; g__Geoalkalibacter; s__uncultured_bacterium	0.70	0.00%	0.00%	0.00%	0.00%	0.00%	4.55%	0.00%
d__Bacteria; p__Desulfobacterota; c__Desulfuromonadia; o__Geobacterales; f__Geobacteraceae	1.00	0.00%	0.19%	0.11%	0.09%	0.00%	0.00%	0.00%
d__Bacteria; p__Desulfobacterota; c__Desulfuromonadia; o__Geobacterales; f__Geobacteraceae; g__Geobacter	1.00	0.03%	0.11%	0.07%	0.20%	0.04%	0.00%	0.03%
d__Bacteria; p__Desulfobacterota; c__Desulfuromonadia; o__Geobacterales; f__Geobacteraceae; g__Geobacter	1.00	0.00%	0.00%	0.04%	0.06%	0.00%	0.00%	0.00%
d__Bacteria; p__Firmicutes; c__Desulfitobacteriia; o__Desulfitobacteriales; f__Desulfitobacteriaceae; g__Desulfitobacterium	1.00	0.00%	0.00%	0.00%	0.00%	0.00%	0.00%	0.24%
d__Bacteria; p__Latescibacterota; c__Latescibacterota; o__Latescibacterota; f__Latescibacterota; g__Latescibacterota; s__uncultured_Pelobacter	1.00	0.03%	0.00%	0.00%	0.00%	0.00%	0.00%	0.00%
d__Bacteria; p__Latescibacterota; c__Latescibacterota; o__Latescibacterota; f__Latescibacterota; g__Latescibacterota; s__uncultured_Pelobacter	1.00	0.01%	0.00%	0.00%	0.00%	0.00%	0.00%	0.00%
d__Bacteria; p__Myxococcota; c__Myxococcia; o__Myxococcales; f__Anaeromyxobacteraceae; g__Anaeromyxobacter	0.90	0.02%	0.00%	0.00%	0.00%	0.00%	0.00%	0.00%
d__Bacteria; p__NB1-j; c__NB1-j; o__NB1-j; f__NB1-j; g__NB1-j; s__uncultured_Desulfuromonadaceae	1.00	0.17%	0.00%	0.00%	0.07%	0.14%	0.00%	0.15%
d__Bacteria; p__NB1-j; c__NB1-j; o__NB1-j; f__NB1-j; g__NB1-j; s__uncultured_Desulfuromonadaceae	1.00	0.00%	0.16%	0.09%	0.00%	0.00%	0.00%	0.00%
d__Bacteria; p__Proteobacteria; c__Gammaproteobacteria; o__Gammaproteobacteria_Incertae_Sedis; f__Unknown_Family; g__Acidibacter	1.00	0.19%	0.00%	0.08%	0.11%	0.17%	0.06%	0.14%
d__Bacteria; p__Proteobacteria; c__Gammaproteobacteria; o__Gammaproteobacteria_Incertae_Sedis; f__Unknown_Family; g__Acidibacter	1.00	0.13%	0.04%	0.06%	0.00%	0.45%	0.00%	0.09%

Supplementary Table 7: Results by species of 16S rRNA analysis on glass samples from the mid-earthworks level listing those known to be capable of sulphate reduction.

Species	Confidence	Soda Lime Optical	Lead Optical	E-Glass	Plate glass Unpolished	Hangleton Linen smoother	Medieval	Borosilicate	Roman	Plate glass polished
d__Bacteria; p__Desulfobacterota; c__Desulfuromonadia; o__PB19; f__PB19; g__PB19; s__metagenome	1.00	0.00%	0.00%	0.00%	0.00%	0.00%	0.00%	0.00%	0.00%	0.03%
d__Bacteria; p__Desulfobacterota; c__uncultured; o__uncultured; f__uncultured; g__uncultured	0.98	0.23%	0.00%	0.00%	0.00%	0.38%	0.00%	0.00%	0.06%	0.00%
d__Bacteria; p__Desulfobacterota; c__uncultured; o__uncultured; f__uncultured; g__uncultured	0.87	0.13%	0.09%	0.00%	0.17%	0.04%	0.00%	0.08%	0.00%	0.00%
d__Bacteria; p__Desulfobacterota; c__uncultured; o__uncultured; f__uncultured; g__uncultured	0.99	0.03%	0.00%	0.00%	0.00%	0.00%	0.00%	0.00%	0.00%	0.00%
d__Bacteria; p__Desulfobacterota; c__uncultured; o__uncultured; f__uncultured; g__uncultured	0.95	0.00%	0.00%	0.17%	0.00%	0.00%	0.00%	0.00%	0.00%	0.00%
d__Bacteria; p__Desulfobacterota; c__uncultured; o__uncultured; f__uncultured; g__uncultured	0.84	0.15%	0.07%	0.00%	0.00%	0.00%	0.00%	0.00%	0.07%	0.00%
d__Bacteria; p__Desulfobacterota; c__uncultured; o__uncultured; f__uncultured; g__uncultured	0.95	0.00%	0.12%	0.00%	0.00%	0.00%	0.08%	0.00%	0.07%	0.00%
d__Bacteria; p__Desulfobacterota; c__uncultured; o__uncultured; f__uncultured; g__uncultured	0.94	0.00%	0.00%	0.00%	0.07%	0.00%	0.00%	0.00%	0.00%	0.00%
d__Bacteria; p__Desulfobacterota; c__uncultured; o__uncultured; f__uncultured; g__uncultured	0.87	0.00%	0.00%	0.00%	0.10%	0.00%	0.00%	0.00%	0.00%	0.11%
d__Bacteria; p__Desulfobacterota; c__uncultured; o__uncultured; f__uncultured; g__uncultured	0.78	0.00%	0.00%	0.00%	0.00%	0.00%	0.00%	0.00%	0.00%	0.04%
d__Bacteria; p__Desulfobacterota; c__uncultured; o__uncultured; f__uncultured; g__uncultured	0.93	0.00%	0.00%	0.00%	0.00%	0.00%	0.00%	0.00%	0.04%	0.00%
d__Bacteria; p__Desulfobacterota; c__uncultured; o__uncultured; f__uncultured; g__uncultured	0.98	0.00%	0.00%	0.00%	0.00%	0.00%	0.00%	0.00%	0.04%	0.02%
d__Bacteria; p__Desulfobacterota; c__uncultured; o__uncultured; f__uncultured; g__uncultured	1.00	0.00%	0.00%	0.00%	0.00%	0.00%	0.03%	0.00%	0.00%	0.00%
d__Bacteria; p__Desulfobacterota; c__uncultured; o__uncultured; f__uncultured; g__uncultured; s__delta_proteobacterium	0.93	0.00%	0.00%	0.03%	0.00%	0.00%	0.03%	0.00%	0.00%	0.00%
d__Bacteria; p__Desulfobacterota; c__uncultured; o__uncultured; f__uncultured; g__uncultured; s__metagenome	0.71	0.00%	0.00%	0.00%	0.00%	0.00%	0.14%	0.00%	0.00%	0.00%
d__Bacteria; p__Desulfobacterota; c__uncultured; o__uncultured; f__uncultured; g__uncultured; s__uncultured_Dongia	0.79	0.00%	0.00%	0.04%	0.00%	0.06%	0.00%	0.00%	0.04%	0.00%
d__Bacteria; p__Desulfobacterota; c__uncultured; o__uncultured; f__uncultured; g__uncultured; s__uncultured_proteobacterium	0.83	0.00%	0.10%	0.00%	0.10%	0.11%	0.00%	0.00%	0.00%	0.11%
d__Bacteria; p__Desulfobacterota; c__uncultured; o__uncultured; f__uncultured; g__uncultured; s__uncultured_proteobacterium	0.73	0.03%	0.00%	0.00%	0.00%	0.00%	0.19%	0.00%	0.14%	0.00%
d__Bacteria; p__Desulfobacterota; c__uncultured; o__uncultured; f__uncultured; g__uncultured; s__uncultured_sludge	0.70	0.00%	0.00%	0.12%	0.00%	0.00%	0.00%	0.00%	0.00%	0.00%
d__Bacteria; p__NB1-j; c__NB1-j; o__NB1-j; f__NB1-j; g__NB1-j; s__uncultured_Desulfuromonadaceae	1.00	0.29%	0.16%	0.05%	0.21%	0.11%	0.20%	0.17%	0.29%	0.17%

Supplementary Table 8: Results by species of 16S rRNA analysis on glass samples from the lower-earthworks level listing those known to be capable of sulphate reduction.

Species	Confidence	Soda lime optical	Lead optical	e-glass	Plate Unpolished	Plate polished	Medieval	Hangleton Linen Smoother	Roman	Borosilicate
d__Bacteria; p__Desulfobacterota; c__uncultured; o__uncultured; f__uncultured; g__uncultured	0.98	0.04%	0.12%	0.12%	0.27%	0.08%	0.00%	0.12%	0.10%	0.07%
d__Bacteria; p__Desulfobacterota; c__uncultured; o__uncultured; f__uncultured; g__uncultured	0.87	0.09%	0.00%	0.00%	0.00%	0.00%	0.00%	0.00%	0.00%	0.00%
d__Bacteria; p__Desulfobacterota; c__uncultured; o__uncultured; f__uncultured; g__uncultured	0.99	0.00%	0.00%	0.06%	0.00%	0.10%	0.13%	0.00%	0.11%	0.00%
d__Bacteria; p__Desulfobacterota; c__uncultured; o__uncultured; f__uncultured; g__uncultured	0.95	0.00%	0.00%	0.00%	0.00%	0.07%	0.00%	0.11%	0.00%	0.00%
d__Bacteria; p__Desulfobacterota; c__uncultured; o__uncultured; f__uncultured; g__uncultured	0.99	0.00%	0.00%	0.00%	0.00%	0.00%	0.00%	0.24%	0.09%	0.00%
d__Bacteria; p__Desulfobacterota; c__uncultured; o__uncultured; f__uncultured; g__uncultured	0.94	0.00%	0.00%	0.00%	0.00%	0.00%	0.00%	0.00%	0.00%	0.11%
d__Bacteria; p__Desulfobacterota; c__uncultured; o__uncultured; f__uncultured; g__uncultured	0.90	0.00%	0.00%	0.05%	0.00%	0.00%	0.00%	0.00%	0.00%	0.00%
d__Bacteria; p__Desulfobacterota; c__uncultured; o__uncultured; f__uncultured; g__uncultured	0.78	0.09%	0.08%	0.00%	0.00%	0.00%	0.00%	0.00%	0.00%	0.00%
d__Bacteria; p__Desulfobacterota; c__uncultured; o__uncultured; f__uncultured; g__uncultured	0.98	0.00%	0.00%	0.08%	0.00%	0.00%	0.00%	0.00%	0.00%	0.00%
d__Bacteria; p__Desulfobacterota; c__uncultured; o__uncultured; f__uncultured; g__uncultured; s__delta_proteobacterium	0.93	0.00%	0.00%	0.08%	0.00%	0.00%	0.00%	0.00%	0.00%	0.00%
d__Bacteria; p__Desulfobacterota; c__uncultured; o__uncultured; f__uncultured; g__uncultured; s__metagenome	0.71	0.06%	0.05%	0.00%	0.00%	0.00%	0.12%	0.00%	0.00%	0.09%
d__Bacteria; p__Desulfobacterota; c__uncultured; o__uncultured; f__uncultured; g__uncultured; s__metagenome	0.82	0.18%	0.00%	0.00%	0.00%	0.00%	0.00%	0.00%	0.00%	0.00%
d__Bacteria; p__Desulfobacterota; c__uncultured; o__uncultured; f__uncultured; g__uncultured; s__metagenome	0.73	0.00%	0.00%	0.00%	0.00%	0.14%	0.00%	0.00%	0.00%	0.00%
d__Bacteria; p__Desulfobacterota; c__uncultured; o__uncultured; f__uncultured; g__uncultured; s__Myxococcales_bacterium	0.74	0.00%	0.08%	0.06%	0.01%	0.00%	0.00%	0.00%	0.02%	0.02%
d__Bacteria; p__Desulfobacterota; c__uncultured; o__uncultured; f__uncultured; g__uncultured; s__uncultured_Dongia	0.79	0.02%	0.00%	0.00%	0.00%	0.00%	0.00%	0.00%	0.00%	0.00%
d__Bacteria; p__Desulfobacterota; c__uncultured; o__uncultured; f__uncultured; g__uncultured; s__uncultured_proteobacterium	0.83	0.25%	0.04%	0.14%	0.16%	0.12%	0.12%	0.00%	0.00%	0.07%
d__Bacteria; p__Desulfobacterota; c__uncultured; o__uncultured; f__uncultured; g__uncultured; s__uncultured_proteobacterium	0.73	0.24%	0.00%	0.00%	0.10%	0.00%	0.00%	0.00%	0.00%	0.00%
d__Bacteria; p__Desulfobacterota; c__uncultured; o__uncultured; f__uncultured; g__uncultured; s__uncultured_sludge	0.75	0.00%	0.00%	0.00%	0.00%	0.00%	0.15%	0.00%	0.09%	0.00%
d__Bacteria; p__Desulfobacterota; c__uncultured; o__uncultured; f__uncultured; g__uncultured; s__uncultured_sludge	0.71	0.00%	0.13%	0.00%	0.00%	0.00%	0.00%	0.00%	0.00%	0.00%
d__Bacteria; p__Desulfobacterota; c__uncultured; o__uncultured; f__uncultured; g__uncultured; s__uncultured_sludge	0.70	0.00%	0.00%	0.00%	0.00%	0.00%	0.00%	0.00%	0.07%	0.00%
d__Bacteria; p__Firmicutes; c__Desulfitobacteriia; o__Desulfitobacteriales; f__Desulfitobacteriaceae; g__Desulfosporosinus	1.00	0.00%	0.00%	0.00%	0.01%	0.08%	0.00%	0.00%	0.00%	0.00%
d__Bacteria; p__NB1-j; c__NB1-j; o__NB1-j; f__NB1-j; g__NB1-j; s__uncultured_Desulfuromonadaceae	1.00	0.29%	0.00%	0.16%	0.11%	0.06%	0.12%	0.03%	0.11%	0.24%
d__Bacteria; p__NB1-j; c__NB1-j; o__NB1-j; f__NB1-j; g__NB1-j; s__uncultured_Desulfuromonadaceae	1.00	0.00%	0.11%	0.00%	0.00%	0.00%	0.00%	0.00%	0.00%	0.12%

Supplementary Table 9: Results by species of 16S rRNA analysis on sediment and limestone samples from the mid- and lower earthworks level listing those known to be capable of sulphate reduction.

Species	Confidence	Limestone	Lower soil	Lower soil	Lower soil	Mid section	Mid section	Mid section
d__Bacteria; p__Desulfobacterota; c__Desulfobacteria; o__Desulfobacterales; f__Desulfobacteraceae	1.00	0.00%	0.27%	0.00%	0.00%	0.00%	0.00%	0.00%
d__Bacteria; p__Desulfobacterota; c__Desulfobacteria; o__Desulfobacterales; f__Desulfosarcinaceae	0.83	0.00%	0.29%	0.00%	0.00%	0.00%	0.00%	0.00%
d__Bacteria; p__Desulfobacterota; c__Desulfobacteria; o__Desulfobacterales; f__Desulfosarcinaceae	0.91	0.00%	0.00%	0.00%	0.00%	0.23%	0.00%	0.00%
d__Bacteria; p__Desulfobacterota; c__Desulfobacteria; o__Desulfobacterales; f__Desulfosarcinaceae; g__Desulfatitalea; s__uncultured_bacterium	1.00	0.00%	0.22%	0.00%	0.00%	0.00%	0.00%	0.00%
d__Bacteria; p__Desulfobacterota; c__Desulfobulbia; o__Desulfobulbales; f__Desulfocapsaceae; g__Desulfofustis; s__uncultured_bacterium	1.00	0.04%	0.18%	0.00%	0.00%	0.00%	0.00%	0.00%
d__Bacteria; p__Desulfobacterota; c__Desulfuromonadia; o__PB19; f__PB19; g__PB19; s__metagenome	1.00	0.00%	0.00%	0.00%	0.01%	0.00%	0.00%	0.00%
d__Bacteria; p__Desulfobacterota; c__uncultured; o__uncultured; f__uncultured; g__uncultured	0.98	0.00%	0.04%	0.13%	0.00%	0.00%	0.00%	0.00%
d__Bacteria; p__Desulfobacterota; c__uncultured; o__uncultured; f__uncultured; g__uncultured	0.87	0.00%	0.00%	0.00%	0.00%	0.12%	0.00%	0.00%
d__Bacteria; p__Desulfobacterota; c__uncultured; o__uncultured; f__uncultured; g__uncultured	0.99	0.00%	0.00%	0.14%	0.14%	0.00%	0.00%	0.00%
d__Bacteria; p__Desulfobacterota; c__uncultured; o__uncultured; f__uncultured; g__uncultured	0.99	0.07%	0.00%	0.00%	0.10%	0.00%	0.00%	0.00%
d__Bacteria; p__Desulfobacterota; c__uncultured; o__uncultured; f__uncultured; g__uncultured	0.95	0.00%	0.00%	0.00%	0.00%	0.00%	0.00%	0.00%
d__Bacteria; p__Desulfobacterota; c__uncultured; o__uncultured; f__uncultured; g__uncultured	0.99	0.00%	0.00%	0.00%	0.11%	0.00%	0.00%	0.00%
d__Bacteria; p__Desulfobacterota; c__uncultured; o__uncultured; f__uncultured; g__uncultured	0.87	0.00%	0.00%	0.00%	0.04%	0.00%	0.00%	0.00%
d__Bacteria; p__Desulfobacterota; c__uncultured; o__uncultured; f__uncultured; g__uncultured	0.78	0.00%	0.00%	0.00%	0.00%	0.00%	0.00%	0.00%
d__Bacteria; p__Desulfobacterota; c__uncultured; o__uncultured; f__uncultured; g__uncultured	0.93	0.04%	0.00%	0.00%	0.00%	0.00%	0.00%	0.00%
d__Bacteria; p__Desulfobacterota; c__uncultured; o__uncultured; f__uncultured; g__uncultured; s__delta_proteobacterium	0.93	0.00%	0.02%	0.10%	0.00%	0.00%	0.00%	0.02%
d__Bacteria; p__Desulfobacterota; c__uncultured; o__uncultured; f__uncultured; g__uncultured; s__Myxococcales_bacterium	0.74	0.00%	0.00%	0.01%	0.00%	0.00%	0.00%	0.00%
d__Bacteria; p__Desulfobacterota; c__uncultured; o__uncultured; f__uncultured; g__uncultured; s__uncultured_Dongia	0.79	0.00%	0.00%	0.00%	0.00%	0.00%	0.00%	0.04%
d__Bacteria; p__Desulfobacterota; c__uncultured; o__uncultured; f__uncultured; g__uncultured; s__uncultured_proteobacterium	0.83	0.16%	0.07%	0.19%	0.00%	0.05%	0.00%	0.00%
d__Bacteria; p__Desulfobacterota; c__uncultured; o__uncultured; f__uncultured; g__uncultured; s__uncultured_proteobacterium	0.73	0.00%	0.00%	0.00%	0.00%	0.00%	0.00%	0.00%
d__Bacteria; p__Desulfobacterota; c__uncultured; o__uncultured; f__uncultured; g__uncultured; s__uncultured_sludge	0.75	0.03%	0.00%	0.10%	0.00%	0.00%	0.00%	0.00%
d__Bacteria; p__Firmicutes; c__Desulfitobacteriia; o__Desulfitobacteriales; f__Desulfitobacteriaceae; g__Desulfosporosinus	1.00	0.00%	0.00%	0.08%	0.00%	0.00%	0.00%	0.00%
d__Bacteria; p__NB1-j; c__NB1-j; o__NB1-j; f__NB1-j; g__NB1-j; s__uncultured_Desulfuromonadaceae	1.00	0.17%	0.00%	0.00%	0.07%	0.14%	0.00%	0.15%
d__Bacteria; p__NB1-j; c__NB1-j; o__NB1-j; f__NB1-j; g__NB1-j; s__uncultured_Desulfuromonadaceae	1.00	0.00%	0.16%	0.09%	0.00%	0.00%	0.00%	0.00%

Supplementary Table 10: Results by order of 18S rRNA analysis on glass samples from the mid-earthworks level.

Total	100.00%	100.00%	100.00%	100.00%	100.00%	100.00%	100.00%	100.00%	100.00%
Total reads	190171	160674	136375	177383	221800	216683	160991	239767	219680
**Taxon**	**Soda Lime Optical**	**Lead Optical**	**E-Glass**	**Plate glass Unpolished**	**Hangleton Linen smoother**	**Medieval**	**Borosilicate**	**Roman**	**Plate glass polished**
Unclassified	36.11%	25.17%	26.25%	26.45%	46.84%	16.40%	36.44%	32.33%	39.06%
Nitrososphaeria	0.00%	0.00%	0.00%	0.00%	0.00%	0.00%	0.00%	0.00%	0.00%
Thermoplasmata	0.00%	0.00%	0.00%	0.00%	0.00%	0.00%	0.00%	0.00%	0.00%
Amoebozoa	0.00%	0.00%	0.00%	0.00%	0.00%	0.00%	0.00%	0.00%	0.00%
Discosea	0.60%	0.24%	0.00%	0.25%	0.08%	0.01%	0.16%	0.05%	0.29%
Tubulinea	0.41%	0.81%	0.00%	3.52%	0.41%	0.37%	0.72%	0.30%	1.79%
Clitellata	0.03%	0.00%	0.00%	0.00%	0.04%	0.26%	0.00%	0.28%	0.07%
Aphelidea	0.00%	0.00%	0.00%	0.00%	0.00%	0.00%	0.05%	0.00%	0.00%
Conoidasida	15.66%	4.96%	20.63%	9.79%	3.89%	3.62%	9.46%	10.01%	7.23%
Apusomonadidae	0.22%	0.47%	0.00%	0.00%	0.23%	0.00%	0.00%	0.00%	0.00%
Arachnida	0.00%	0.01%	0.00%	0.07%	0.60%	0.05%	0.00%	0.00%	0.09%
Chilopoda	0.00%	0.00%	0.00%	0.00%	0.00%	0.00%	0.00%	0.00%	0.00%
Diplopoda	0.00%	0.00%	0.00%	0.00%	0.00%	0.00%	0.00%	0.00%	0.06%
Ellipura	0.00%	0.03%	1.00%	0.00%	0.09%	0.05%	0.00%	0.00%	0.06%
Insecta	0.32%	0.00%	0.00%	0.00%	0.00%	0.00%	0.00%	0.00%	0.09%
Malacostraca	0.00%	0.13%	0.25%	0.01%	0.07%	2.48%	0.09%	0.00%	0.05%
Maxillopoda	0.00%	0.00%	0.00%	0.00%	0.00%	12.60%	0.00%	0.00%	0.00%
Dothideomycetes	2.66%	1.38%	0.00%	1.86%	0.90%	0.79%	2.21%	0.46%	1.39%
Eurotiomycetes	1.25%	0.73%	1.41%	0.39%	0.43%	0.58%	1.14%	0.95%	0.79%
Lecanoromycetes	0.00%	0.07%	0.00%	0.00%	0.00%	0.00%	0.00%	0.00%	0.00%
Leotiomycetes	1.83%	1.87%	0.10%	0.69%	0.44%	0.35%	3.82%	0.61%	1.55%
Pezizomycetes	0.16%	0.03%	0.00%	0.00%	0.00%	0.44%	0.00%	0.45%	0.17%
Pezizomycotina	0.00%	0.00%	0.00%	0.00%	0.00%	0.00%	0.00%	0.00%	0.00%
Saccharomycetes	0.17%	0.18%	0.00%	0.00%	0.04%	0.06%	0.00%	0.00%	0.19%
Sordariomycetes	0.00%	0.00%	0.00%	0.00%	0.00%	0.00%	0.00%	0.00%	0.00%
Taphrinomycetes	0.05%	0.03%	0.00%	0.00%	0.00%	0.00%	0.00%	0.00%	0.00%
Agaricomycetes	0.77%	0.66%	3.25%	1.55%	0.22%	0.93%	0.48%	0.03%	0.92%
Agaricostilbomycetes	0.00%	0.00%	0.00%	0.00%	0.00%	0.09%	0.00%	0.00%	0.00%
Cystobasidiomycetes	0.04%	0.05%	0.00%	0.00%	0.09%	0.11%	0.00%	0.00%	0.09%
Malasseziomycetes	0.16%	0.85%	1.15%	0.00%	0.17%	0.40%	0.00%	0.00%	4.35%
Microbotryomycetes	0.49%	0.00%	0.00%	0.00%	0.00%	0.00%	0.00%	0.00%	0.00%
Pucciniomycetes	0.00%	0.03%	0.00%	1.63%	0.00%	0.00%	0.00%	0.00%	0.00%
Tremellomycetes	0.00418	0.002614	0.001188	0.000671	0.000491	0.001034	0.006435	0	0.004083
Ustilaginomycetes	0.00%	0.00%	0.00%	0.00%	0.00%	0.00%	0.00%	0.00%	0.00%
Bicosoecida	0.00%	0.00%	0.00%	0.00%	0.00%	0.00%	0.00%	0.00%	0.00%
Breviatea	0.00%	0.00%	0.00%	0.00%	0.00%	0.00%	0.00%	0.00%	0.00%
Cavosteliida	0.00%	0.02%	0.00%	0.00%	0.00%	0.00%	0.00%	0.00%	0.00%
Centrohelida	0.11%	0.01%	0.00%	0.20%	0.00%	0.02%	0.09%	0.00%	0.19%
Cercomonadidae	3.65%	2.45%	3.12%	2.19%	3.68%	1.16%	2.39%	0.68%	4.22%
Cercozoa	0.32%	0.06%	0.00%	0.00%	0.05%	0.00%	0.00%	0.00%	0.00%
Glissomonadida	0.72%	0.27%	0.00%	0.16%	0.18%	0.26%	0.20%	0.02%	0.59%
Imbricatea	1.65%	0.82%	0.27%	1.00%	0.57%	0.10%	0.58%	0.60%	0.44%

Phytomyxea	2.14%	0.92%	3.58%	2.31%	1.42%	0.84%	1.27%	1.41%	2.58%
Thecofilosea	1.63%	0.42%	0.00%	1.01%	0.69%	0.05%	1.06%	0.00%	0.54%
Vampyrellidae	0.37%	0.00%	0.00%	0.00%	0.26%	0.02%	0.00%	0.00%	0.00%
Chlorophyceae	0.26%	0.09%	0.00%	0.00%	0.04%	0.00%	0.18%	0.00%	0.13%
Chlorophyta	0.25%	0.13%	0.00%	0.00%	0.00%	0.00%	0.00%	0.00%	0.00%
Trebouxiophyceae	0.52%	0.27%	0.00%	0.35%	0.08%	0.10%	0.00%	0.02%	0.13%
Ulvophyceae	0.00%	0.00%	0.00%	0.00%	0.00%	0.00%	0.00%	0.00%	0.00%
Chytridiomycetes	0.70%	0.05%	0.02%	0.00%	0.00%	0.01%	0.00%	0.00%	0.00%
Intramacronucleata	1.63%	0.84%	1.06%	0.00%	4.18%	3.61%	5.08%	1.17%	3.38%
Postciliodesmatophora	0	0	0	0	0	0	0	0	0
Cryptophyceae	0.00%	0.00%	0.00%	0.00%	0.00%	0.00%	0.00%	0.12%	0.00%
Bacillariophyceae	0.00%	0.00%	1.17%	0.00%	0.00%	0.00%	0.00%	0.00%	0.00%
Dictyostelia	0.00%	0.00%	0.00%	0.07%	0.00%	0.00%	0.00%	0.00%	0.00%
Dinophyceae	0.07%	0.00%	0.00%	0.00%	0.00%	0.00%	0.51%	0.00%	0.00%
Euglenida	0.03%	0.14%	0.00%	1.14%	1.33%	0.06%	0.28%	2.99%	0.11%
Kinetoplastea	0.32%	0.02%	0.38%	0.13%	0.16%	0.07%	0.17%	0.00%	0.14%
Rhodymeniophycidae	0.00%	0.00%	0.00%	0.00%	0.00%	0.00%	0.20%	0.00%	0.00%
Gracilipodida	0.79%	0.60%	0.00%	0.00%	0.09%	0.02%	0.90%	0.00%	0.43%
Haptophyta	0.00%	0.05%	0.00%	0.00%	0.00%	0.00%	0.22%	0.00%	0.00%
Tetramitia	0.00%	0.05%	0.00%	0.00%	0.00%	0.00%	0.00%	0.00%	0.07%
Choanoflagellida	0.00%	0.00%	0.00%	0.18%	0.00%	0.00%	0.00%	0.00%	0.00%
Ichthyosporea	0.21%	0.52%	0.00%	0.38%	0.37%	0.15%	0.77%	0.04%	0.51%
Hyphochytriomycetes	0.00%	0.00%	0.00%	0.00%	0.00%	0.00%	0.00%	0.00%	0.00%
Labyrinthulomycetes	0.00%	0.00%	0.00%	0.00%	0.00%	0.00%	0.00%	0.00%	0.00%
Microsporidia	0.00%	0.00%	0.00%	0.00%	0.00%	0.00%	0.00%	0.00%	0.00%
Gastropoda	0.00%	0.00%	0.88%	0.00%	0.00%	0.05%	0.13%	0.00%	0.00%
Glomeromycetes	0.06%	0.07%	2.46%	3.59%	0.25%	0.04%	0.17%	1.14%	0.04%
Incertae_Sedis	1.32%	1.11%	4.29%	23.52%	8.67%	9.62%	2.57%	7.15%	2.16%
Myxogastria	0.00%	0.00%	0.00%	0.00%	0.00%	0.00%	0.00%	0.00%	0.00%
Chromadorea	4.17%	0.27%	0.00%	0.36%	0.24%	1.76%	2.18%	4.65%	1.12%
Enoplea	0.00%	0.00%	4.50%	0.00%	0.00%	0.00%	8.24%	0.00%	0.00%
Chrysophyceae	1.96%	0.59%	0.00%	0.80%	0.30%	0.25%	2.38%	0.00%	0.63%
Xanthophyceae	0.00%	0.05%	0.00%	0.00%	0.00%	0.00%	0.00%	0.00%	0.00%
Peronosporomycetes	0.93%	1.01%	2.63%	0.52%	6.17%	1.00%	3.16%	1.58%	2.45%
Embryophyta	14.43%	50.09%	21.09%	15.58%	16.19%	41.09%	11.57%	32.57%	19.59%
Rhabditophora	0.05%	0.01%	0.00%	0.00%	0.01%	0.01%	0.00%	0.12%	1.84%
Perkinsidae	0.00%	0.00%	0.00%	0.00%	0.00%	0.00%	0.00%	0.00%	0.00%
Protosteliida	0.00%	0.00%	0.00%	0.00%	0.00%	0.00%	0.00%	0.21%	0.00%
Foraminifera	0.00%	0.00%	0.00%	0.00%	0.00%	0.00%	0.00%	0.00%	0.00%
Rigifilida	0.00%	0.00%	0.00%	0.00%	0.00%	0.00%	0.00%	0.00%	0.00%
Bdelloidea	0.00%	0.00%	0.00%	0.00%	0.00%	0.00%	0.00%	0.00%	0.00%
Monogononta	0.00%	0.16%	0.00%	0.00%	0.00%	0.00%	0.00%	0.00%	0.00%
Labyrinthulomycetes	0.20%	0.87%	0.37%	0.26%	0.50%	0.02%	0.23%	0.06%	0.01%
Schizoplasmodiida	0.17%	0.08%	0.00%	0.00%	0.00%	0.00%	0.27%	0.00%	0.06%

Supplementary Table 11: Results by order of 18S rRNA analysis on glass samples from the lower-earthworks level.

Total	100.00%	100.00%	100.00%	100.00%	100.00%	100.00%	100.00%	100.00%	100.00%
Total reads	271672	186416	127756	137330	84183	210220	166095	215545	271987
**Taxon**	**Soda lime optical**	**Lead optical**	**e-glass**	**Plate Unpolished**	**Plate polished**	**Medieval**	**Hangleton Linen Smoother**	**Roman**	**Borosilicate**
Unclassified	29.57%	21.09%	46.38%	26.52%	18.65%	31.34%	29.23%	24.92%	29.04%
Nitrososphaeria	0.05%	0.00%	0.00%	0.00%	0.00%	0.00%	0.00%	0.00%	0.00%
Thermoplasmata	0.00%	0.00%	0.00%	0.00%	0.00%	0.28%	0.00%	0.40%	0.07%
Amoebozoa	0.00%	0.00%	0.00%	0.00%	0.00%	0.00%	0.00%	0.00%	0.00%
Discosea	1.07%	0.32%	0.12%	0.63%	0.09%	0.64%	0.00%	0.29%	0.35%
Tubulinea	1.44%	0.77%	1.67%	1.26%	0.00%	1.86%	0.99%	0.52%	0.04%
Clitellata	0.00%	0.00%	0.36%	0.00%	0.00%	0.01%	0.00%	0.12%	0.00%
Aphelidea	0.00%	0.00%	0.00%	0.14%	0.00%	0.06%	0.00%	0.00%	0.00%
Conoidasida	1.53%	0.37%	1.25%	1.32%	0.02%	3.85%	0.01%	0.15%	0.06%
Apusomonadidae	0.00%	0.26%	0.00%	0.00%	0.00%	0.18%	1.19%	0.05%	0.00%
Arachnida	0.00%	0.00%	0.00%	0.00%	0.00%	0.00%	0.00%	0.00%	0.20%
Chilopoda	0.00%	0.00%	0.00%	0.00%	0.00%	0.00%	0.00%	0.00%	0.00%
Diplopoda	0.00%	0.00%	0.00%	0.00%	0.00%	0.00%	0.00%	0.00%	0.00%
Ellipura	0.00%	0.00%	0.06%	0.00%	0.00%	0.00%	0.00%	0.58%	5.76%
Insecta	0.00%	0.00%	0.00%	0.18%	0.00%	0.00%	0.00%	0.06%	0.10%
Malacostraca	0.00%	0.00%	0.00%	0.00%	0.00%	0.79%	0.00%	0.00%	0.00%
Maxillopoda	0.00%	0.00%	0.00%	0.00%	0.00%	0.00%	0.00%	0.00%	0.00%
Dothideomycetes	1.25%	0.42%	1.84%	3.07%	22.47%	0.45%	1.91%	2.03%	4.81%
Eurotiomycetes	0.08%	0.05%	0.56%	0.36%	0.00%	0.28%	1.18%	1.56%	1.46%
Lecanoromycetes	0.00%	0.00%	0.00%	0.06%	0.00%	0.02%	0.00%	0.13%	0.08%
Leotiomycetes	0.73%	1.81%	2.03%	1.48%	0.00%	2.81%	4.38%	2.42%	4.73%
Pezizomycetes	0.00%	0.24%	3.77%	0.00%	0.00%	1.58%	0.00%	3.39%	2.46%
Pezizomycotina	0.00%	0.00%	0.00%	0.00%	0.00%	0.00%	0.00%	0.00%	0.13%
Saccharomycetes	0.00%	0.00%	0.11%	0.00%	0.00%	0.04%	1.60%	0.14%	0.03%
Sordariomycetes	0.00%	0.00%	0.00%	0.06%	0.00%	0.01%	0.00%	0.01%	0.00%
Taphrinomycetes	0.00%	0.00%	0.00%	0.00%	0.00%	0.00%	0.00%	0.00%	0.24%
Agaricomycetes	0.11%	0.00%	4.15%	0.00%	0.74%	0.29%	0.00%	1.96%	1.50%
Agaricostilbomycetes	0.00%	0.00%	0.00%	0.00%	0.00%	0.00%	0.00%	0.00%	0.00%
Cystobasidiomycetes	0.00%	0.00%	0.00%	0.00%	0.00%	0.00%	0.00%	0.01%	0.05%
Malasseziomycetes	0.35%	0.00%	9.28%	2.45%	0.00%	0.62%	2.48%	0.97%	1.15%
Microbotryomycetes	0.00%	0.00%	0.00%	0.00%	0.00%	0.02%	0.00%	0.03%	0.39%
Pucciniomycetes	0.00%	0.00%	0.00%	0.00%	0.00%	0.00%	0.00%	0.00%	0.00%
Tremellomycetes	0.001307	0	0.004759	0.001223	0	0.001218	0.008634	0.00424	0.00075
Ustilaginomycetes	0.00%	0.00%	0.00%	0.00%	0.00%	0.00%	0.00%	0.06%	0.00%
Bicosoecida	0.00%	0.00%	0.00%	0.00%	0.00%	0.06%	0.00%	0.16%	0.00%
Breviatea	0.00%	0.00%	0.00%	0.00%	0.00%	0.00%	0.00%	0.00%	0.00%
Cavosteliida	0.00%	0.00%	0.00%	0.00%	0.00%	0.95%	0.00%	0.01%	0.02%
Centrohelida	0.00%	0.00%	0.00%	0.00%	0.00%	0.03%	0.00%	0.01%	0.69%
Cercomonadidae	1.12%	6.99%	4.92%	1.57%	4.49%	3.07%	0.79%	2.60%	2.69%
Cercozoa	0.00%	0.00%	0.13%	0.19%	0.00%	0.42%	0.00%	0.10%	0.21%
Glissomonadida	0.21%	0.00%	0.38%	0.82%	0.00%	0.32%	0.00%	0.49%	0.13%
Imbricatea	0.58%	0.35%	0.06%	0.62%	0.00%	1.61%	0.00%	1.51%	1.40%

Phytomyxea	0.00%	0.25%	0.00%	0.00%	0.00%	1.58%	0.00%	0.13%	0.23%
Thecofilosea	1.19%	2.02%	1.17%	0.19%	0.00%	2.84%	0.00%	2.02%	2.01%
Vampyrellidae	0.00%	1.49%	0.00%	1.83%	0.00%	2.46%	0.00%	0.47%	0.55%
Chlorophyceae	0.69%	0.94%	0.00%	0.00%	1.79%	1.70%	0.00%	0.78%	0.41%
Chlorophyta	0.00%	0.00%	0.00%	0.00%	0.00%	0.00%	0.00%	0.00%	0.00%
Trebouxiophyceae	0.00%	0.00%	0.00%	0.00%	0.00%	0.03%	0.90%	0.31%	0.34%
Ulvophyceae	0.00%	0.00%	0.00%	0.00%	0.00%	0.00%	0.00%	0.02%	0.00%
Chytridiomycetes	0.00%	0.02%	0.00%	0.00%	0.00%	0.05%	0.00%	0.03%	0.00%
Intramacronucleata	0.29%	1.59%	0.00%	0.38%	0.00%	3.10%	0.00%	0.57%	0.30%
Postciliodesmatophora	0	0	0	0	0	0	0	0	0
Cryptophyceae	0.00%	0.00%	0.00%	0.00%	0.00%	0.00%	0.00%	0.00%	0.00%
Bacillariophyceae	0.00%	0.00%	0.00%	0.00%	0.00%	0.04%	0.00%	0.02%	0.00%
Dictyostelia	0.00%	0.00%	0.00%	0.00%	0.00%	0.00%	0.00%	0.00%	0.00%
Dinophyceae	0.00%	0.00%	0.00%	0.00%	0.00%	0.00%	0.00%	0.00%	0.00%
Euglenida	0.15%	0.16%	0.45%	0.00%	0.00%	0.64%	0.00%	0.56%	0.00%
Kinetoplastea	0.22%	0.87%	0.12%	0.75%	0.00%	0.43%	0.25%	0.09%	0.33%
Rhodymeniophycidae	0.00%	0.00%	0.48%	0.00%	0.00%	0.03%	0.00%	0.00%	0.00%
Gracilipodida	0.19%	0.47%	4.97%	0.49%	0.00%	2.08%	0.00%	1.20%	2.37%
Haptophyta	0.00%	0.00%	0.00%	0.00%	0.00%	0.00%	0.00%	0.00%	0.00%
Tetramitia	0.00%	0.00%	0.18%	0.00%	0.02%	0.24%	0.49%	0.17%	0.13%
Choanoflagellida	0.00%	0.00%	0.00%	0.00%	0.00%	0.00%	0.00%	0.00%	0.00%
Ichthyosporea	0.00%	0.00%	0.00%	0.13%	0.00%	0.03%	0.00%	0.07%	0.29%
Hyphochytriomycetes	0.00%	0.00%	0.00%	0.00%	0.00%	0.00%	0.00%	0.00%	0.00%
Labyrinthulomycetes	0.00%	0.00%	0.00%	0.00%	0.00%	0.00%	0.00%	0.00%	0.00%
Microsporidia	0.00%	0.00%	0.00%	0.00%	0.00%	0.00%	0.00%	0.07%	0.00%
Gastropoda	0.06%	0.00%	0.23%	0.00%	0.00%	0.02%	0.00%	0.67%	0.25%
Glomeromycetes	0.44%	0.12%	0.11%	0.00%	0.00%	0.05%	0.00%	0.00%	0.26%
Incertae_Sedis	3.70%	2.20%	2.37%	4.95%	5.39%	5.37%	8.64%	4.69%	2.17%
Myxogastria	0.00%	0.00%	0.00%	0.01%	0.00%	0.03%	0.00%	0.01%	0.00%
Chromadorea	0.06%	0.70%	0.00%	0.13%	0.01%	8.80%	5.94%	0.23%	0.05%
Enoplea	0.00%	0.00%	0.00%	0.00%	0.00%	0.00%	0.00%	0.10%	0.00%
Chrysophyceae	0.76%	0.00%	0.82%	0.39%	0.00%	0.57%	1.48%	0.17%	1.89%
Xanthophyceae	0.00%	0.00%	0.00%	0.52%	0.00%	0.34%	0.00%	0.00%	0.00%
Peronosporomycetes	47.71%	1.43%	0.00%	0.60%	0.02%	5.07%	0.00%	1.94%	12.66%
Embryophyta	5.15%	52.88%	11.58%	48.78%	46.31%	12.23%	35.99%	40.27%	16.94%
Rhabditophora	0.00%	0.00%	0.00%	0.00%	0.00%	0.00%	0.00%	0.00%	0.08%
Perkinsidae	0.00%	0.00%	0.00%	0.00%	0.00%	0.00%	0.00%	0.00%	0.00%
Protosteliida	0.00%	0.00%	0.00%	0.00%	0.00%	0.00%	0.00%	0.00%	0.00%
Foraminifera	0.00%	0.00%	0.00%	0.00%	0.00%	0.00%	0.00%	0.00%	0.00%
Rigifilida	0.37%	0.00%	0.00%	0.00%	0.00%	0.00%	0.00%	0.04%	0.21%
Bdelloidea	0.29%	0.00%	0.00%	0.00%	0.00%	0.00%	0.00%	0.00%	0.00%
Monogononta	0.00%	1.13%	0.00%	0.00%	0.00%	0.00%	0.00%	0.00%	0.00%
Labyrinthulomycetes	0.52%	1.00%	0.00%	0.00%	0.00%	0.30%	1.69%	0.11%	0.37%
Schizoplasmodiida	0.00%	0.06%	0.00%	0.00%	0.00%	0.26%	0.00%	0.14%	0.28%

Supplementary Table 12: Results by order of 18S rRNA analysis on sediment samples from the limestone, mid-earthworks level and lower earthworks.

Total	100.00%	100.00%	100.00%	100.00%	100.00%	100.00%	100.00%
Total reads	283481	274555	282931	161787	173838	182607	163103
**Taxon**	Limestone	Low	Low	Low	Mid	Mid	Mid
Unclassified	24.24%	22.73%	31.31%	22.92%	25.77%	29.46%	23.24%
Nitrososphaeria	0.00%	0.00%	0.00%	0.00%	0.00%	0.00%	0.00%
Thermoplasmata	0.00%	0.05%	0.31%	0.00%	0.00%	0.00%	0.00%
Amoebozoa	0.00%	0.00%	0.04%	0.00%	0.00%	0.00%	0.00%
Discosea	0.14%	0.20%	0.48%	0.20%	1.90%	0.55%	0.06%
Tubulinea	0.70%	0.00%	0.82%	0.59%	0.00%	1.35%	0.47%
Clitellata	0.15%	0.00%	0.15%	0.30%	0.00%	0.00%	0.10%
Aphelidea	0.00%	0.00%	0.05%	0.13%	0.00%	0.00%	0.00%
Conoidasida	5.31%	3.20%	3.55%	13.42%	5.71%	14.15%	9.32%
Apusomonadidae	0.00%	0.00%	0.04%	0.00%	0.00%	0.00%	0.00%
Arachnida	0.00%	0.00%	0.00%	0.00%	15.33%	0.01%	7.31%
Chilopoda	0.11%	0.00%	0.00%	0.00%	0.00%	0.00%	0.00%
Diplopoda	0.32%	0.00%	0.00%	0.00%	0.00%	0.00%	0.08%
Ellipura	1.30%	0.00%	0.01%	0.06%	5.46%	1.34%	0.00%
Insecta	10.14%	0.00%	0.00%	0.00%	0.00%	0.00%	0.00%
Malacostraca	0.00%	0.00%	0.00%	0.00%	0.00%	0.00%	0.00%
Maxillopoda	0.18%	0.00%	0.00%	0.00%	0.00%	0.00%	0.00%
Dothideomycetes	1.72%	7.48%	2.62%	1.58%	1.27%	2.26%	1.97%
Eurotiomycetes	0.15%	0.26%	0.83%	1.00%	3.27%	1.00%	0.66%
Lecanoromycetes	0.05%	0.00%	0.05%	0.08%	0.00%	0.00%	0.00%
Leotiomycetes	1.31%	0.95%	3.04%	1.83%	1.01%	1.04%	1.11%
Pezizomycetes	0.09%	1.84%	1.21%	1.28%	0.00%	0.02%	0.36%
Pezizomycotina	0.15%	0.00%	0.00%	0.00%	0.78%	0.00%	0.00%
Saccharomycetes	0.13%	0.00%	0.08%	0.00%	0.00%	0.86%	0.30%
Sordariomycetes	0.02%	0.00%	0.01%	0.08%	0.00%	0.00%	0.00%
Taphrinomycetes	0.08%	0.00%	0.00%	0.00%	0.00%	0.00%	0.00%
Agaricomycetes	0.64%	0.97%	0.61%	0.15%	1.43%	0.24%	1.46%
Agaricostilbomycetes	0.00%	0.00%	0.00%	0.00%	0.00%	0.00%	0.00%
Cystobasidiomycetes	0.10%	0.00%	0.01%	0.00%	0.00%	0.00%	0.13%
Malasseziomycetes	0.00%	0.00%	0.00%	0.00%	0.71%	0.00%	0.00%
Microbotryomycetes	0.19%	0.00%	0.05%	0.00%	1.79%	0.00%	0.00%
Pucciniomycetes	0.00%	0.00%	0.00%	0.00%	0.00%	0.00%	0.00%
Tremellomycetes	0.001683	0	0.00094	0.001434	0.066286	0.000444	0
Ustilaginomycetes	0.00%	0.00%	0.00%	0.00%	0.00%	0.00%	0.00%
Bicosoecida	0.08%	0.00%	0.10%	0.00%	0.00%	0.00%	0.00%
Breviatea	0.03%	0.00%	0.00%	0.00%	0.00%	0.00%	0.00%
Cavosteliida	0.00%	0.00%	0.05%	0.01%	0.00%	0.00%	0.00%
Centrohelida	0.00%	0.00%	0.01%	0.00%	0.00%	0.00%	0.00%
Cercomonadidae	0.57%	3.21%	5.17%	1.89%	2.09%	2.36%	1.76%
Cercozoa	0.06%	0.00%	0.26%	0.35%	0.00%	0.32%	0.13%
Glissomonadida	0.00%	0.00%	0.49%	0.00%	0.00%	0.23%	0.09%
Imbricatea	0.31%	0.42%	1.10%	0.07%	0.00%	1.29%	0.77%
Phytomyxea	5.23%	0.93%	0.65%	0.62%	1.28%	1.29%	0.66%

Thecofilosea	0.87%	2.72%	2.67%	2.80%	1.10%	3.90%	1.68%
Vampyrellidae	0.37%	0.00%	1.39%	0.29%	0.00%	1.63%	0.21%
Chlorophyceae	0.00%	0.00%	1.22%	1.47%	0.00%	0.20%	1.41%
Chlorophyta	0.00%	0.00%	0.01%	0.00%	0.00%	0.35%	0.04%
Trebouxiophyceae	3.11%	0.00%	0.05%	0.25%	0.00%	0.50%	0.00%
Ulvophyceae	0.00%	0.00%	0.00%	0.00%	0.00%	0.00%	0.00%
Chytridiomycetes	0.00%	0.00%	0.03%	0.00%	0.00%	0.09%	0.01%
Intramacronucleata	2.32%	0.55%	3.10%	0.89%	1.95%	3.77%	2.59%
Postciliodesmatophora	0	0	0	0.002411	0	0	0.002275
Cryptophyceae	0.00%	0.00%	0.00%	0.00%	0.00%	0.00%	0.00%
Bacillariophyceae	0.05%	0.00%	0.01%	0.00%	0.00%	0.00%	0.00%
Dictyostelia	0.00%	0.00%	0.00%	0.00%	0.00%	0.00%	0.00%
Dinophyceae	0.00%	0.00%	0.00%	0.00%	0.00%	0.00%	0.00%
Euglenida	0.11%	0.60%	0.16%	0.14%	0.00%	0.02%	0.36%
Kinetoplastea	0.04%	0.04%	0.12%	0.08%	0.00%	0.10%	0.00%
Rhodymeniophycidae	0.00%	0.00%	0.04%	0.00%	0.00%	0.00%	0.00%
Gracilipodida	0.20%	0.77%	1.44%	2.20%	0.00%	1.32%	0.37%
Haptophyta	0.00%	0.00%	0.02%	0.00%	0.00%	0.00%	0.00%
Tetramitia	0.34%	0.62%	0.58%	0.35%	0.00%	0.00%	0.00%
Choanoflagellida	0.07%	0.00%	0.02%	0.00%	0.00%	0.00%	0.00%
Ichthyosporea	0.31%	0.00%	0.55%	0.05%	0.00%	0.02%	0.06%
Hyphochytriomycetes	0.00%	0.00%	0.00%	0.00%	0.00%	0.00%	0.00%
Labyrinthulomycetes	0.00%	0.00%	0.00%	0.00%	0.00%	0.00%	0.00%
Microsporidia	0.00%	0.00%	0.00%	0.00%	0.00%	0.00%	0.00%
Gastropoda	0.33%	0.00%	0.02%	0.09%	0.00%	0.00%	0.17%
Glomeromycetes	0.19%	0.14%	0.05%	0.08%	4.11%	0.35%	0.35%
Incertae_Sedis	1.61%	3.09%	3.16%	3.19%	1.40%	11.07%	4.24%
Myxogastria	0.02%	0.00%	0.00%	0.01%	0.00%	0.00%	0.00%
Chromadorea	0.19%	0.13%	1.50%	0.57%	1.32%	2.12%	8.12%
Enoplea	0.11%	2.97%	0.56%	0.00%	0.21%	0.00%	0.00%
Chrysophyceae	0.30%	0.00%	0.34%	0.25%	0.00%	0.00%	0.19%
Xanthophyceae	0.00%	0.00%	0.10%	0.16%	0.00%	0.26%	0.00%
Peronosporomycetes	1.57%	0.19%	1.57%	1.31%	2.53%	1.80%	1.74%
Embryophyta	33.81%	44.68%	27.58%	38.57%	12.94%	14.62%	28.11%
Rhabditophora	0.03%	0.00%	0.01%	0.00%	0.00%	0.00%	0.00%
Perkinsidae	0.00%	0.00%	0.00%	0.00%	0.00%	0.00%	0.00%
Protosteliida	0.00%	0.00%	0.00%	0.00%	0.00%	0.00%	0.00%
Foraminifera	0.00%	0.00%	0.00%	0.00%	0.00%	0.09%	0.00%
Rigifilida	0.00%	0.00%	0.01%	0.00%	0.00%	0.00%	0.00%
Bdelloidea	0.00%	0.00%	0.00%	0.00%	0.00%	0.00%	0.00%
Monogononta	0.03%	0.87%	0.00%	0.00%	0.00%	0.00%	0.00%
Labyrinthulomycetes	0.05%	0.38%	0.25%	0.17%	0.00%	0.00%	0.13%
Schizoplasmodiida	0.27%	0.00%	0.23%	0.13%	0.00%	0.00%	0.00%

Supplementary Table 13: atom % of elements measured by EPMA at points 1-14 indicated on the image below. Note that these data are semi-quantitive due to loss of Na, K during analysis. Results are given to 3 dp.

Element	K	S	Zn	O	Ca	Mo	Zr	P	Cl	Fe	Cr	Ni	Ti	Si	Na	Mg	F	Al	B	Total
1	0.607	0.049	0.002	63.261	2.470	0	0	0.221	0.370	0.242	0	0.043	0.547	20.174	0.114	0.008	0.090	11.804	0	100.001
2	0.912	0.005	0.000	48.000	4.780	0	0.010	0.655	0.358	0.674	0	0.016	0.003	38.197	0.160	0.011	0.022	6.196	0	99.999
3	1.620	0.024	0.017	13.164	10.103	0.002	0	0.781	0.550	1.078	0	0	0.044	62.053	0.228	0.028	0	10.306	0	99.999
4	0.592	0.010	0.004	54.853	4.091	0	0.002	0.408	0.173	0.865	0	0.009	0.022	32.357	0.047	0.020	0.092	6.452	0	99.999
5	2.291	0.037	0.000	30.059	9.681	0	0	0.450	0.432	2.121	0.017	0.024	0.046	47.395	0.313	0.027	0.067	7.040	0	99.999
6	0.772	0	0	40.254	5.210	0	0	0.430	0.359	0.854	0.023	0.002	0.047	44.450	0.100	0.022	0.033	7.443	0	99.999
7	0.608	0	0.021	37.934	4.818	0	0	0.410	0.307	1.030	0.014	0.024	0	46.568	0.023	0.022	0.048	8.172	0	99.999
8	0.912	0.010	0.020	32.393	6.592	0	0.010	0.441	0.316	1.417	0.034	0.052	0.042	49.176	0.085	0.032	0	8.468	0	99.999
9	0.525	0	0.003	38.562	5.355	0	0	0.403	0.250	1.016	0.019	0	0.046	46.063	0.046	0.024	0.143	7.543	0	100.000
10	1.326	0.029	0.014	38.562	8.193	0	0	0.507	0.445	1.793	0.03	0	0	52.394	0.387	0.032	0.140	9.004	0	99.999
11	1.385	0.017	0.019	25.704	8.207	0	0.018	0.393	0.323	1.466	0	0	0.049	50.685	0.119	0.026	0.020	7.189	0	100.000
12	1.079	0.002	0.015	50.249	4.901	0	0	0.220	0.239	0.902	0.008	0	0.001	36.928	0.123	0.002	0	5.330	0	100.000
13	0.388	0	0.007	51.438	3.498	0	0.005	0.153	0.205	0.621	0	0	0.024	37.410	0.037	0.015	0.061	6.141	0	100.000
14	1.209	0.018	0.012	0.759	10.186	0	0	0.065	0.135	1.879	0.012	0.021	0.016	76.107	0.215	0.098	0	9.269	0	100.000

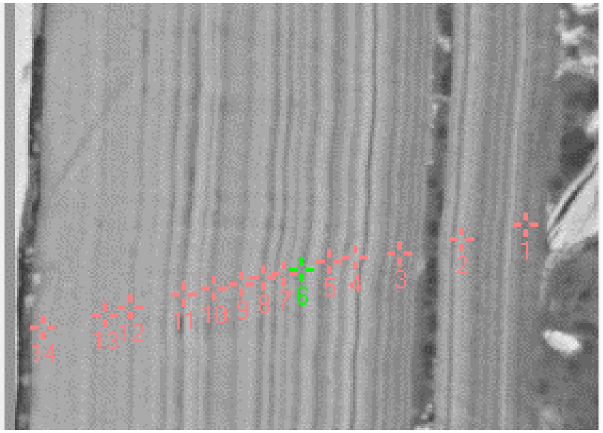

Supplement: Supplementary file 1 — Supplemental Material [file 41529_2025_571_MOESM1_ESM.pdf]
